# Fluorescence imaging of beta cell primary cilia

**DOI:** 10.3389/fendo.2022.1004136

**Published:** 2022-09-23

**Authors:** Zipeng A. Li, Jung Hoon Cho, Louis G. Woodhams, Jing W. Hughes

**Affiliations:** ^1^ Department of Medicine, Washington University School of Medicine, Saint Louis, MO, United States; ^2^ Department of Mechanical Engineering and Materials Science, Washington University McKelvey School of Engineering, Saint Louis, MO, United States

**Keywords:** Cilia, fluorescence, beta cell (β-cell), microscopy, live-cell

## Abstract

Primary cilia are slender cell-surface organelles that project into the intercellular space. In pancreatic beta cells, primary cilia coordinate a variety of cell responses including GPCR signaling, calcium influx, and insulin secretion, along with likely many underappreciated roles in islet development and differentiation. To study cilia function in islet biology, direct visualization of primary cilia by microscopic methods is often a necessary first step. Ciliary abundance, distribution, and morphology are heterogeneous among islet cells and are best visualized by fluorescence microscopy, the tools for which are readily accessible to most researchers. Here we present a collection of fluorescence imaging methods that we have adopted and optimized for the observation of primary cilia in mouse and human islets. These include conventional confocal microscopy using fixed islets and pancreas sections, live-cell imaging with cilia-targeted biosensors and probes, cilia motion recordings, and quantitative analysis of primary cilia waveform in the *ex vivo* environment. We discuss practical considerations and limitations of our approaches as well as new tools on the horizon to facilitate the observation of primary cilia in pancreatic islets.

## Introduction

Primary cilia are hair-like protrusions on the surface of most vertebrate cells. As a cellular antenna, primary cilia provide a physical platform for receiving and integrating molecular and mechanical signals to stimulate cellular responses (1–4). In the pancreas, primary cilia mediate diverse processes in the development, differentiation, and maintenance of tissue function, including cell proliferation, islet vascularization, hormone secretion, transcriptome regulation, and intercellular communication (5–13). Cilia defects disrupt energy homeostasis through central and peripheral metabolic pathways, and human ciliopathy syndromes as well as type II diabetes and metabolic disorders in general have been linked to monogenic ciliary changes (10, 14–18). All three major types of islet endocrine cells contain primary cilia, which in rodent and human islets are typically 5-10 μm in length and sub-micron in width (5, 8, 19–22). A number of key proteins are conserved among islet cilia and make easy immunostaining targets or can be labeled by fluorescent-tagged proteins for live- or fixed-cell imaging. These ciliary proteins reside in well-defined compartments including a centriole-derived basal body that anchors the cilium, a proximal transition zone for gating protein entry, and a microtubule-based axoneme with a dynamic distal tip specialized in signaling (23, 24). Functionally, primary cilia are conventionally classified as sensory, in contrast to motile cilia whose role is to beat in coordinated waves for mucociliary clearance and cell motility in specialized tissues (23, 25–27). This distinction between primary and motile cilia has recently become blurred, as sensory functions have been discovered in motile cilia and varying forms of motility have been identified in primary cilia (28–35). In pancreatic beta cells, primary cilia movement relies on glucose metabolism and intracellular energy production, while the movement itself is required for glucose-stimulated calcium entry and insulin release (35). Thus, primary cilia may not be strictly immotile as previously classified, and their movement contributes to their role as sensory organelles in the islet environment (5, 6, 9, 10, 19, 33, 34).

There has been growing interest in understanding the ciliary regulation of beta cell function. Key metabolic receptors are concentrated and uniquely accessible on the surface of primary cilia, providing attractive pharmacological targets. The first step and – often – barrier in studying islet cilia is the visualization of ciliary structures in intact pancreatic and islets, particularly in human specimens. While most of the current knowledge of islet cilia distribution, abundance, and morphology comes from fixed sliced tissues or cell lines using one or two axonemal markers, there is increasing need for visualizing multiple ciliary components in three-dimensional (3D) islet specimens as well as real-time observation of cilia dynamics in live cells. Therefore, imaging methods for primary cilia has become an increasingly hot topic of discussion for islet biologists who are working to understand the structure and function of these small organelles. This methods paper summarizes our current approaches to fluorescence imaging of primary cilia in mouse and human islets. We provide notes on specimen preparation, microscope settings, and practical tips for generating consistent and clean cilia images. Sample images are provided in the Results section along with discussions about antibody selection and imaging parameters, as well as limits and pitfalls of each approach. Our methods described herein are adaptable to various fixation and staining conditions and have been validated by collaborator labs, thus should prove broadly reproducible. Finally, while the images shown here were generated on two Zeiss LSM880 and LSM980 inverted confocal microscopes, our methods are compatible with a variety of imaging systems and can be combined with orthogonal imaging techniques such as intravital microscopy or correlative light and electron microscopy.

## Materials and methods

### Isolating and staining intact islets

Mouse pancreatectomy and islet isolations were performed using an established protocol (36, 37) with adherence to guidelines by The Washington University School of Medicine Institutional Animal Care and Use Committee. Intact islets were cultured in islet media containing RPMI supplemented with 10% FBS, 11 mM glucose, 20 mM HEPES, and antibiotics. Human islets from cadaveric donors were obtained from the NIDDK Integrated Islet Distribution Program (IIDP) or purchased commercially from Prodo Laboratories, Inc, with adherence to IRB guidelines of human specimen use.

**Figure 1:**
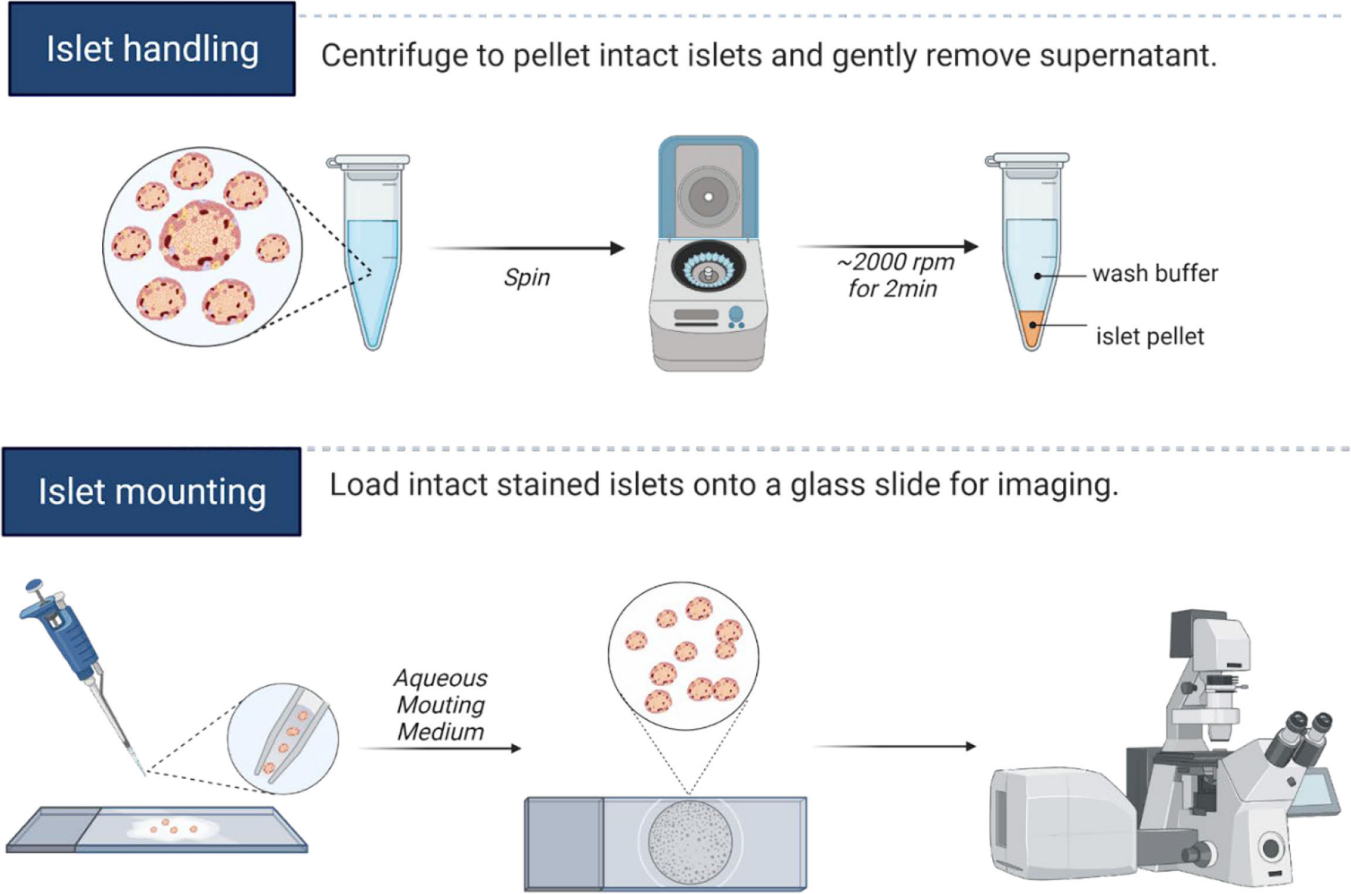
Islet handling tips for immunostaining. Isolated islets can be pelleted by centrifugation, and the entire staining protocol can be completed in microcentrifuge tubes. Care should be taken to not disrupt the islet pellet when aspirating wash fluids. We recommend leaving residual liquid above the islet pellet to prevent unintentional islet loss. For mounting whole islets, hand-transfer islets from low-adhesion staining tubes onto a glass slide, aspirate away excess liquids by pipette, then cover islets with 1-2 drops of mounting media and coverslip, taking care not to let islets dry out completely during the mounting process. A hydrophobic barrier pen may be useful in marking the islet boundary to prevent islet spread on the slide and to facilitate islet identification during imaging. Graphics created in BioRender.

Post-isolation or shipping arrival, mouse and human islets are cultured at least overnight in normoglycemia to allow recovery of morphology and function. Cilia length and number may be induced by serum starvation or by other alterations in culture conditions (38–40), but we typically avoid these manipulations if the goal is to capture physiologic cilia. For fixed imaging, between 20-50 whole islets per staining condition are hand-picked into 1.7 mL low-adhesion microcentrifuge tubes (VWR 490003-230), washed with PBS, then fixed with 4% paraformaldehyde (PFA) for 30 minutes and permeabilized with 0.3% Triton X-100 in PBS (PBST) for 10 minutes at room temperature (RT). After blocking with 0.3% PBST in CAS-Block buffer (Thermo Fisher 008120) for 1 hour at RT, islets are incubated for 48 hours at 4°C with primary antibodies diluted in blocking buffer. Islets are washed 5-6 times in PBS and incubated with secondary antibodies diluted in PBS at 4°C overnight. On the following day, islets are washed with PBS, and a quick 5-minute incubation in DAPI (5 µg/ml) provides nuclear counterstain. In all staining and washing steps, we find it helpful to pellet islets using a tabletop centrifuge (1000-2000 rpm for 2-5 minutes) and to gently aspirate wash solutions using gel loading pipette tips (Fisherbrand 02-707-181), leaving a 20-50 µL liquid residual to avoid unintentional islet loss. Stained islets are then transferred to glass slides by hand pipetting using low-retention tips coated with FBS solution beforehand to prevent islets from adhering, then covered with ProLong Gold Antifade Mountant (Thermo Fisher P36930) and stored at 4°C protected from light until imaging. A graphical depiction of islet handling and islet mounting workflow are presented in **Figure 1**.

### Whole-tissue staining using pancreas thin sections

Pancreas sections allow visualization of larger tissue areas and simultaneous examination of endocrine and non-endocrine structures. In contrast to isolated islets, pancreas tissue sections provide an intact surrounding and are less prone to preparation-induced damages to the islet structure. We typically prepare mouse or human pancreas sections by flash-freezing in optimal cutting temperature (OCT) compound and cut into cryosections, or by fixing in 4% PFA, preferably EM-grade (Electron Microscopy Sciences 15710), then serially dehydrating in 10-30% sucrose gradient solution for three consecutive days, and embedding and cutting as frozen thin sections into 5-10 µm thickness. Tissue staining require different incubation time than isolated islets, which should be optimized before each experiment. Thicker tissue sections (50-100 µm or more) may be used but will require optical clearing. For both islets and pancreas specimens, we recommend considering experimental and biological reproducibility and to use samples from donors of varying age and gender, when possible.

### Microscope settings and imaging resolution

Fixed stained islets or pancreas sections on glass slides are first visually examined using brightfield transmitted light or differential interference contrast (DIC). After selecting the target area for imaging, switch to fluorescence and excite with the appropriate lasers, keeping laser power to a minimum (<1%) to prevent photobleaching. Primary cilia are diminutive structures but can be easily recognized with clean staining and by a trained eye. For a whole-islet view, low-magnification objectives such as 20x air on the Zeiss LSM880 confocal system is sufficient and appropriate for capturing all cilia within an intact islet. Single-cilium images are best acquired with 63x or 100x water or oil immersion objectives, which produce superior high-resolution images especially when used in combination with the Airyscan detector (41). Three-dimensional islet volumes can be acquired at approximately 0.2-0.4 μm/pixel resolution, with recommended optical sectioning by 1 μm or sub-micron increments in axial z-dimension that would be sufficient for resolving cilia morphology. Spatial coordinates of primary cilia can be mapped in relation to the cell body and the z-depth from the surface of the coverslip. As with whole islets, 3D z-stacks are possible and advisable in pancreatic sections, even in sections as thin as 5-10 µm, as it allows potential capture of adjacent cell layers and visualization of the full length and trajectory of their cilia.

### Cilia morphometrics

Post-acquisition, whole-islet or pancreas images can be reconstructed in ImageJ and Imaris software, combining z-stacks or creating 3D rendering or maximal intensity projections. Quantitation of cilia number per high-power field (hpf) can be done by manual counting or by computer-assisted programs like ACDC and CiliaQ, open-source software or ImageJ plugins to automate cilia counting (42–45). While both these programs work well with 2D images to automate cilia/nuclei detection, CiliaQ is better suited for analyzing high dimensional images (3D-4D) and can capture morphometric parameters such as ciliary length, curvature, intensity, and orientation relative to the cell body and nucleus. Thresholds need to be applied when specifying automated cilia detection, and images should be manually reviewed to exclude non-ciliary signals such as cytosolic staining or nerve tissue. Segmenting cilia labels by cell identity markers will allow quantitation of cilia distribution on alpha/beta/delta cells as well as regional differences (peri-vascular vs non-vascular, innervated vs non-innervated) throughout the islet.

### Visualizing cilia in live islets

The primary cilium, contrary to canonical definition, is not static but constantly remodels and moves. This makes time-lapse imaging in live cells imperative to understand primary cilia dynamics in addition to their fixed distribution and morphology. To visualize live beta cell cilia, we generated a constitutive green fluorescent cilia reporter mouse (Ins1-Cre SSTR3-GFP^OFF^) that expresses GFP-tagged somatostatin receptor-3 (SSTR3) under the control of Ins1-Cre in islet beta cells (22, 35, 46). SSTR3 is a G protein-coupled receptor (GPCR) enriched on the membrane of beta cell cilia, making it ideal as a marker for cilia labeling and a functional mediator of paracrine somatostatin signaling (22, 47). Ins1-Cre SSTR3-GFP^OFF^ mice are viable and healthy, with normal islet function and secretory capacity, thus bearing no apparent ill effects of transgene expression. Intact islets from these reporter mice can be isolated and imaged *ex vivo*, where beta cell cilia stably and brightly express green fluorescence, and cilia waveform traces can be generated through time-lapse imaging and analyzed using a quantitative mathematical model (35). As beta cell cilia movement is modifiable by islet condition, ambient glucose, and ATP availability (35), as well as other environmental factors such as temperature, pH, and osmolarity, it is good practice to use fresh healthy islets preferably within 1-2 days of isolation, correctly balanced imaging buffers, and employ humidified and CO_2_-regulated imaging chambers in all live-cell cilia recording sessions. In general, prolonged time-lapse imaging in whole islets over extended time scales (minutes to hours) can introduce photobleaching and phototoxicity issues, as laser excitation can cause fluorophore decay and produce free radicals that damage cell health. We recommend proactively addressing these issues in experimental design, by minimizing laser power and illumination during image acquisition, limiting the time duration to only what is necessary to record the physiologic process, e.g. glucose-stimulated calcium flux or insulin secretion, and reducing sampling frequency, practices that will also help keep imaging file sizes manageable.

For native islets in non-transgenic animals and human donor islets, live-cell cilia labeling may be simply and rudimentarily achieved by using cell-permeant agents that bind membrane phospholipids or fluorescent lectins that bind sugar moieties on glycosylated proteins (48–50). This type if labeling, however, is not specific to cilia, as microvilli, nerves, and other membrane structures can also be detected (51, 52). The cell-permeable dye SiR-tubulin (Cytoskeleton CY-SC002) stains endogenous microtubules and is another useful proxy for cilia labeling, since tubulin is a major structural component of cilia. SiR-tubulin is a conjugated chemical of the bright fluorophore silicon rhodamine (SiR) and microtubule binding drug Docetaxel (53), which has the advantage of being in the far-red channel and can be used alongside green and red fluorescent proteins, also compatible with super-resolution microscopy (STED and SIM). In addition to strong ciliary labeling, SiR-tubulin does produce moderate amounts of fluorescence signal in the cell body, which can potentially mask overlapping cilia, making it necessary to apply threshold criteria when reviewing images and to select those cilia that are clearly labeled.

More specific live-cell cilia labeling may be achieved by using cilia-targeted fluorescent probes specific for second-messengers such as cAMP and Ca^2+^ (54–57). The green cADDis sensors were originally developed as a reporter for whole-cell cytosolic cAMP (58) and has since been modified *via* a fusion construct using 5HT6 to target cilia membrane expression (54) (Montana Molecular D0201G), making it a useful live-cell label for primary cilia. Commercially available cADDis can be purchased as pre-packaged viral vectors that can then be directly applied to cells and islets, making it easy to use and optimize. Post-infection in whole or partly dispersed islets, we generally allow cells to recover 24-48 hours in a 12-well low cell attachment plate (Thermo Fisher 174931) where islets can recover and re-aggregate before imaging. The cADDis sensor can also be used, as intended, to measure intra-ciliary cAMP activity, which is best done using the ratiometric version of the virus (Montana Molecular D0211). cADDis sensor response appears not to be affected by tagging of the 5HT6 receptor, as multiple ciliary proteins including Arl13b and SSTR3 have been targeted in this fashion without changing the sensor response (54). Similarly, cilia-targeted calcium probes such as 5HT6-GECO (Addgene 47499) (55, 59) can be used for the study of ciliary calcium dynamics while serving as a surrogate label for primary cilia. If greater ciliary specificity still is desired, one can introduce transient transgene expression of fluorescent-tagged proteins such as the ciliary GPCRs somatostatin receptor 3 (SSTR3) and 5-hydroxytryptamine receptor (5HT6), or ADP-ribosylation factor-like protein 13 B (Arl13b), a small GTPase enriched in the cilia membrane (60–62), using transfection or viral transduction approaches.

### Multiplex imaging of cilia and other beta cell dynamics

Primary cilia dynamics may be visualized in live cells in combination with other subcellular processes using multi-channel live imaging. These include cytosolic calcium and cAMP fluxes as aforementioned, whole-cell or localized metabolic events, insulin exocytosis, cell division, just to name a few. For both calcium and cAMP, subcellular dynamics of these messengers can be examined using cilia-targeted sensors alongside the cytosolic versions of the sensors. For cytoplasmic calcium, cell-permeant dyes such as Fura Red, Fluo-4, Calcium Orange, and Calbryte can be used on intact islets, or genetic calcium sensors such as GCaMP6 and GECO can be targeted to intact or partly dissociated islets *via* viral constructs, potentially in beta cell-specific manner, producing more stable fluorescence with greater signal-noise ratio than cell-permeant dyes (63, 64). Cytosolic cAMP can be monitored using a number of PKA- or EPAC-based sensors (54, 65, 66) including the whole-cell cADDis reporter [(Montana Molecular (58)]. Most calcium and cAMP sensors are single-color green or red, providing an opportunity to add another channel for cilia in dual-color live imaging. We have had success in combining cilia motion imaging in our Ins1-Cre SSTR3-GFP cilia reporter islets with whole-cell calcium imaging using Calbryte 590 AM (AAT Bioquest 20700). The simultaneous analysis of ciliary activity with cytoplasmic Ca^2+^ or cAMP fluxes and their correlation are computationally complex and is still a method that is being developed. We expect that these correlative results will help elucidate the sequence of cellular events that link glucose stimulation to beta cell secretion. Lastly, as an orthogonal approach to studying *ex vivo* islets, cilia and other beta cell dynamics can also be imaged in pancreatic slices which may be more faithful to *in vivo* physiology (67).

## Results and discussion

### Islet cilia distribution is differentially captured by 2D and 3D imaging

**Figure 2:**
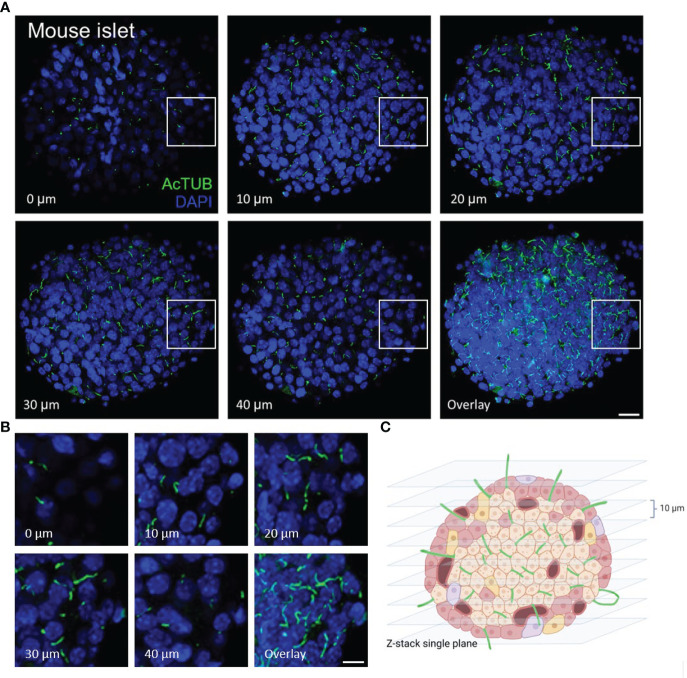
Serial z-plane capture of primary cilia in isolated islets. **(A)** Cross-section panels of cilia staining by acetylated α-tubulin (AcTUB, green) in an intact wildtype B6 mouse islet. The number and length of cilia vary depending on z-plane. Overlay shows cumulative cilia throughout the islet. Nuclei, DAPI (blue). Scale bar, 20 µm. **(B)** A cropped 40 x 40 x 20 µm volume of an islet interior showing cilia coursing through serial z-planes. Scale bar, 10 µm. **(C)** BioRender graphic illustration of typical z-stacks for imaging whole-islet cilia, combining vertical slices in 10 µm step size.

In whole islets, primary cilia can be visualized in single-plane view or in z-stacks (**Figure 2**). In our experience, most cells in mouse islets (up to 80-90%) and a significant portion of cells in human islets (up to 50-70%) are ciliated under basal culture conditions i.e. without serum starvation. Islet primary cilia have conserved protein components, including axonemal markers acetylated α-tubulin, Arl13b, and polyglutamylated tubulin which have proved reliable for cilia labeling in both human and mouse islets. In general for fluorescence studies, we recommend reserving the brightest channel for the least abundant signal. Therefore we typically stain cilia using secondary antibodies with 488 nm or 568 nm excitation wavelengths so to facilitate their visualization.

**Figure 3:**
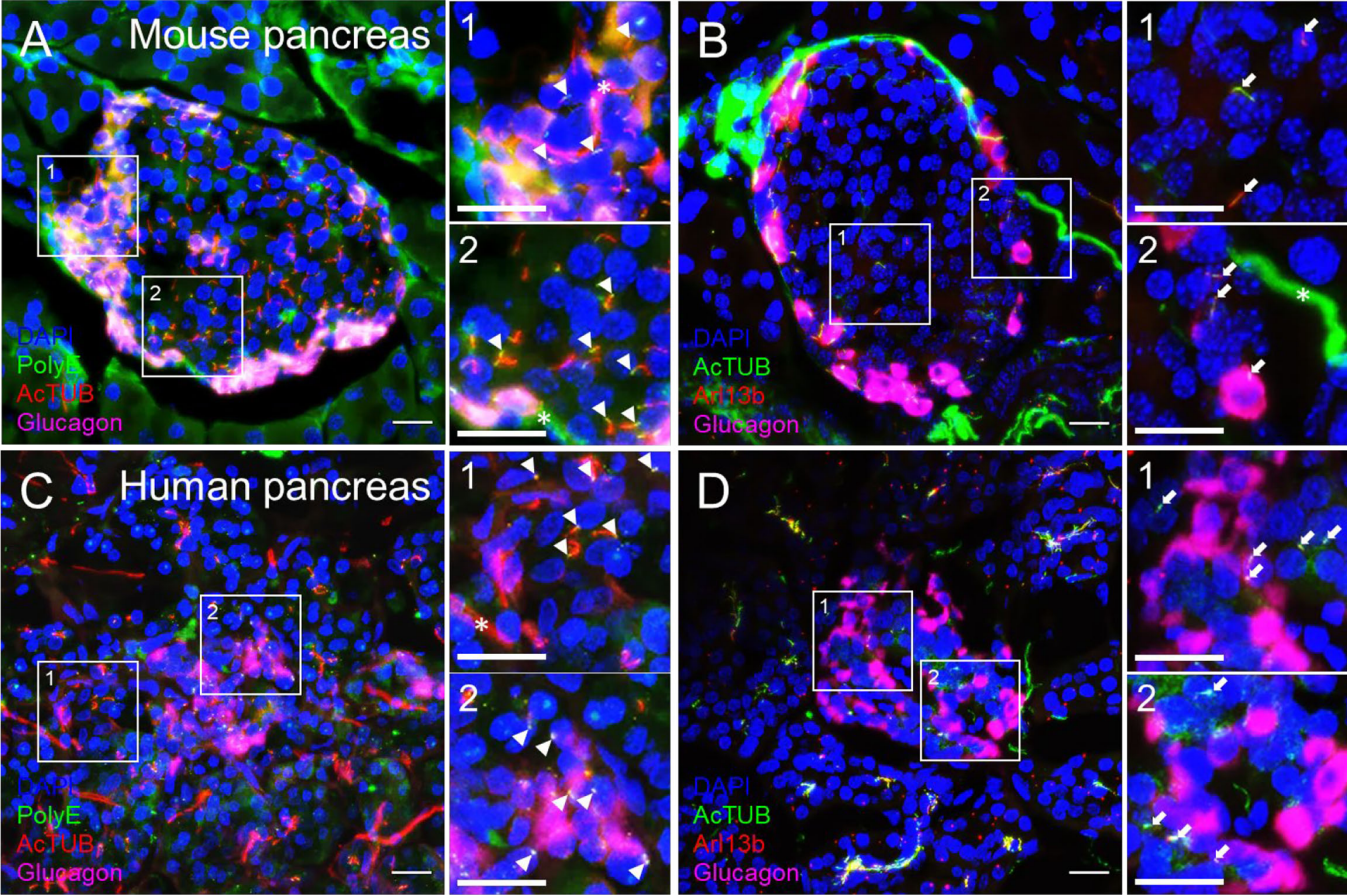
Comparison of cilia staining in mouse and human pancreas sections observed by confocal microscopy. Maximal projection of confocal images from mouse **(A, B)** and human **(C, D)** pancreata thin sections (10 µm) labeled with polyglutamylated tubulin (PolyE, green in **A, C**), acetylated α-tubulin (AcTUB, red in **A, C**, green in **B, D**), glucagon (magenta), and Arl13b (red in **B, D**). Nuclei, DAPI (blue). Magnified insets (1, 2) are located next to panels **(A-D)**. White arrowheads, proximal segment of cilia, White arrows, cilia axonemes. Asterisks, nerves. Scale bars, 20 µm.

**Figure 4:**
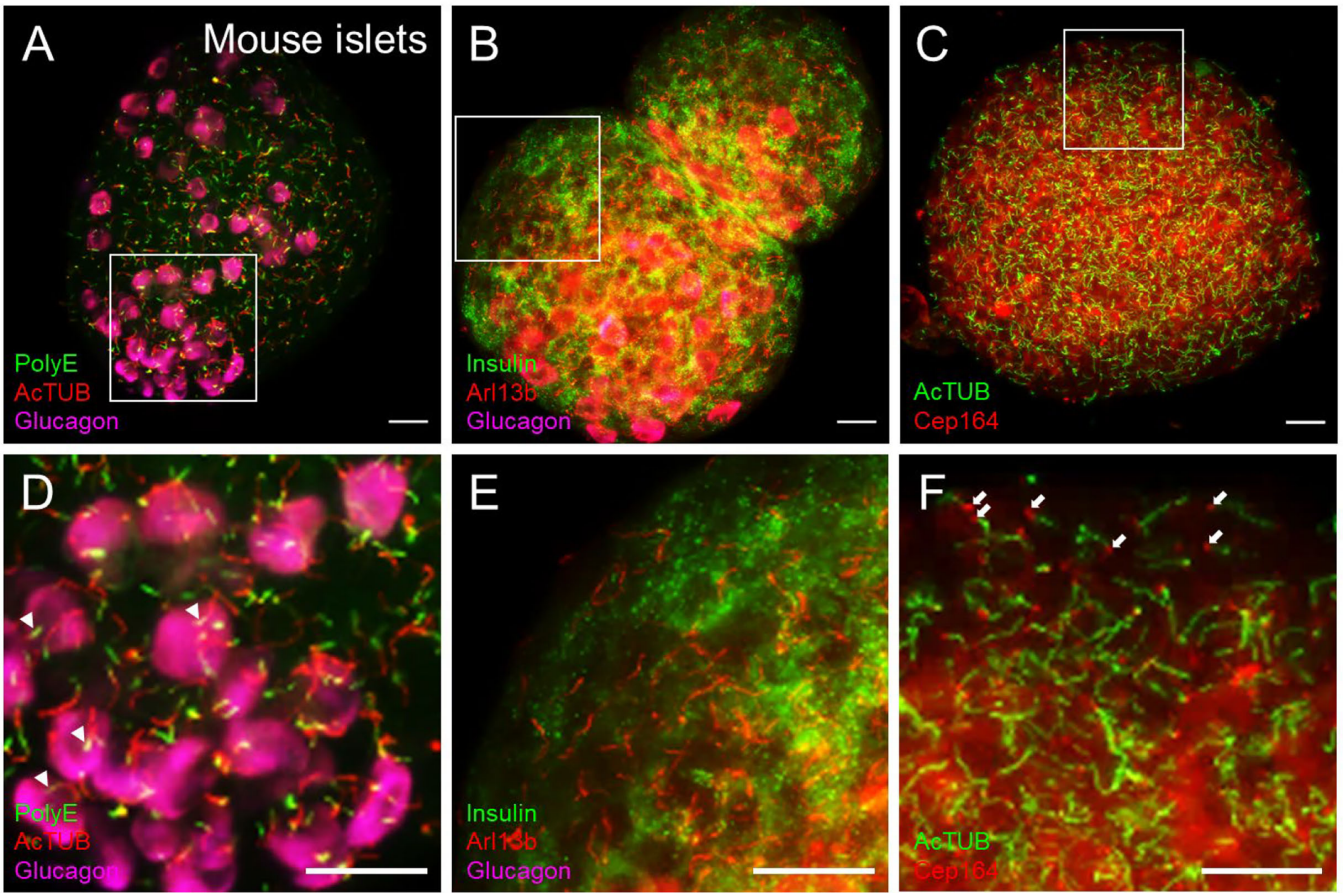
Cilia and centriole protein markers produce distinct staining patterns in islets. **(A)** Dual-labeled cilia in intact mouse islets showing polyglutamylated tubulin (PolyE, green) and acetylated α-tubulin (AcTUB, red) in a maximal projection. AcTUB and PolyE label different parts of the axoneme, which is better visualized in the magnified panel in **(D)** Alpha cells are labeled by glucagon (magenta). **(B)** The small GTPase Arl13b labels the cilia axoneme but can also have non-ciliary signal throughout the islet. A relatively “clean” region is shown in magnified panel **(E)** Beta cells are labeled by insulin (green), and alpha cells labeled by glucagon (magenta). **(C)** Cilia and basal body co-labeling using axonemal marker acetylated α-tubulin (AcTUB, green) and centriole marker Cep164 (red), showing differential localizations of the two proteins. Cep164 antibody also produces cytosolic and nuclear staining that is less focally intense than ciliary signals and may be reduced with antigen retrieval methods. **(D–F)** Magnified insets from **(A-C)**, respectively. White arrowheads in panel **(D)**, proximal segment of cilia. White arrows in panel **(F)**, basal bodies. All scale bars, 20 µm.

We recommend routine use of axial 3D stacks to imaging islets, as single-plane views rarely capture the entire length of even a single cilium, unless the cilium happens to align with the horizontal focal plane. In planar views, cilia can be missed or appear truncated or punctate. We typically analyze cilia in maximal intensity projections through tissue regions of interest (**Figure 3**). Between 0.1-1 μm thicknesses are recommended for z-stack intervals to facilitate smooth maximal projections, a consideration when generating downstream images. For pancreas sections, an additional challenge is to identify endocrine islet “islands” which are scattered among swaths of exocrine tissue (>98% of the pancreas). We typically co-stain with a hormonal marker such as glucagon or to look by DAPI for clusters of small, dense nuclei that typically correspond to islet regions (**Figure 4**). Cilia are more abundant in endocrine cells than in the exocrine pancreas except in pancreatic ducts which are abundantly ciliated and are morphologically distinct from islets, thus the location and density of ciliary staining can be a surrogate marker to identify islet regions in whole-pancreas sections.

### Antibody choices matter

Cilia labeling efficiency differs by antibody and by protein target, with axonemal antibodies generally detecting more abundant and intense cilia staining than basal body markers. The trade-off is that the strongest-staining antibodies tend to pick up non-ciliary signal, most notably acetylated alpha tubulin which is also present in neural fibers and can produce dense signals at the islet periphery (**Figure 3B**). In fact, acetylated a-tubulin has been used to study islet innervation (68, 69); it is also present in the cytosol in human islet cells, thus staining can appear non-specific unless combined with antigen retrieval methods. Arl13b reliably labels the axoneme (**Figures 3B, D**, **Figures 4B, E**) and are enriched in ciliary membranes (70), though the protein also has extra-ciliary functions (71) and thus may produce non-ciliary staining (**Figure 4B**). Basal bodies can be labeled *via* the centriole appendage protein Cep164 (red, white arrows, **Figures 4C, F**) which also has non-ciliary localization (red, **Figures 4C, F**). Of note, while many post-translational modifications of tubulin (72) provide useful targets for cilia antibodies, they each have non-uniform distribution along the axoneme, and therefore imaging expectations should be tailored. For example, in mouse islets, acetylated α-tubulin (AcTUB) labels the full length of the axoneme, while polyglutamylated tubulin (polyE, AdipoGen antibody GT335) detects only the proximal segment of cilia (**Figures 4A, D**).

Antibody performance varies depending on commercial vendor, lot number, host species, sample condition, and staining protocol. Typically, we use 1-2 fold higher cilia antibody concentration than manufacturer recommendations, e.g. 1:200 instead of 1:400, 1:500 instead of 1:1000, followed by more rigorous washing. Of the commercially available antibodies, we have curated a list of reliable cilia markers for either mouse or human islet staining, with specifications noted in **Table 1**. We recommend testing multiple antibodies, and each antibody should be individually. Acetylated α-tubulin, in particular, may benefit from a quick antigen retrieval step in 1% SDS for human islet specimens (data not shown). Glucagon and insulin antibodies can be used for co-labeling to show cilia distribution on alpha and beta cells, provided that the antibody titer be adjusted so as to not overpower the ciliary signal. Depending on experimental need and microscope laser capability, additional pancreatic structures may be labeled, including blood vessels, nerve fibers, and non-endocrine compartments. Islet vasculature remains intact and detectable for days *ex vivo* in culture and can be visualized with relative ease using staining antibodies such as CD31. The advent of multiplex imaging platforms such as CODEX (73) will facilitate detailed characterizations of the pancreatic and islet cilia network, allowing simultaneous visualization of many other structural markers *in situ* to reveal new information about ciliary functions in each tissue compartment.

**Table 1:** Recommended antibodies for islet cilia staining.

Antibodies	Manufacturer	Catalog#	Mouse	Human	Target
Mouse anti-acetylated α-tubulin	Sigma	T7451	✓	✓	Axoneme
Mouse anti-acetylated α-tubulin	Proteintech	66200-1-lg	✓	✓	Axoneme
Rabbit anti-acetylated α-tubulin	Cell Signaling Technology	5335	✓	✓	Axoneme
Rabbit anti-ARL13B	Proteintech	17711-1-AP	✓	✓	Ciliary membrane
Mouse anti-polyglutamylated tubulin (GT335)	AdipoGen Life Sciences	AG-208-0020 C100	✓	✓	Proximal axoneme, basal body

Validated commercial antibodies for immunostaining primary cilia in mouse and human islets. For all listed antibodies, 1:400 dilution is a good starting point; optimization is recommended. Acetylated α-tubulin antibodies generally produce non-ciliary staining in human islets and may require additional optimization.

### Fluorescence cilia reporter reveals live-cell cilia dynamics

**Figure 5:**
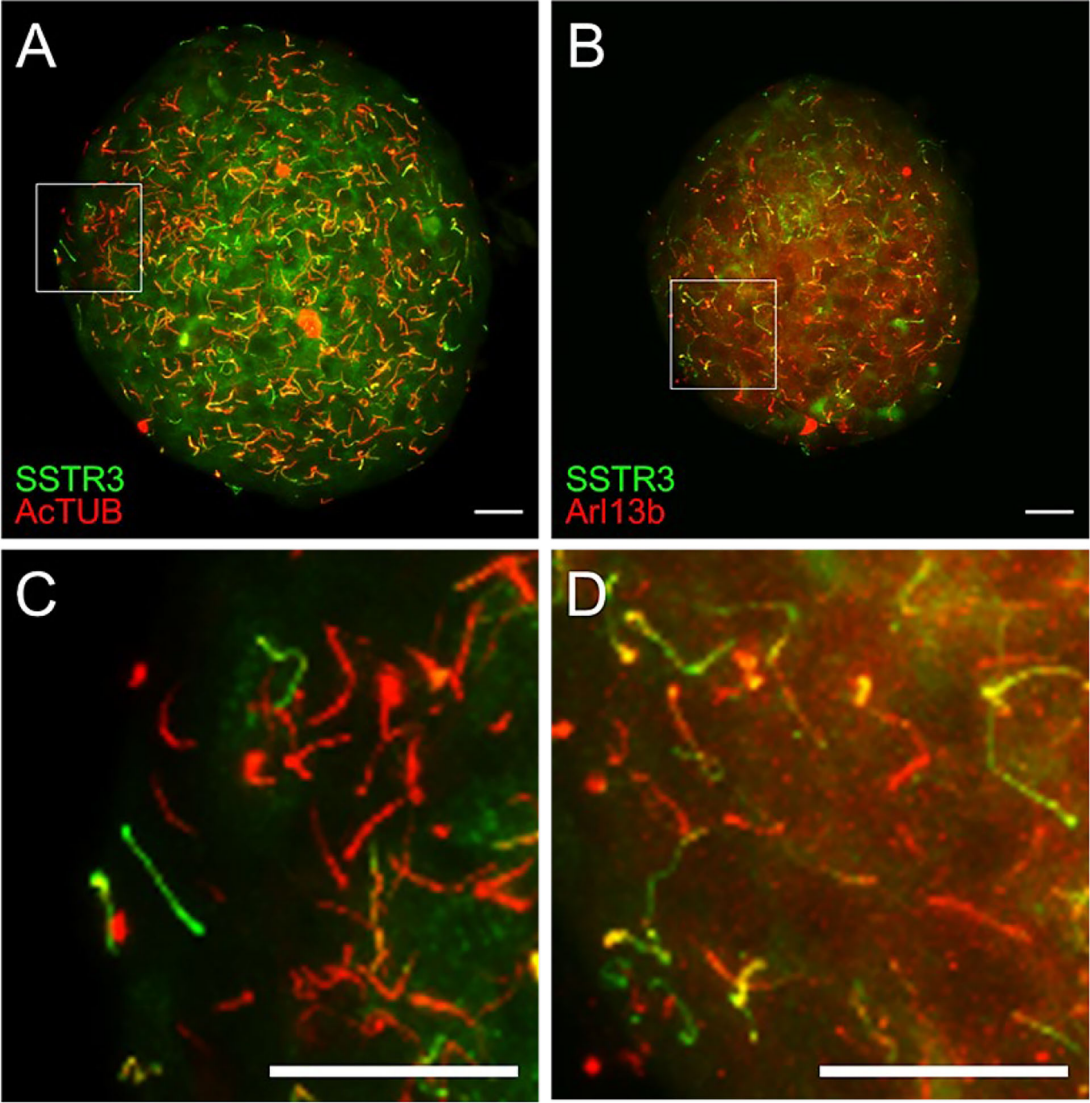
Validation of SSTR3-GFP cilia reporter by immunostaining. **(A, B)** Maximal confocal projections of whole islets from beta cell green cilia reporter SSTR3-GFP mice labeled with acetylated α-tubulin (AcTUB) or Arl13b in red. Green and yellow correspond to beta cell cilia; red represents non-beta cell cilia. **(C, D)** Magnified inset images from panels A and B, respectively. Scale bars, 20 µm.

Using the Ins1-Cre SSTR3-GFP^OFF^ reporter mouse, we recently characterized a wave-like axonemal motility in beta cell primary cilia, which is dependent on the presence of glucose, ATP, and dynein molecular motors (35). The ability of beta cell cilia to move and form physical connections likely contributes to their sensory roles in the islet niche and a communications device among adjacent cells and structures. Thus, beyond the static organization of the islet primary cilia network, the dynamic behavior of primary cilia is also informative for understanding their functions in intact islets. Time-lapse motion recordings of primary cilia in live islets and tissues requires stable and bright fluorescent labeling to make the cilia visible. Our beta cell-specific cilia reporter mice thus represent a useful model for studying ciliary motion in beta cells. Ciliary membrane SSTR3 expression in the transgenic mouse islet can be validated by immunostaining against known cilia markers such as Arl13b and AcTUB, with green signals corresponding to beta cell cilia expressing Cre-driven SSTR3-GFP (**Figure 5**). Alpha or delta cell-specific cilia reporter models can be generated using dedicated Cre lines using the same methodology, and the fluorescent marker can be ciliary proteins other than SSTR3, such as Arl13b or centrin (74, 75). The labeling of mature ciliary axonemes by fluorescent fusion proteins may also allow distinguishing of ciliated cells by FACS sorting based on reporter expression.

While primary cilia may lack vigorous or coordinated motion, they are nonetheless highly dynamic organelles that constantly elongate, shrink, remodel, and traffic molecules in and out, and in certain settings the whole axonemal structure has been observed to move to extracellular or intracellular forces (28, 33, 76, 77). **Video 1** shows a collage of ciliary motion on the islet periphery in low (1 mM) versus high (11 mM) glucose, with ciliary motility increasing in response to higher glucose. Islet cilia are relatively slow-moving compared to classic motile cilia, with irregular wave periods on the order of 20-30 seconds and variable sweep trajectory between beat strokes (35). We typically record multiple continuous waveform cycles per cilium to facilitate statistical analysis, keeping the sampling frequency or temporal resolution of live cilia imaging no less than 1 frame per 2 seconds to allow smooth capture of the ciliary path. These temporal parameters are slow enough to allow a limited z-stack to visualize multiple cell layers in a 3D islet volume and to track cilia that move in and out of the focal plane. This typically translates to 60-120 cycles over a continuous 10-minute imaging duration per field of view, 10-15 vertical slices (if acquiring z-stack images) at 1 μm interval, and overall 512x512 pixel size with 0.26-0.44 µm/pixel imaging scale. Waveform analysis is performed at the level of individual cilia, and images are analyzed in single channel over a time course, analysis method described in a dedicated section below. Quantitative assessment of waveform parameters typically shows variability in waveform among different cilia in the same islet. The amplitude or “sweep” of cilia movement is more pronounced on the islet periphery, a phenomenon possibly related to the artificial *ex vivo* environment. In comparison, we have observed in pilot intravital imaging studies using intact pancreas that cilia movement at the islet boundary is as physically restricted as in the islet core, though both observable and quantifiable (data not shown). Speculatively, the heterogeneity in beta cell cilia movements may be another phenotype in the functional beta cell diversity.

### Live-cell labeling of primary cilia by cADDis and SiR-tubulin

**Figure 6:**
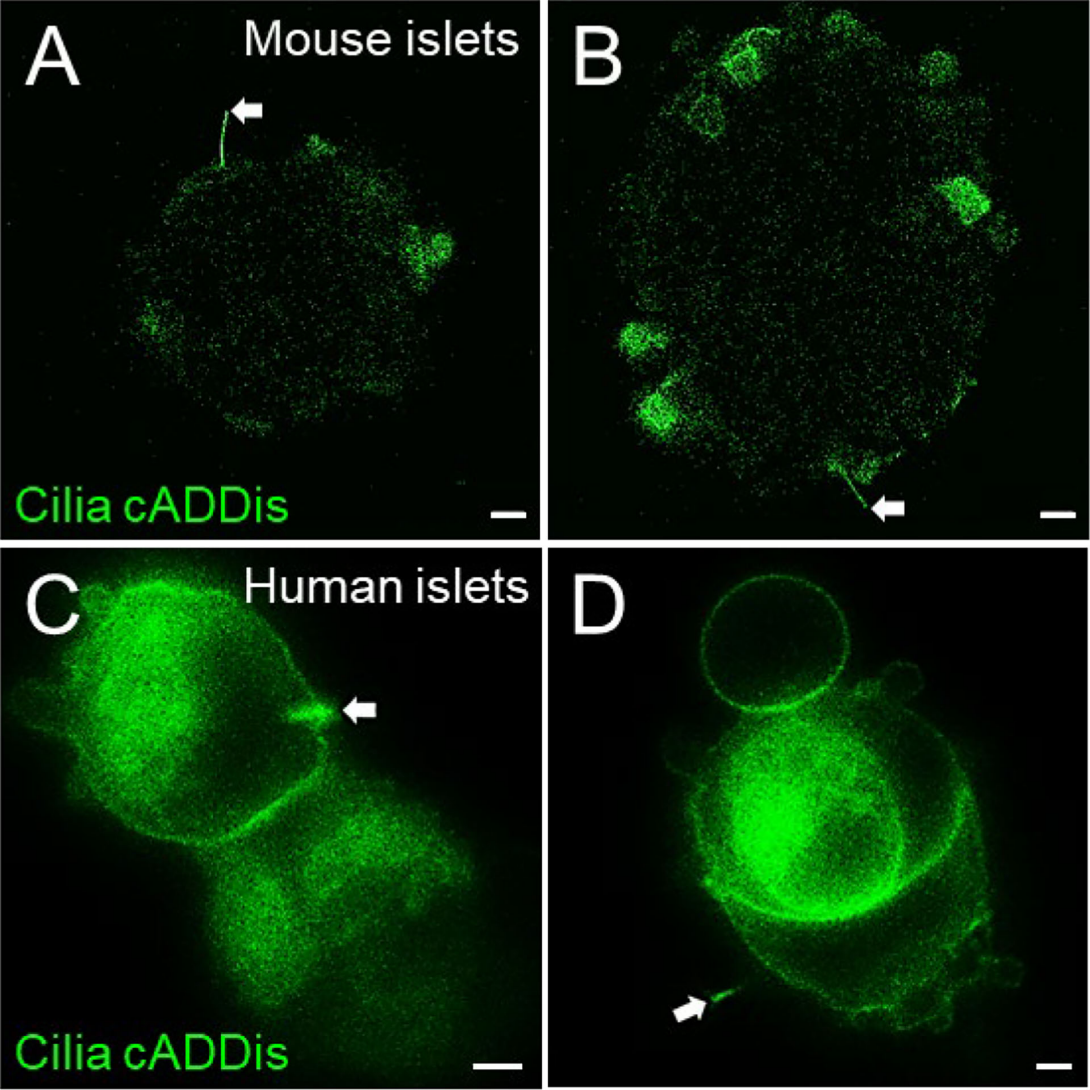
Green cADDis labeling of live islet cell cilia. Snapshot images from live-cell recordings of intact mouse islets **(A, B)** and partly dissociated human islets **(C, D)** 24 hours after transduction with baculovirus containing cilia-targeted green cADDis sensors. White arrows indicate primary cilia. Scale bars, 10 µm **(A, B)**, 2 µm **(C, D)**.

Live-cell sensors for molecules that traffic in and out of the cilium, such as cAMP, calcium, and tubulin make useful cilia imaging markers. The BacMam vector that carries the cADDis can infect both mouse and human islets, albeit with low efficiency and targeting mostly peripheral cells (**Figure 6**, white arrows). Transduction efficiency may be improved by the use of sodium butyrate, a histone deacetylase (HDAC) inhibitor for more stable BacMam expression, and by partially dispersing intact islets with Accutase (Innovative Cell Technologies AT-104) before baculovirus infection. Practical considerations in using the cADDis sensor include balancing transduction efficiency with cytotoxicity when using high viral titers, potential damage from the dissociation process, and the need for experimental optimization. There can also be non-ciliary expression of the cADDis sensor in the plasma membrane and cytosol. Nonetheless, the green cilia cADDis can reliably label a small percentage of cilia in native islet cells.

**Figure 7:**
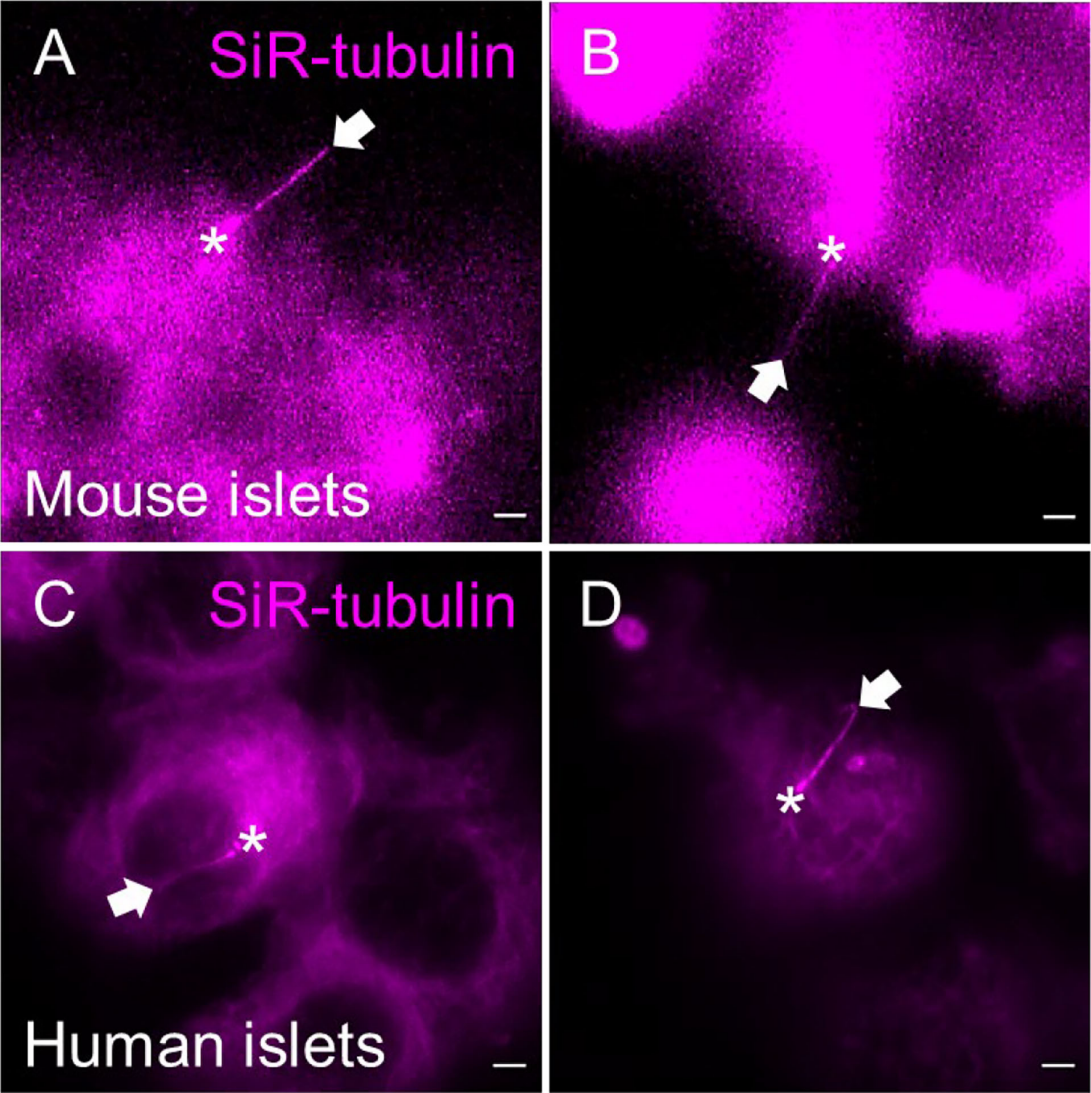
SiR-tubulin labeling of islet cilia for live-cell imaging. The far-red microtubule probe SiR-tubulin (magenta) labels primary cilia in partially dissociated and reaggregated mouse **(A, B)** and human **(C, D)** islets. Still images represent maximal intensity projections of confocal z-stacks. White arrows, cilia. Asterisks, centrioles. Scale bars, 2 µm.

As with cADDis, SiR-tubulin produces bright and cilia-selective staining, labeling primary cilia and centrioles with acceptable efficiency that they could be distinguished from the level of background signal in the cytoplasm (**Figure 7**). Verapamil, an efflux-pump inhibitor, may be added per manufacturer recommendations to increase SiR-tubulin labeling efficiency. Of note, like cADDis, SiR-tubulin does not penetrate more than a few cell layers in intact islets, so it is helpful to partially dissociate islets prior to labeling. Newer spirochrome-based SPY probes from the same parent company promise even greater signal-to-noise ratio with less cytotoxicity than SiR probes, but which we have not yet tested in islets.

### Cilia waveform analysis

**Figure 8:**
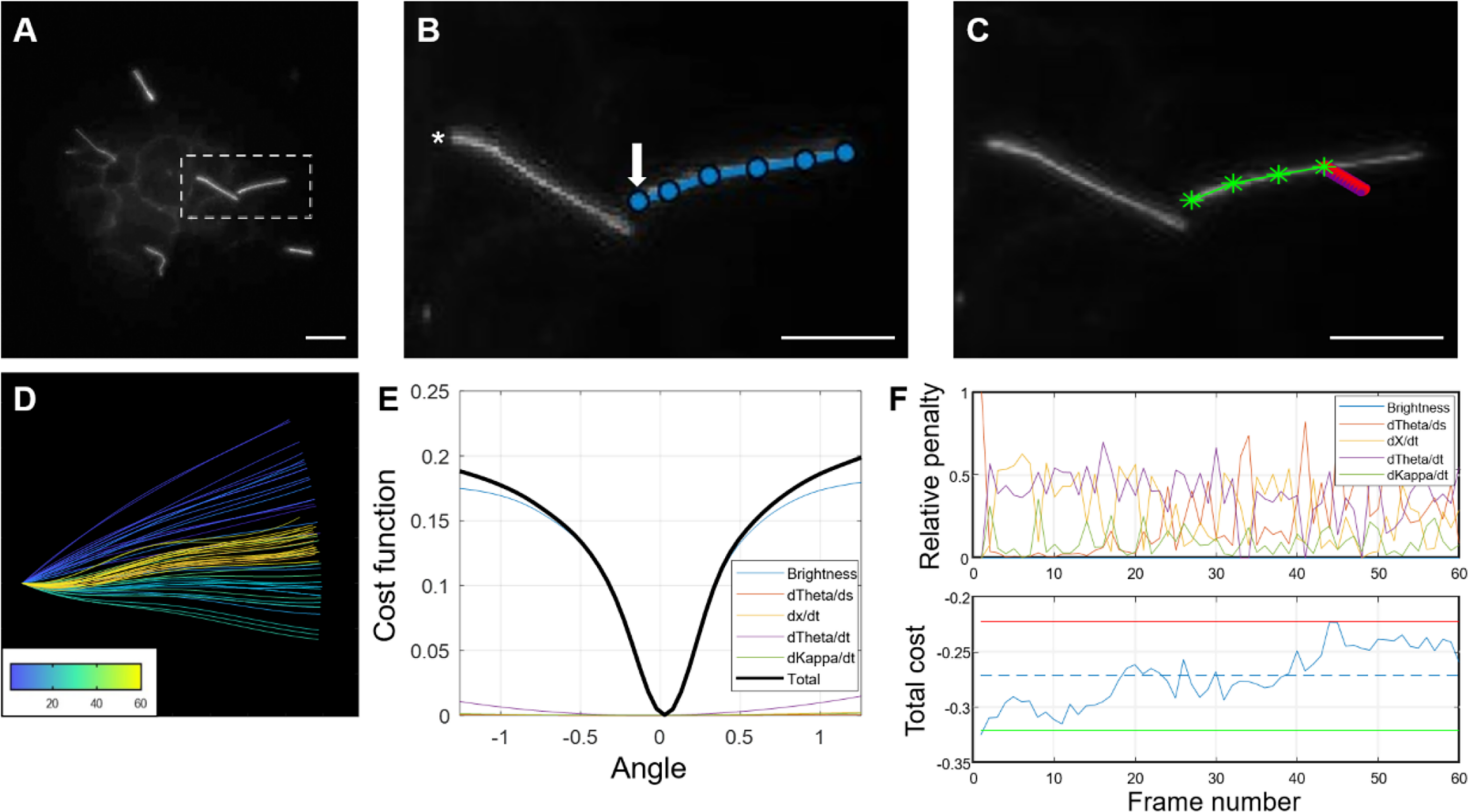
Step-by-step demonstration of MATLAB cilia waveform autotrace and quantification. **(A)** Grayscale image of a user-defined frame from time-lapse video of ciliary motion, allowing manual selection of the cilium of interest and identification of a stationary cilia base as reference for tracing. **(B)** Magnified inset showing user-defined traces (blue line) from the ciliary base (arrow) to tip and another ciliary base (asterisk) not quantified in this example. Scale bars, 10 µm. **(C)** Automated cilia tracing by the program. Green line represents completed waveform traces by searching for regional maxima, and red represents a rotating rectangular array of points spanning the width and length of the first cilium segment **(D)** Color-coded waveforms of the cilium in **(C)** corresponding to a temporal heatmap. **(E)** Dynamic cost function of waveform tracing. The angle chosen is the one that minimizes a linear combination of the (negative) weighted pixel intensity under the probe array as well as penalty terms used to improve robustness in cases of noise, poor contrast, and focus. **(F)** Example of the relative penalties and total cost for a 60-frame trace. For the total cost plot, the dashed line represents the mean cost, the upper (red) and lower (green) lines represent two standard deviations above and below the mean, respectively.

**Figure 9:**
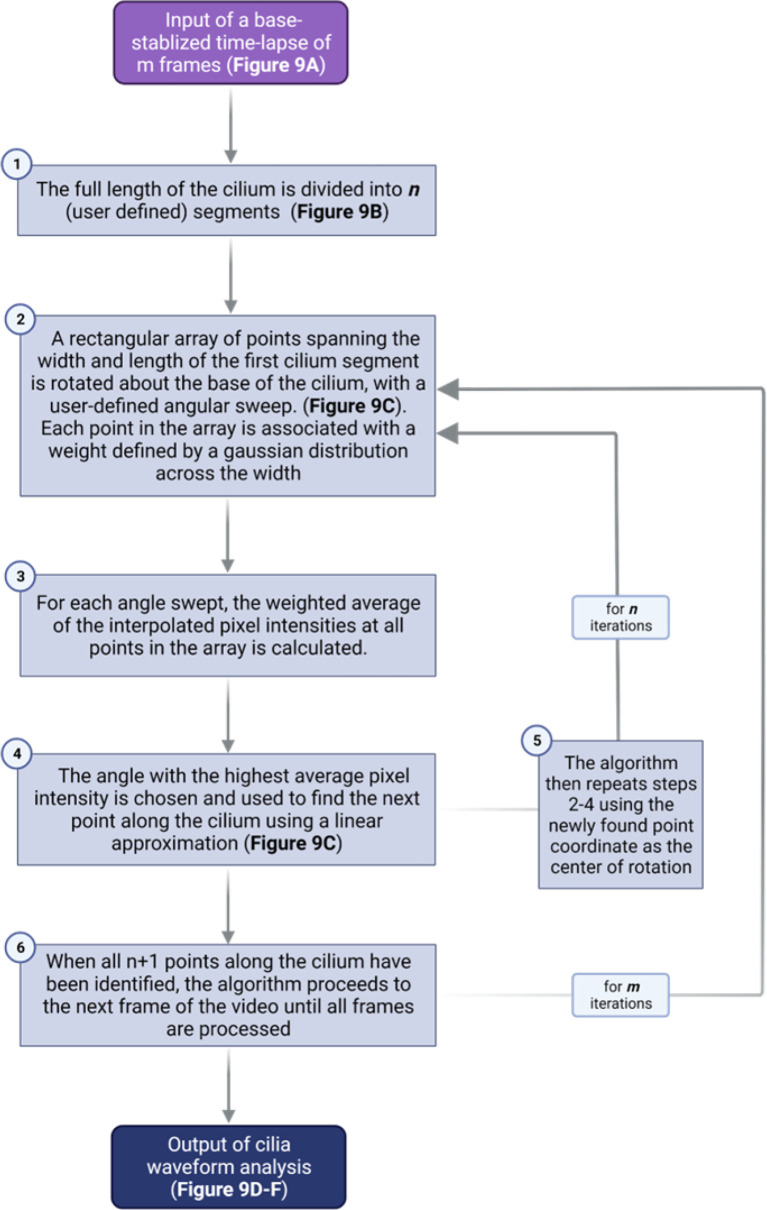
Flowchart outlining the algorithm for cilia waveform analysis.

Quantitative waveform analysis can be performed on genetically- or sensor-labeled cilia in whole islets. Cilia waveform traces were obtained using a custom-built program written in MATLAB (The MathWorks Inc., Natick, MA) (35, 78). Using the first frame of a dynamic primary cilia *in-vivo* time lapse video, the user manually identifies a cilium base, then traces along the cilium to obtain length and set the initial angle for automatic tracing (**Figure 8**). The program then starts automatically tracing the cilia in successive frames by iterating through a streamlined algorithm (**Figure 9**). To improve the robustness and reliability of the program, terms are applied to penalize curvature, translational velocity, rotational velocity, and rate of change of curvature. These penalty terms help the algorithm ‘stay on track’ when issues with image focus, contrast, noise, or other objects in the field make the angle of the cilium unclear at a given segment. Quantities of interest such as amplitude, beating frequency, cilia length in μm, period, periodicity, and maximal curvature are calculated by post-processing of the raw angle data obtained above. Cilia beating amplitude is reflected by calculating the standard deviation of theta, the angle between the tangent vector of the cilium and the x-axis, of all points within the middle 80% of the length of the cilium, excluding the base and the tip. As islets can move during live-cell imaging due to treatments or changes in osmotic pressure, it is necessary to remove translation of the ciliary base as this motion is not accounted for in the software described above. This was accomplished using a previously developed stabilization method based on the fast Fourier transform (78). We have modified the software to allow selection of a range of frames within a time-lapse video, which allows for comparative analysis of cilia motility under different treatment conditions without two separate imaging inputs. This feature is especially useful when analyzing the glucose-induced cilia motility, which requires a changing concentration gradient of glucose.

### Looking forward: Superresolution and ultrastructural imaging

Recent advances in superresolution (SR) microscopy techniques herald exciting opportunities for the study of islet cilia structure. In immortalized cell lines and non-islet tissues, the use of expansion microscopy (ExM) using polyelectrolyte gels to physically expand the sample up to ~4-fold in the linear dimension has allowed greater spatial resolution, generating ultrastructural details rivaling conventional electron microscopy (79–81). These methods have not yet been applied to the study of islet primary cilia. The successful adaptation of ExM to islet cilia will largely depend on the fluorescent labeling strategy and compatibility of antibodies or fluorescent proteins with the proteolytic treatment and subsequent expansion. Expanded samples can be used in combination with structured illumination microscopy (SIM) or Zeiss Airyscan to provide an additional ~2-fold increase in resolution as well as greater signal-to-noise ratio, thereby enabling subdiffraction imaging of cilia and centrioles.

In addition to superresolution imaging modalities, the use of correlative light and electron microscopy (CLEM) is another strategy to obtain ultrastructural information on targeted ciliary regions (82). For pancreatic islets, ultrastructural studies of primary cilia have been limited by difficulties identifying these relatively rare objects in native tissue blocks. CLEM in theory bypasses this limit as ciliary localizations can be precisely resolved by protein staining and fluorescence microscopy prior to ultrastructural imaging by electron microscopy (EM) or electron tomography (ET). In practice, cilia-staining fluorescent antibodies rarely survive the glutaraldehyde fixation required for EM or ET sample prep, therefore, the use of chemical tags or genetic reporters may be a more compatible approach than antibody staining for cilia identification in CLEM. Multi-color CLEM is also theoretically possible to separately mark beta and non-beta cell cilia to study heterotypic cilia interactions. We expect that these future ultrastructural studies of islet primary cilia will identify physical features that explain their motility and roles in islet cell communication.

## Summary

Primary cilia are an important sensory organelle and a dynamic structure to study by fluorescence microscopy. Emerging data suggest that primary cilia play pleiotropic roles in pancreatic islets including cell-cell communication, paracrine signaling, glucose-regulated calcium dynamics, and many other cellular processes that can benefit from direct visualization by imaging. The techniques summarized here represent our experiences thus-far and can be used as a field guide for those studying cilia morphology and behavior in the context of islet cell function. For both fixed- and live-cell imaging in both mouse and human islet specimens, the use of 3D imaging is important for visualizing cilia throughout the entire islet volume. Other considerations include imaging depth vs breadth, whether the experiment requires subcellular detail or panoramic whole-organ scale, and for live-cell imaging, the choice to prioritize spatial vs temporal resolution. The choice of imaging modality determines what you see; conventional static imaging using antibody-based staining provides a reliable approach to visualize primary cilia in fixed tissues (8), while time-lapse microscopy captures ciliary waveform, physical coupling, and other dynamic behavior (35, 83). Thus, it is useful to consider the strengths and limitations of each imaging approach and to employ them in a complementary manner.

## Data availability statement

The datasets presented in this study can be found in online repositories. The names of the repository/repositories and accession number(s) can be found below: The MATLAB program code used for cilia waveform autotrace is deposited *via* Zenodo at https://doi.org/10.5281/zenodo.6687921.

## Ethics statement

Ethical review and approval was not required for the study on human participants in accordance with the local legislation and institutional requirements. Written informed consent for participation was not required for this study in accordance with the national legislation and the institutional requirements. The animal study was reviewed and approved by The Washington University School of Medicine Institutional Animal Care and Use Committee.

## Author contributions

ZL and JC contributed imaging data. LW wrote the cilia autotrace code. All authors wrote and approved the manuscript.

## Funding

This study was funded by NIH grant DK127748 and DK115795 to JWH. Human islets were acquired from the NIDDK-funded Integrated Islet Distribution Program (IIDP) (RRID : SCR_014387) at City of Hope, NIH grant #2UC4DK098085 and the JDRF-funded IIDP Islet Award Initiative. Light microscopy was performed at the Washington University Center for Cellular Imaging (WUCCI), supported by Washington University School of Medicine, The Children’s Discovery Institute of Washington University and St. Louis Children’s Hospital (CDI-CORE-2015-505 and CDI-CORE-2019-813) and the Foundation for Barnes-Jewish Hospital (3770 and 4642).

## Acknowledgments

We are grateful to the donors and families that made human pancreatic islets available to our research. SSTR3-GFP parental mice were a kind gift from Brad Yoder Lab at UAB. We thank members of the Hughes Lab for their discussion of the manuscript and our reviewers for critical feedback. Graphic illustrations were created with BioRender.com.

## Conflict of interest

The authors declare that the research was conducted in the absence of any commercial or financial relationships that could be construed as a potential conflict of interest.

## Publisher’s note

All claims expressed in this article are solely those of the authors and do not necessarily represent those of their affiliated organizations, or those of the publisher, the editors and the reviewers. Any product that may be evaluated in this article, or claim that may be made by its manufacturer, is not guaranteed or endorsed by the publisher.

## Supplementary material

Supplementary Video 1 High-speed video microscopy showing beta cell cilia motility in 1 vs. 11 mM glucose. Ins1-Cre SSTR3-GFP reporter mouse islets exhibit spontaneous beta cell cilia movement under differential glucose concentrations. Islets are imaged in KRBH imaging buffer containing 1mM (low glucose) and 11 mM (high glucose) glucose and collaged into one video image. For each glucose concentration, time-lapse z-stack video images are captured with a frame time of 624.24 ms for a total duration of 412 seconds. Maximum intensity projection was performed to consolidate all cilia in the z-axis. Time stamp in the upper right corner indicates total elapsed time mapped to a 10-second video. Scale bar, 10 µm.

SUPPLEMENTARY DATA SHEET 1 Autotrace.m file published in PDF with sample traces.
