# Supplementary material for: Fluorescence imaging of beta cell primary cilia
Source: Front Endocrinol (Lausanne). 2022 Sep 23;13:1004136. doi: 10.3389/fendo.2022.1004136 (PMC9540379; doi:10.3389/fendo.2022.1004136)
Supplement: SUPPLEMENTARY DATA SHEET 1 — Autotrace.m file published in PDF with sample traces. [file DataSheet_1.pdf]

```
function [Out, FileInfo] = autotrace_fun3(FileInfo)

% autotrace cilia
% Louis Woodhams 09/2020

% This is the continuous version. Sweeps a 'gaussian rod' rotating about
% current point to find best angle to next point.
% Still a work in progress, but it does work...

% BE AWARE: points and angles are in image coordinates, not cartesian
% coordinate, you may need to reverse angle and y-values from outputs

% The 100 x we are using right now is 0.194 um/pixel,
% and the 63 x is 0.306 um/pixel (Mathieu’s data is 0.169 um/pixel).

%****************** USER PARAMETERS *************************************
umpp = 0.26;   % um/pixel spatial resolution of image
fps = 0.146;     % frames/sec,


n = 5;     % number of segments along length
nT = 50;    % number of theta values to try
dd = 3;     % rod width in pixels
sd = 0.8;   % standard deviation of gaussian dist (normalized)

sweepWidthRatio = 2; % if this is too low, angles may be out of range
a1 = -1;     % weight of correlation value
a2 = .05;   % penalty for curvature along length (dTheta/ds)
a3 = .1;    % penalty for change in position from last frame (dX/dt)
a4 = .001;  % penalty for change in angle from last frame(dTheta/dt)
a5 = .001;   % penalty for change in curvature from last frame(dKappa/dt)
interpType = 'cubic';

if nargin == 0
    [file,path] = uigetfile({'*.avi;*.mp4;*.mpg;*.mpeg','Videos';...
        '*.*','All Files'}, 'Select a video');
    fileName = fullfile(path,file);
else
    fileName = FileInfo.fileName;
end

showTrace = 1; % show trace path graphically?
showSweep = 0; % show sweep path graphically? (requires trace)
showStats = 1;
showPlot = 1;
showTrace2 = 0;
step = 0; % step through points
ndp = 5; % number of points in disk diameter (keep odd)
makeGif = 0;
gifName = 'c:\gifs\autotrace.gif';
%***************************************************************************

dt = 1/fps;     % time step
```

```
% could improve/add:
% - vectorize sweep ()
% - better tool for viewing paths after the fact
% - way to easily fix broken traces (after or while running?)
% - integrate into existing code
% - optimize parameters?
% - use initial trace(s)(by hand) to calibrate penalty terms?
%       - first trace is now without history based penalty terms
% - correlate with line or curve sweep? [that is what this version does]
% - use predictions based on history to help guide curve (~multistep)
% -

v = VideoReader(fileName);
numFrames = v.NumFrames;

% get start and end frames
if nargin == 0
    prompt = {'first frame','last frame'};
    dlgtitle = 'Input';
    dims = [1 20];
    definput = {'1',num2str(numFrames)};
    answer = inputdlg(prompt,dlgtitle,dims,definput);
    firstFrame = str2double(answer{1});
    lastFrame  = str2double(answer{2});
    numFrames = lastFrame - firstFrame +1;
else
    firstFrame = FileInfo.firstFrame;
    lastFrame  = FileInfo.lastFrame;
end
nFrame = read(v,firstFrame);
nFrame = im2double(nFrame);
if size(nFrame,3)==3, nFrame = rgb2gray(nFrame); end

% this is for visual interoplation of the image. It is just showing what
% the interpolation looks like (as a way of seeing if it creates any
% strange artifaces, etc.)
interpFactor = 4;
[rows,cols] = size(nFrame);
[Xint, Yint] = ndgrid(1:1/interpFactor:rows,1:1/interpFactor:cols);

costArray = zeros(numFrames,5); % compare cost terms
totalCost = zeros(numFrames,1); % see if there are any high frames here


thetaSweepVec = linspace(-pi,pi,nT)*sweepWidthRatio/n; % angles to sweep
pointArray = zeros(n+1,2,numFrames);
thetaArray = zeros(n+1, numFrames);
c3 = 0; c4 = 0; c5 = 0;
```

get an initial line trace from base to tip then double click

this gets us length, initial position, and initial angle

```
if nargin == 0
    imshow(nFrame)

    roi = drawpolygon('FaceAlpha',0);
    % roi = drawassisted;
    % roi.Closed = 0;

    % find arclength
    line1 = roi.Position;
    close
    delete(roi)
else
    line1 = FileInfo.line1;
end

disp = line1(2:end,:)-line1(1:end-1,:);
    dist = sqrt(disp(:,1).^2 + disp(:,2).^2);
    arcLenPix = sum(dist);
    r = arcLenPix/n;   % individual segment length
    r_um = r*umpp; r_m = 1e-6*r_um; % in umints of um
    rs = ceil(r); % need to fix this? rounding segment length up for sweeper


startTime = tic;

d = gaussRod(ndp,rs,sd); % this is the 'rod' we will sweep across the cilium
dSum = sum(d(:));
dVec = linspace(-dd/2,dd/2,ndp); % points around zero to evaluate for corr
Y0 = repmat(dVec',[1, rs+1]);
X0 = repmat(0:rs,[ndp, 1]);
XY0 = [X0(:), Y0(:)]';

ind = 1;
for iFrame = firstFrame:lastFrame

    nFrame = read(v,iFrame);
    nFrame = im2double(nFrame);
    if size(nFrame,3)==3, nFrame = rgb2gray(nFrame); end
    I = griddedInterpolant(nFrame,interpType,'none');
    nFrameInt = I(Xint, Yint);
```

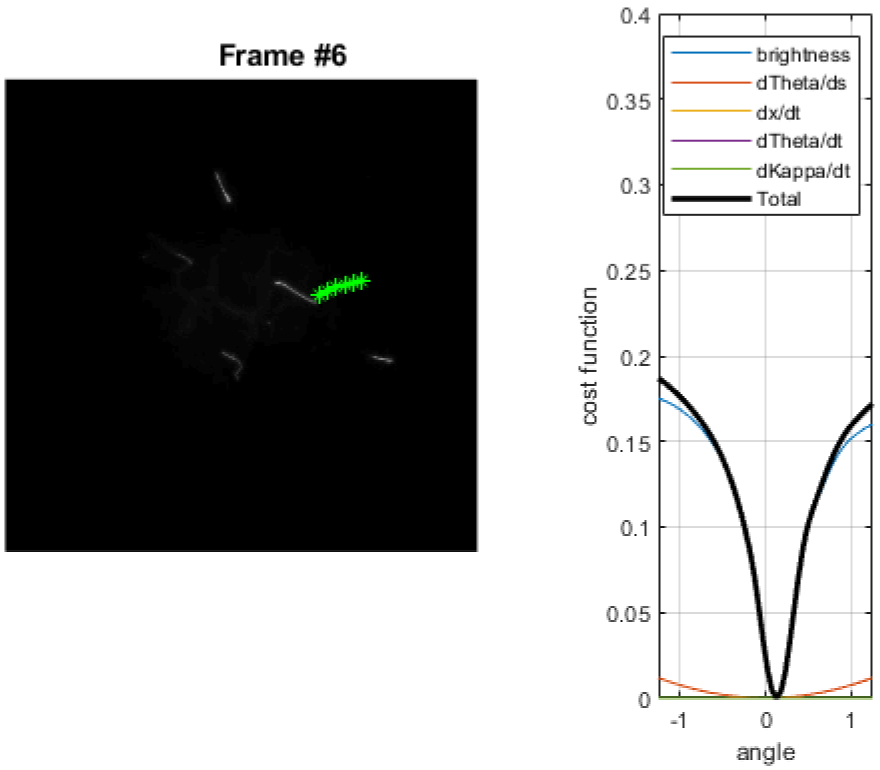

initial pos and angle

```
x = NaN(n+1,2);
x(1,:) = line1(1,:);
theta = zeros(n+1,1);
if ind == 1
    theta(1) = atan2(disp(1,2), disp(1,1));
    if ~showTrace, w1 = waitbar(1,'Finding paths'); end
else
    theta(1) = thetaBaseLast;
end

if showTrace
    figure(1)
    subplot(1,3,[1 2])
    h = imshow(nFrameInt);
    h.XData = [1 cols];
    h.YData = [1 rows];
    axis auto
    if makeGif && ind == 1
        gif(gifName,'DelayTime',1/30)
    end
    hold on
    if showSweep
        % h1 = plot(x(:,1),x(:,2),'r-','LineWidth',3);
```

```
        % h1 = plot(XY0(1,:),XY0(2,:),'r.','MarkerSize',3);
        pColors = [d(:),zeros(size(d(:))),1-d(:)];
        h1 = scatter(XY0(1,:),XY0(2,:),d(:)'*10,pColors,'filled');
    end
    title(['Frame #' num2str(iFrame)])
    h2 = plot(x(:,1),x(:,2),'g*-');
    hold off
    subplot(1,3,3)
    h3 = plot(thetaSweepVec, zeros(length(thetaSweepVec),5));
    hold on
    h4 = plot(thetaSweepVec, zeros(length(thetaSweepVec),1),'k');
    hold off
    h4.LineWidth = 2;
    legend('brightness','dTheta/ds','dx/dt','dTheta/dt',...
        'dKappa/dt','Total')
    ylim([0 0.4])
    xlim([thetaSweepVec(1) thetaSweepVec(end)])
    grid
    xlabel('angle')
    ylabel('cost function')
else
    waitbar(ind/numFrames,w1)
end


for iStep = 1:n
    %initialize cost and position arrays
    c = zeros(nT,5);
    xVec = zeros(nT,2);

    for iTheta = 1:length(thetaSweepVec)
        nTheta = (thetaSweepVec(iTheta) + theta(iStep));
        iX = x(iStep,:)+[r*cos(nTheta), r*sin(nTheta)];

        % create rotation matrix with current trial angle nTheta
        ROTMAT = [cos(nTheta), -sin(nTheta);
            sin(nTheta),  cos(nTheta)];
        % rotate and translate 'rod' of evaluation points
        XYRot = ROTMAT*XY0+x(iStep,:)';
        % evaluate intensities at these coordinates
        blockVal = I(XYRot(2,:),XYRot(1,:));
        % take weighted average using weights in 'd'
        if showSweep && showTrace
            %                 h1.XData = [x(iStep,1) iX(1)];
            %                 h1.YData = [x(iStep,2) iX(2)];
            h1.XData = XYRot(1,:);
            h1.YData = XYRot(2,:);
            drawnow
            pause(0.01)
            % gif
        end
        c1 = a1*dot(d(:),blockVal(:))/dSum; % weighted average pixel vals
        if isnan(c1) % assign penalty for going out of image bounds
            c1 = a1*10;
```

```
        end
        % curvature cost ~(dTheta/ds)^2
        c2 = a2*(thetaSweepVec(iTheta)/r_um)^2;
        if ind > 1
            % c3 is cost due to (dX/dt)^2, dXdt in um/s
            dXdt = norm(iX - pointArray(iStep+1,:,ind-1))*umpp/dt;
            c3 = a3*dXdt^2*(1-iStep/(n+1))/1e6; % diminishes along length
            % c4 is cost due to dTheta/dt
            dThetadt = (nTheta - thetaArray(iStep+1,ind-1))/dt;
            c4 = a4*dThetadt^2/1e6;
            % c4 = c4*(1-iStep/(n+1))/arcLen; % diminishes along length
            % c5 is cost due to d(dTheta/ds)/dt
            c5 = a5*(thetaSweepVec(iTheta)-...
                (thetaArray(iStep+1,ind-1)-...
                thetaArray(iStep,ind-1)))^2/r_um^2/dt^2/1e6 ;
        end

        c(iTheta,:) = [c1, c2, c3, c4, c5];
        xVec(iTheta,:) = iX;
    end

    [minVal, minInd] = min(sum(c,2));
    totalCost(ind) = totalCost(ind) + minVal/n;
    c = c - min(c,[],1);
    costArray(ind,:) = costArray(ind,:) + c(minInd,:)/n;
    theta(iStep+1) = theta(iStep)+thetaSweepVec(minInd);
    x(iStep+1,:) = xVec(minInd,:);
    if iStep == 1
        thetaBaseLast = theta(iStep)+thetaSweepVec(minInd);
    end

    if showTrace
        h2.XData = x(:,1);
        h2.YData = x(:,2);
        c = c - min(c,[],1);
        h3(1).YData = c(:,1);
        h3(2).YData = c(:,2);
        h3(3).YData = c(:,3);
        h3(4).YData = c(:,4);
        h3(5).YData = c(:,5);
        h4.YData = sum(c,2);
        drawnow
        if step, pause, end
    end
end
pointArray(:,:,ind) = x;
thetaArray(:,ind) = theta(:);
if makeGif, figure(1), gif, end
ind = ind + 1;
```

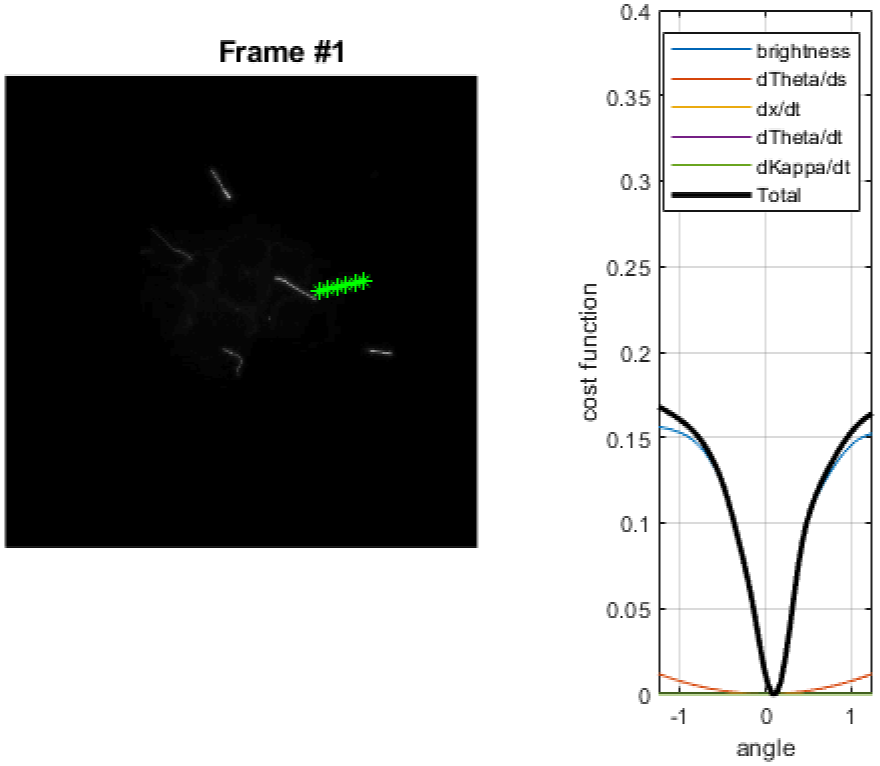
Frame #1
brightness
dTheta/ds
dx/dt
dTheta/dt
dKappa/dt
Total
cost function
angle

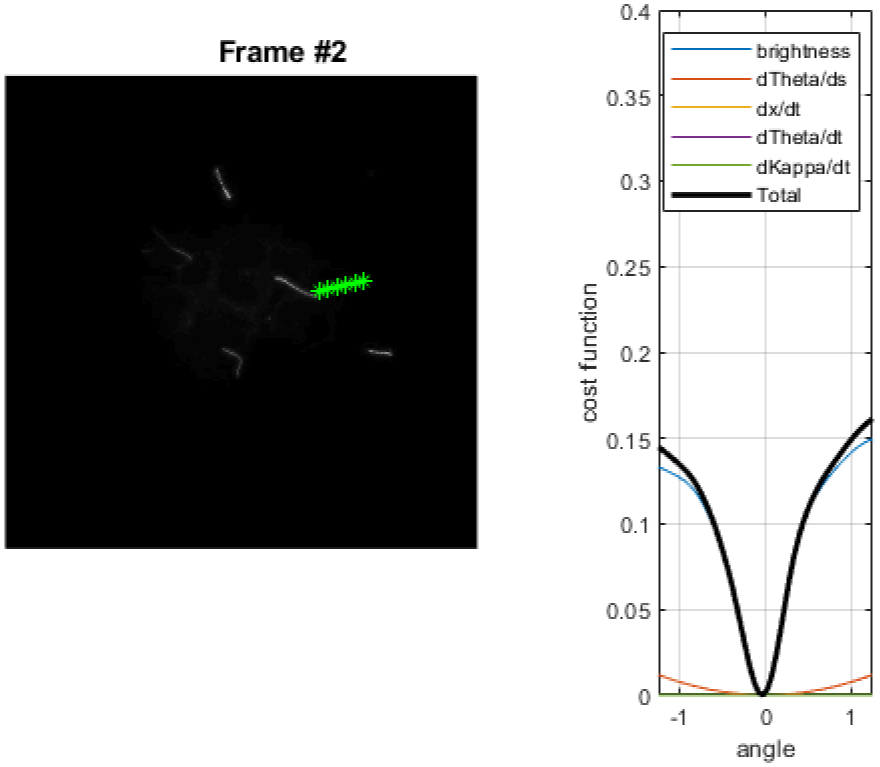
Frame #2
brightness
dTheta/ds
dx/dt
dTheta/dt
dKappa/dt
Total
cost function
angle

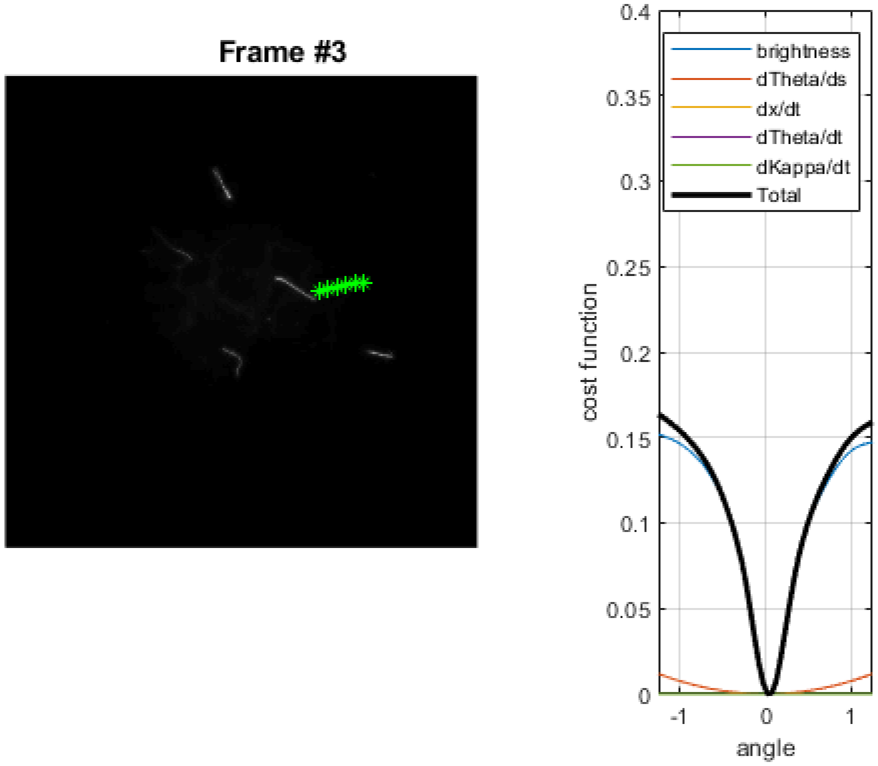
Frame #3
brightness
dTheta/ds
dx/dt
dTheta/dt
dKappa/dt
Total
cost function
angle
0.4
0.35
0.3
0.25
0.2
0.15
0.1
0.05
0
-1
0
1

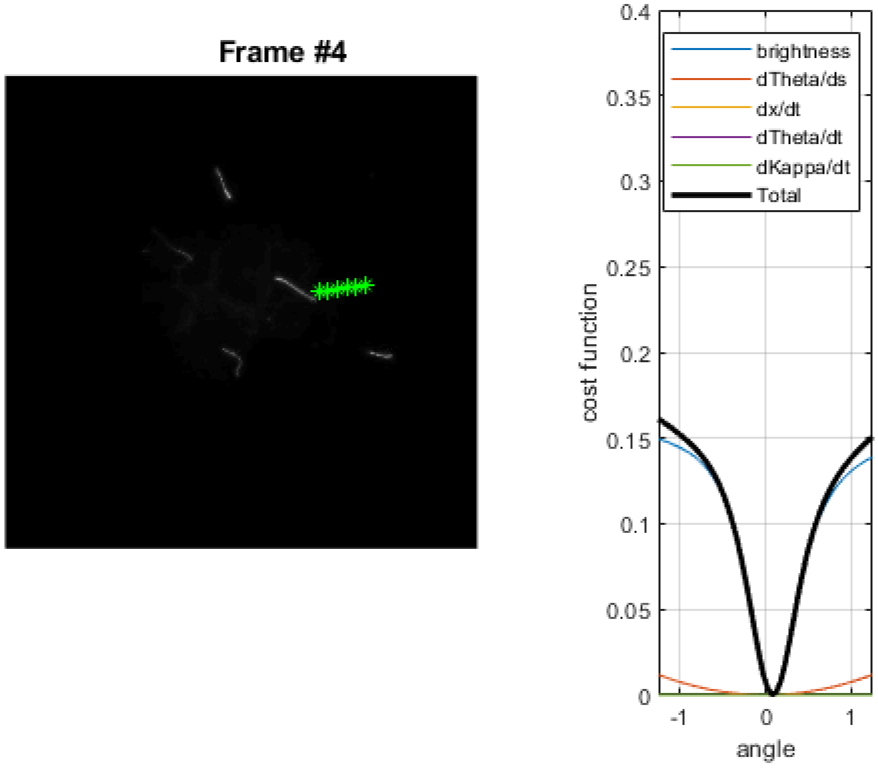
Frame #4
brightness
dTheta/ds
dx/dt
dTheta/dt
dKappa/dt
Total
cost function
angle
0.4
0.35
0.3
0.25
0.2
0.15
0.1
0.05
0
-1
0
1

Frame #5

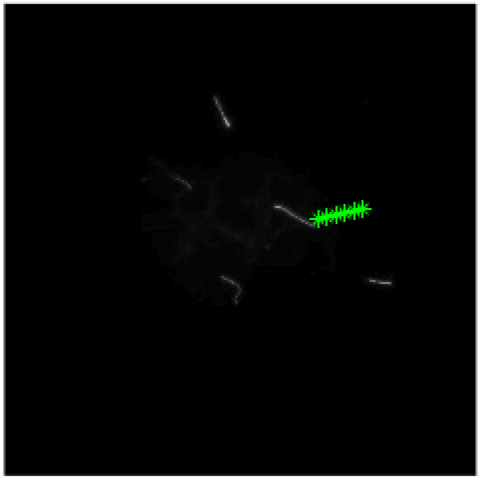

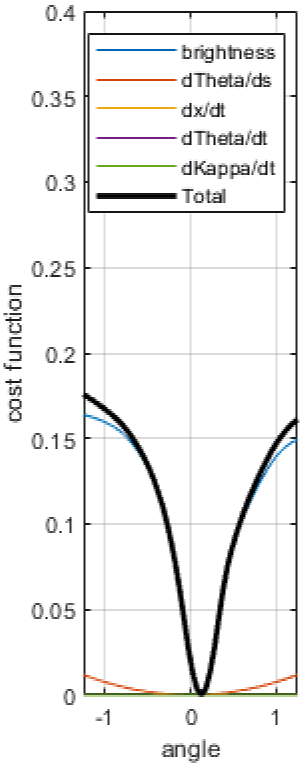

Frame #6

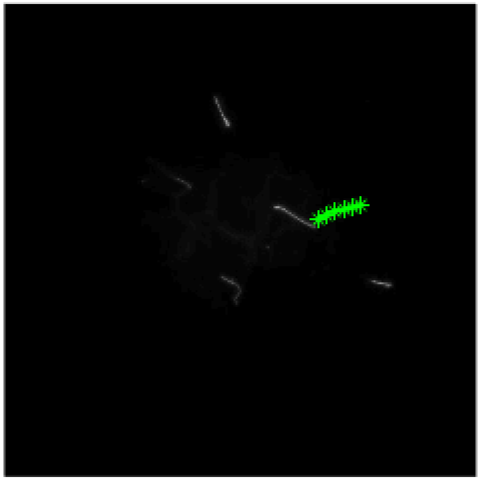

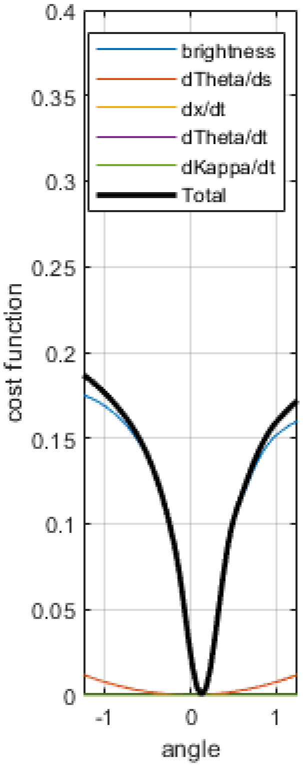

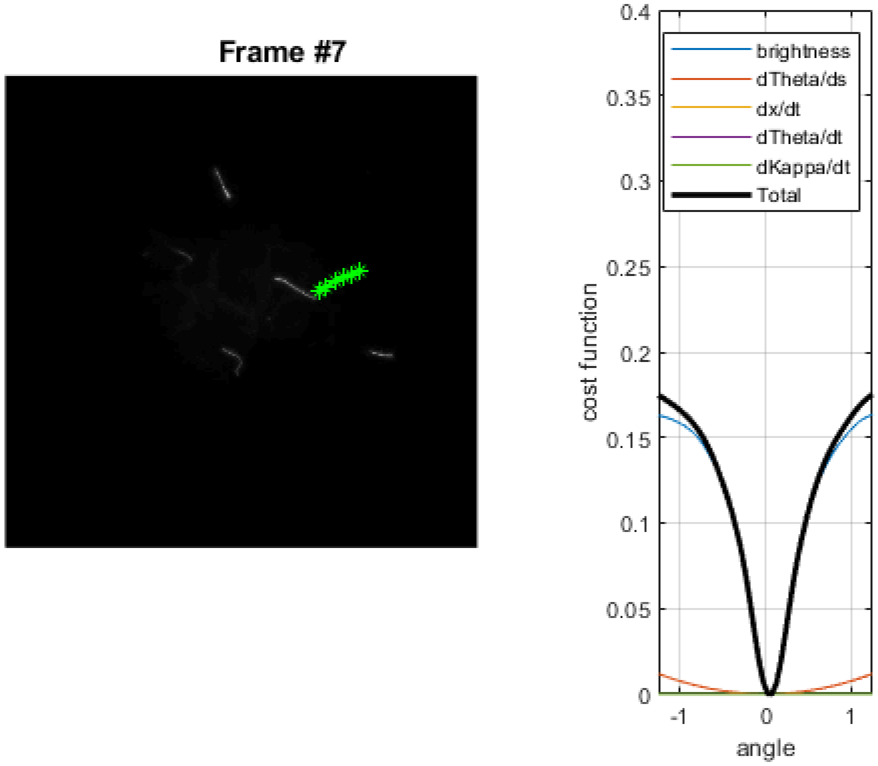
Frame #7
brightness
dTheta/ds
dx/dt
dTheta/dt
dKappa/dt
Total
cost function
angle
0
0.05
0.1
0.15
0.2
0.25
0.3
0.35
0.4
-1
0
1

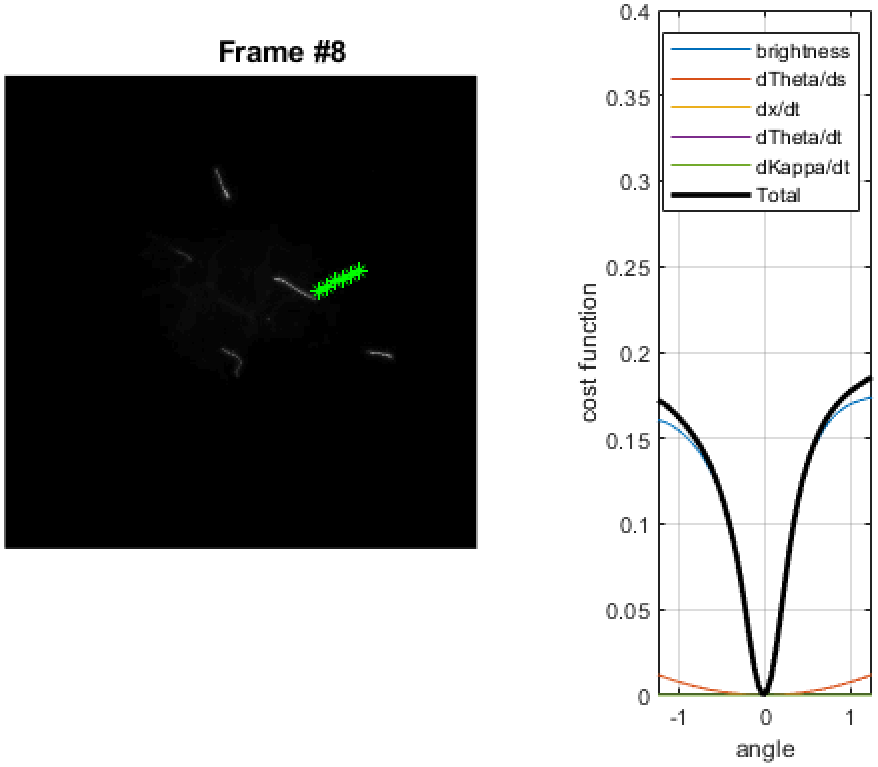
Frame #8
brightness
dTheta/ds
dx/dt
dTheta/dt
dKappa/dt
Total
cost function
angle
0
0.05
0.1
0.15
0.2
0.25
0.3
0.35
0.4
-1
0
1

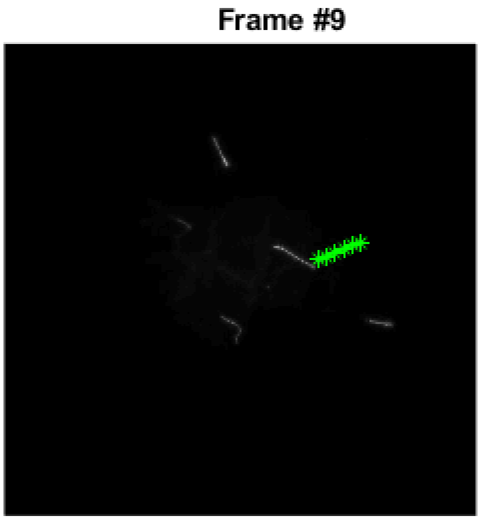
Frame #9

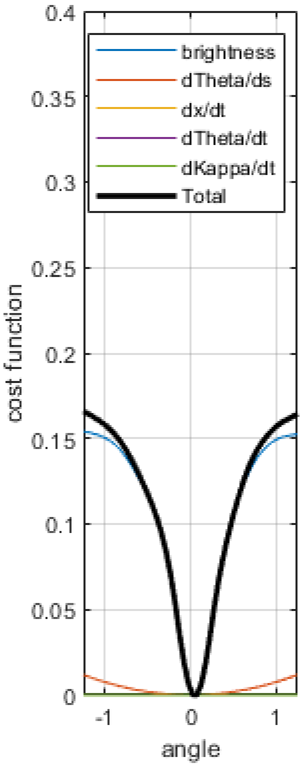
brightness
dTheta/ds
dx/dt
dTheta/dt
dKappa/dt
Total
cost function
angle
0.4
0.35
0.3
0.25
0.2
0.15
0.1
0.05
0
-1
0
1

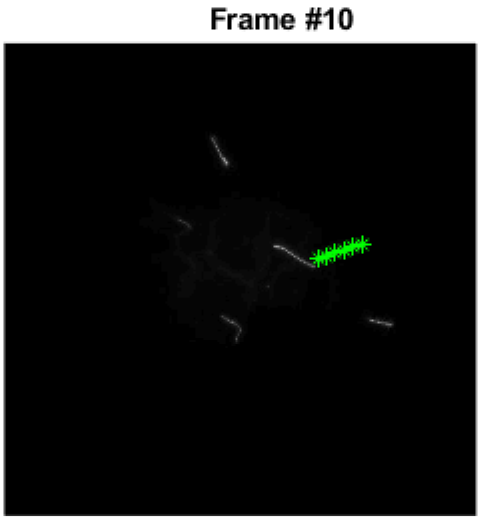
Frame #10

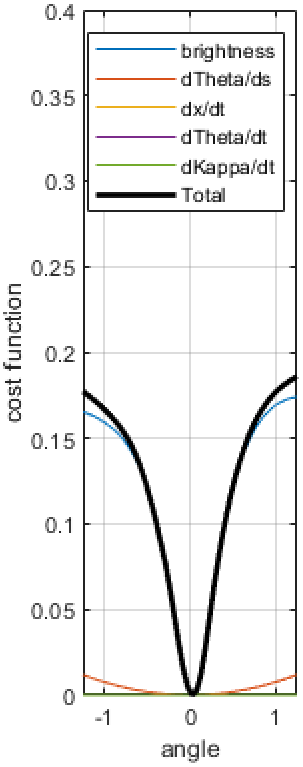
brightness
dTheta/ds
dx/dt
dTheta/dt
dKappa/dt
Total
cost function
angle
0.4
0.35
0.3
0.25
0.2
0.15
0.1
0.05
0
-1
0
1

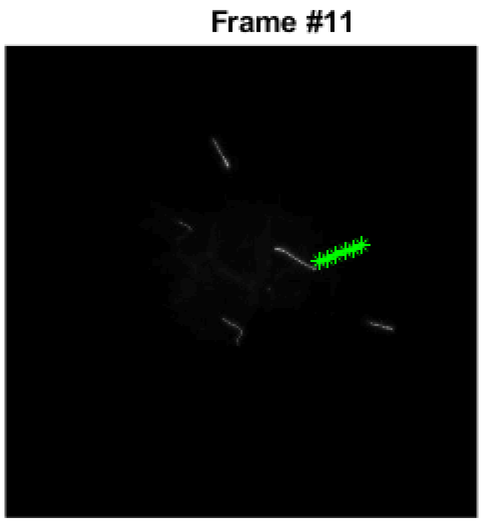
Frame #11

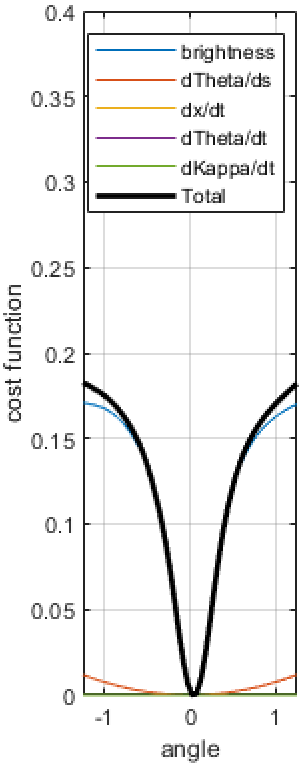
0.4
0.35
0.3
0.25
0.2
0.15
0.1
0.05
0
-1
0
1
angle
cost function
brightness
dTheta/ds
dx/dt
dTheta/dt
dKappa/dt
Total

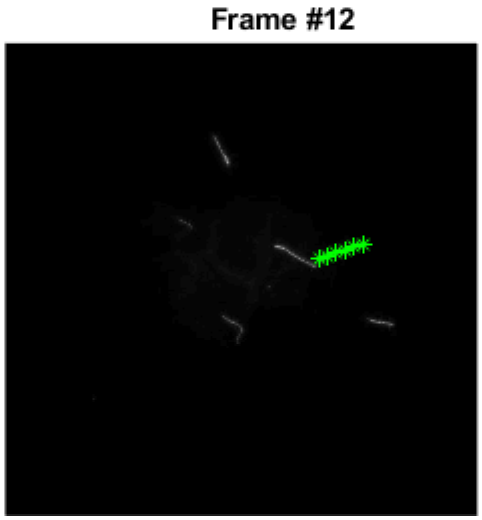
Frame #12

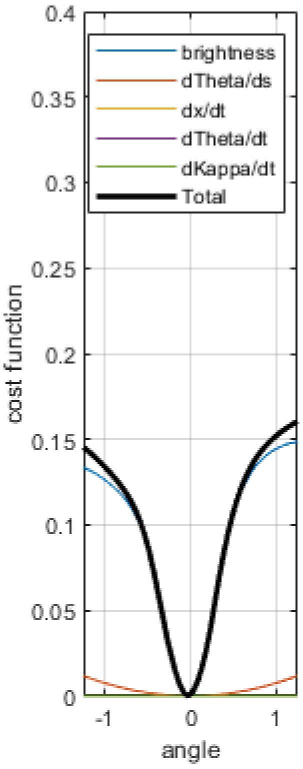
0.4
0.35
0.3
0.25
0.2
0.15
0.1
0.05
0
-1
0
1
angle
cost function
brightness
dTheta/ds
dx/dt
dTheta/dt
dKappa/dt
Total

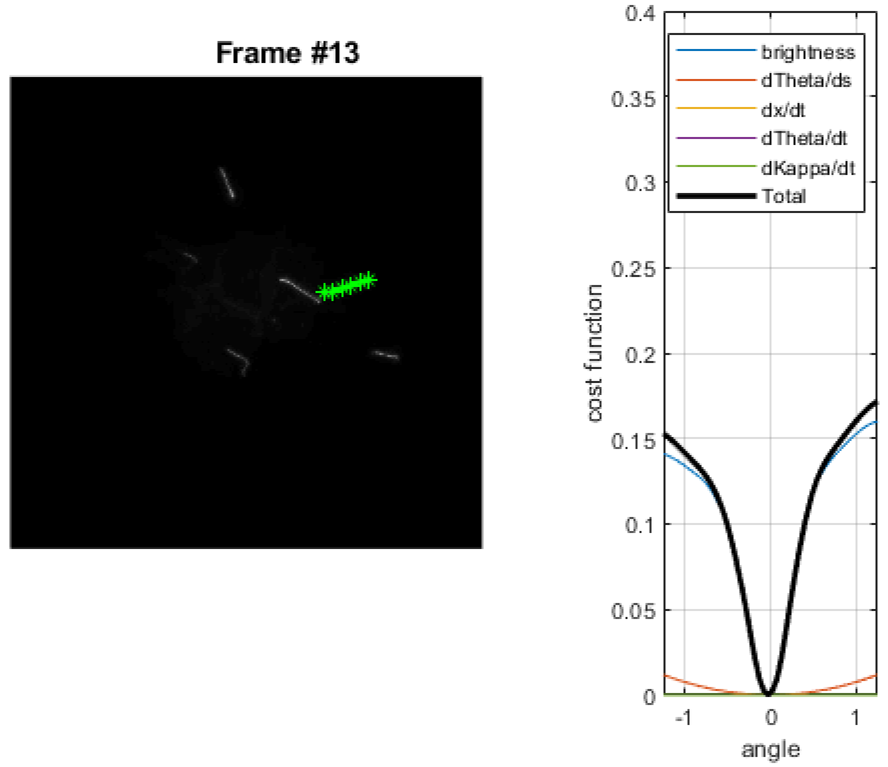
Frame #13
brightness
dTheta/ds
dx/dt
dTheta/dt
dKappa/dt
Total
cost function
angle
0.4
0.35
0.3
0.25
0.2
0.15
0.1
0.05
0
-1
0
1

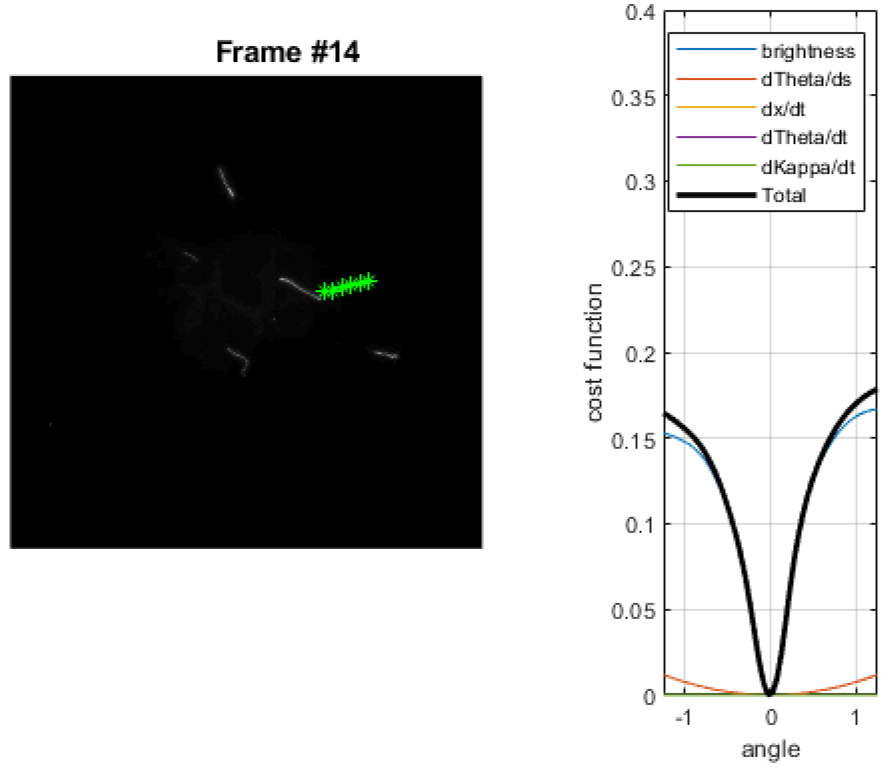
Frame #14
brightness
dTheta/ds
dx/dt
dTheta/dt
dKappa/dt
Total
cost function
angle
0.4
0.35
0.3
0.25
0.2
0.15
0.1
0.05
0
-1
0
1

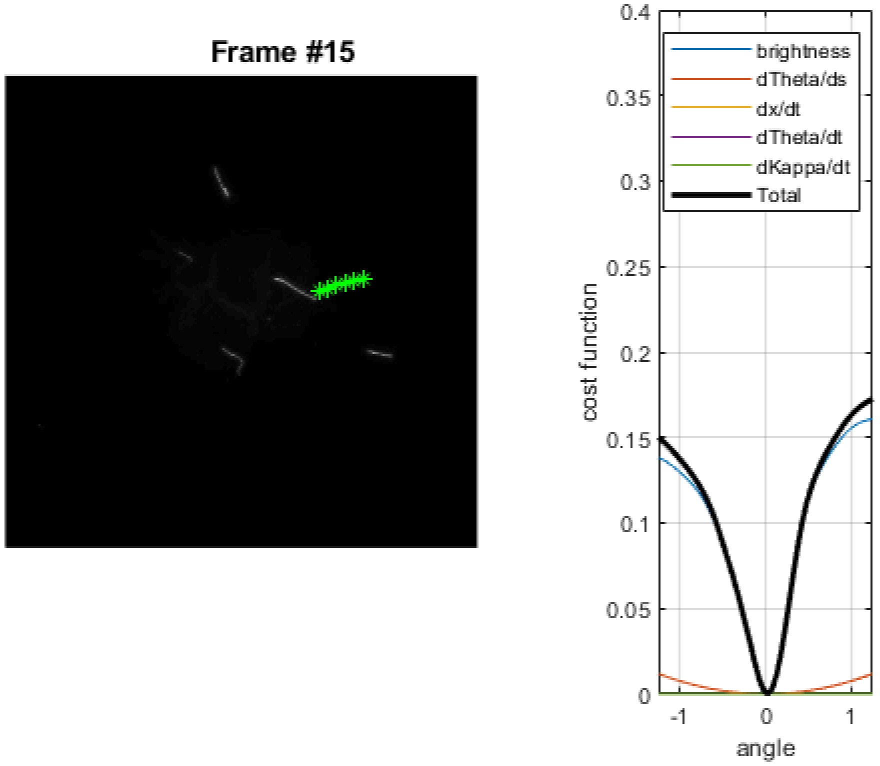
Frame #15
0.4
0.35
0.3
0.25
0.2
0.15
0.1
0.05
0
cost function
brightness
dTheta/ds
dx/dt
dTheta/dt
dKappa/dt
Total
-1
0
1
angle

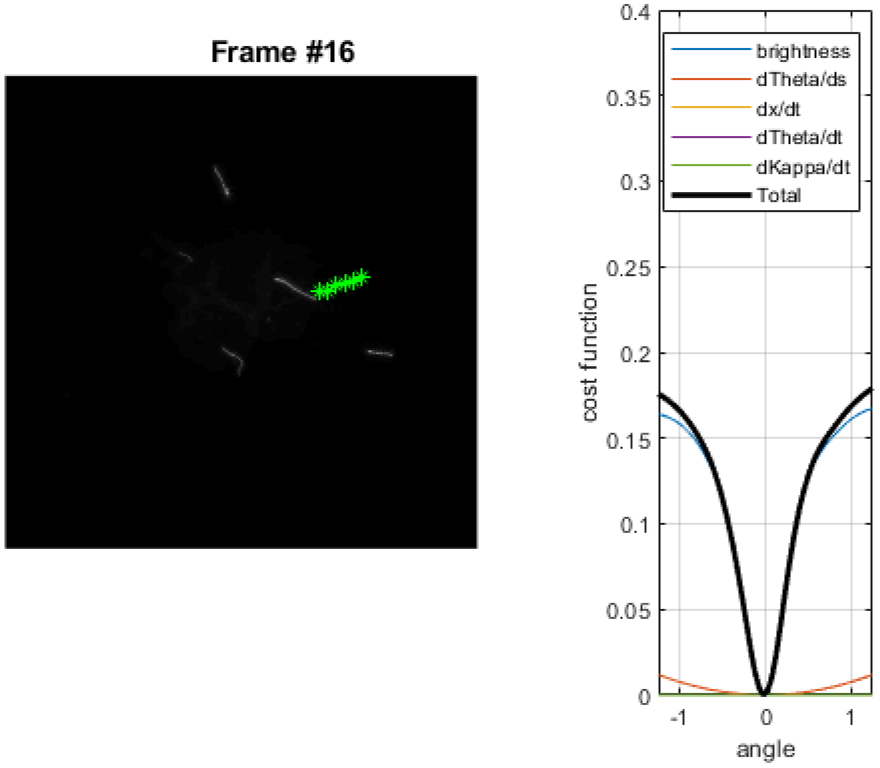
Frame #16
0.4
0.35
0.3
0.25
0.2
0.15
0.1
0.05
0
cost function
brightness
dTheta/ds
dx/dt
dTheta/dt
dKappa/dt
Total
-1
0
1
angle

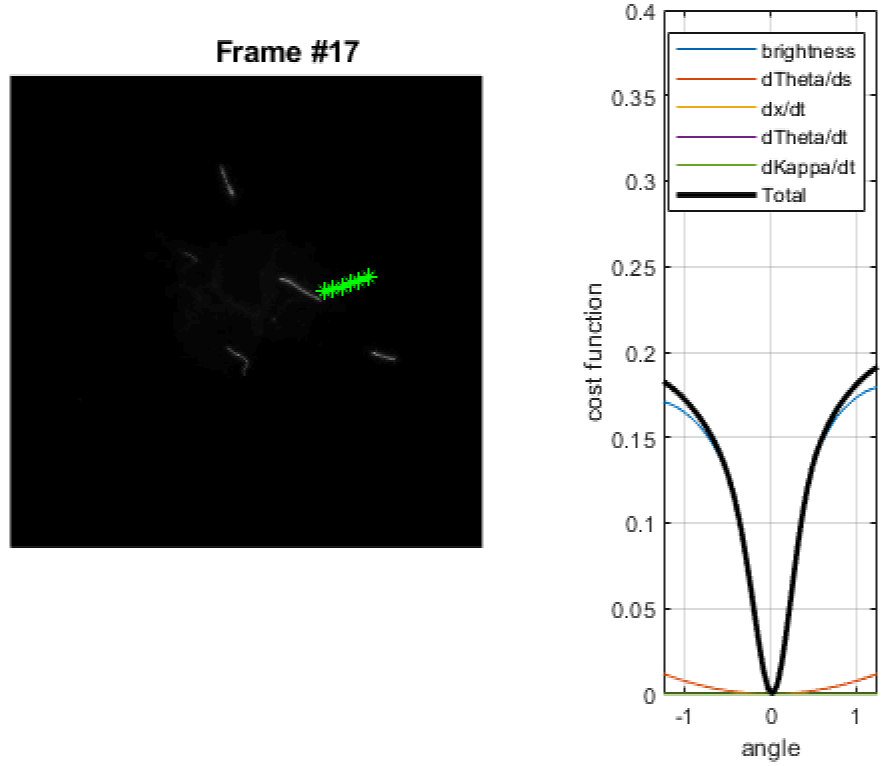
Frame #17
brightness
dTheta/ds
dx/dt
dTheta/dt
dKappa/dt
Total
cost function
angle
0.4
0.35
0.3
0.25
0.2
0.15
0.1
0.05
0
-1
0
1

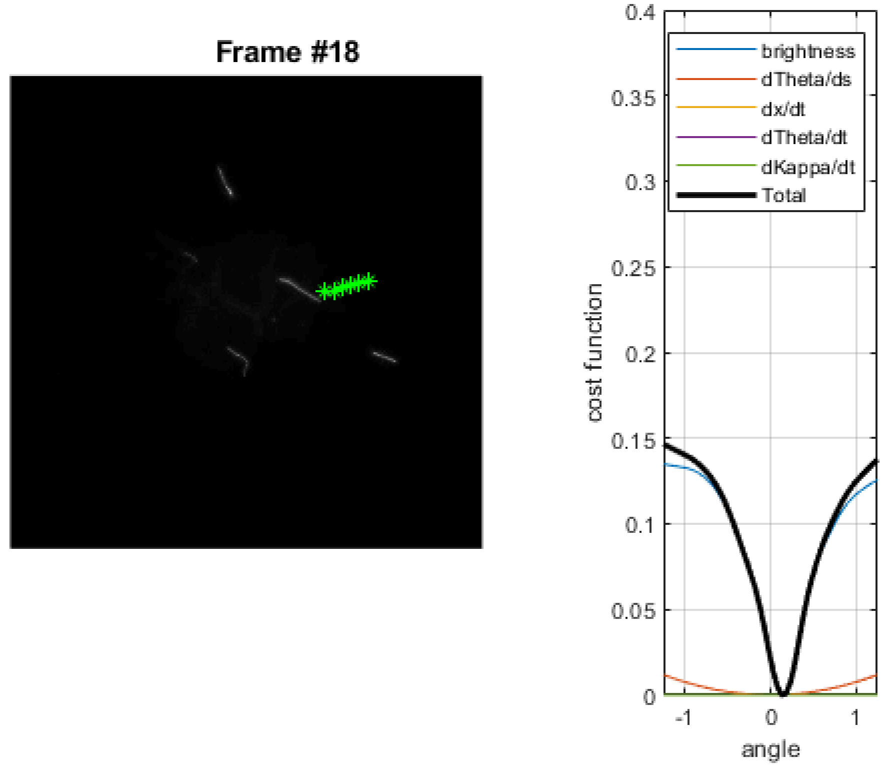
Frame #18
brightness
dTheta/ds
dx/dt
dTheta/dt
dKappa/dt
Total
cost function
angle
0.4
0.35
0.3
0.25
0.2
0.15
0.1
0.05
0
-1
0
1

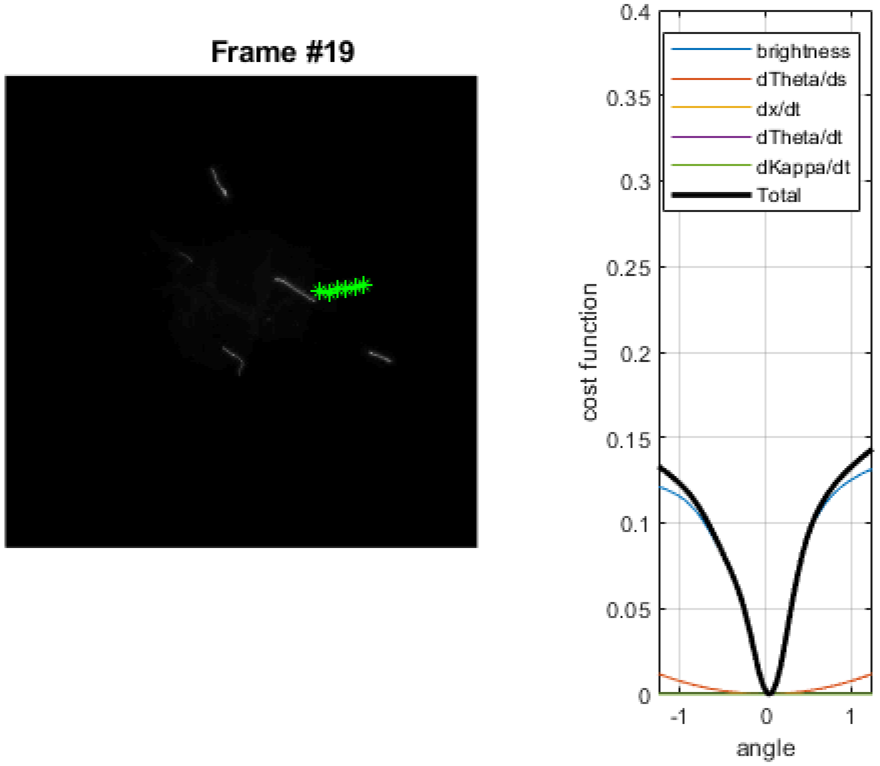
Frame #19
0.4
0.35
0.3
0.25
0.2
0.15
0.1
0.05
0
-1
0
1
angle
cost function
brightness
dTheta/ds
dx/dt
dTheta/dt
dKappa/dt
Total

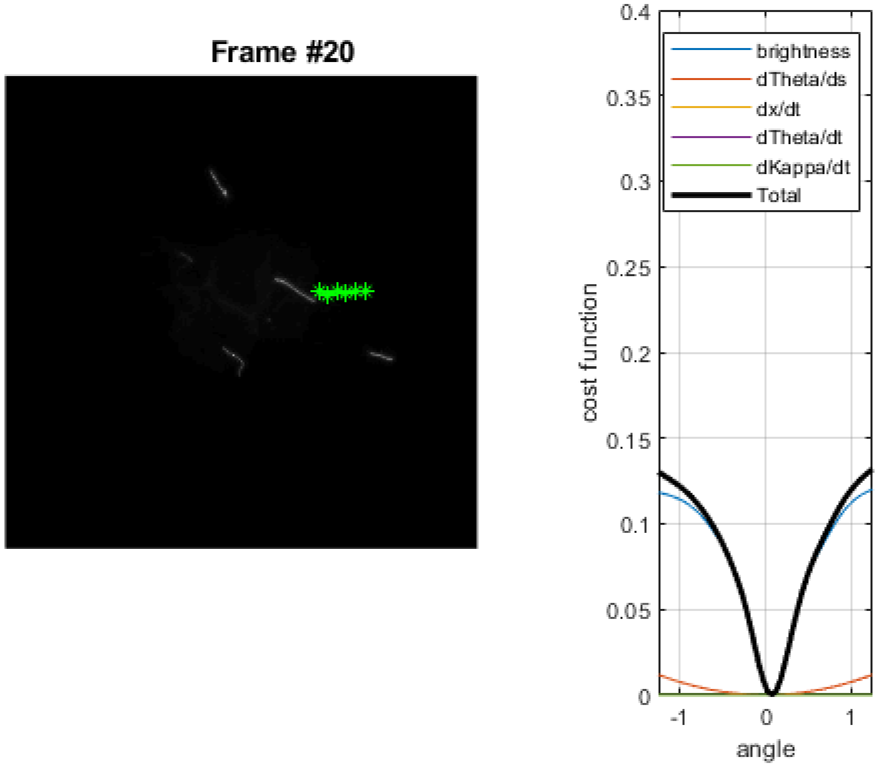
Frame #20
0.4
0.35
0.3
0.25
0.2
0.15
0.1
0.05
0
-1
0
1
angle
cost function
brightness
dTheta/ds
dx/dt
dTheta/dt
dKappa/dt
Total

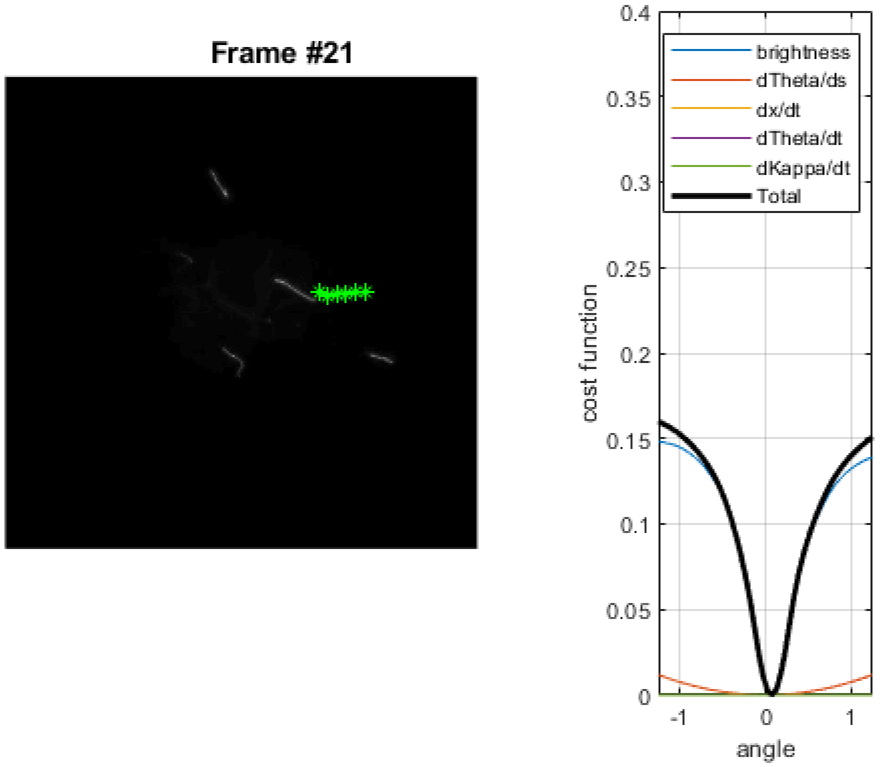
Frame #21
brightness
dTheta/ds
dx/dt
dTheta/dt
dKappa/dt
Total
cost function
angle
0.4
0.35
0.3
0.25
0.2
0.15
0.1
0.05
0
-1
0
1

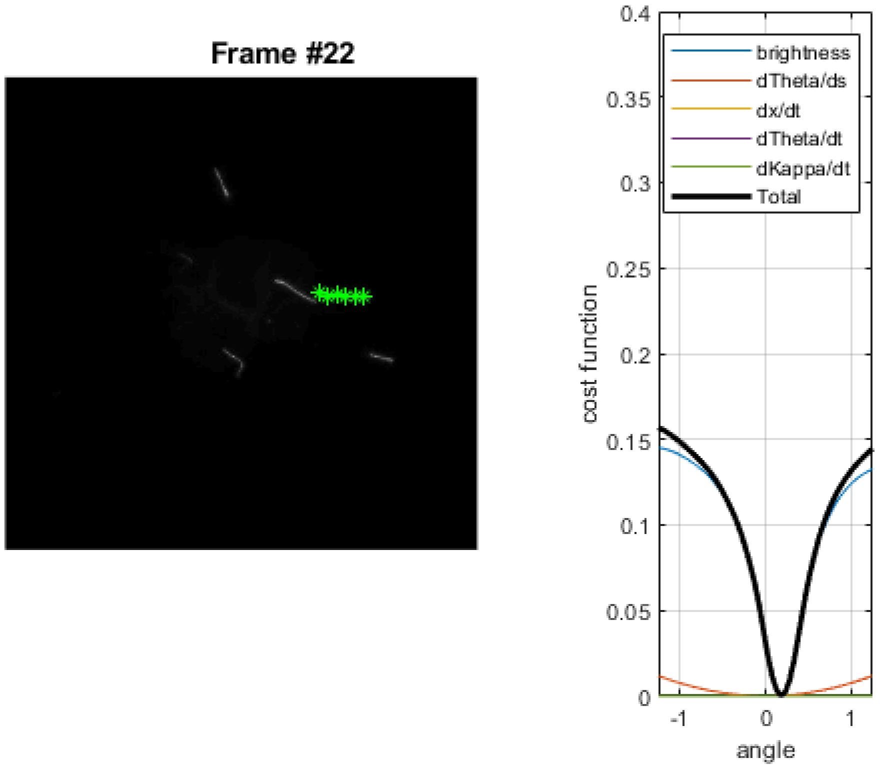
Frame #22
brightness
dTheta/ds
dx/dt
dTheta/dt
dKappa/dt
Total
cost function
angle
0.4
0.35
0.3
0.25
0.2
0.15
0.1
0.05
0
-1
0
1

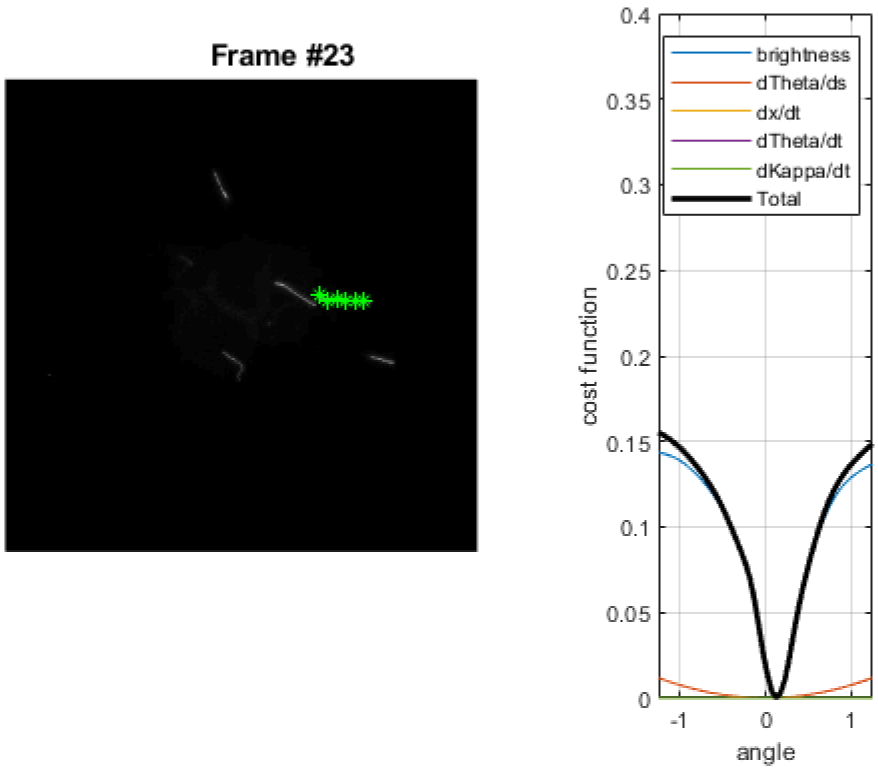
Frame #23
brightness
dTheta/ds
dx/dt
dTheta/dt
dKappa/dt
Total
cost function
angle
0.4
0.35
0.3
0.25
0.2
0.15
0.1
0.05
0
-1
0
1

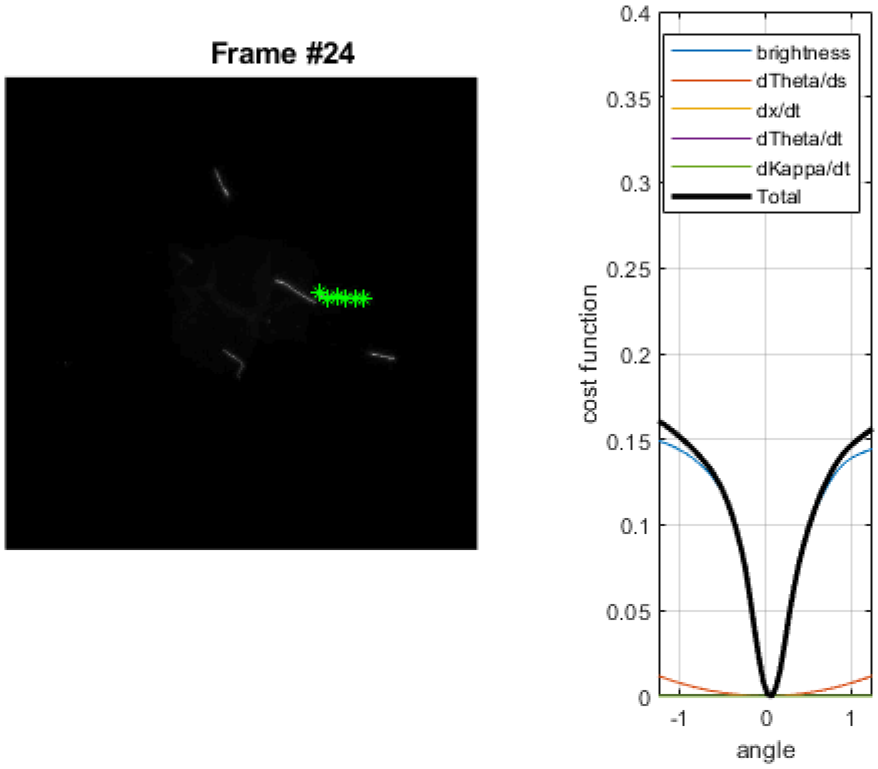
Frame #24
brightness
dTheta/ds
dx/dt
dTheta/dt
dKappa/dt
Total
cost function
angle
0.4
0.35
0.3
0.25
0.2
0.15
0.1
0.05
0
-1
0
1

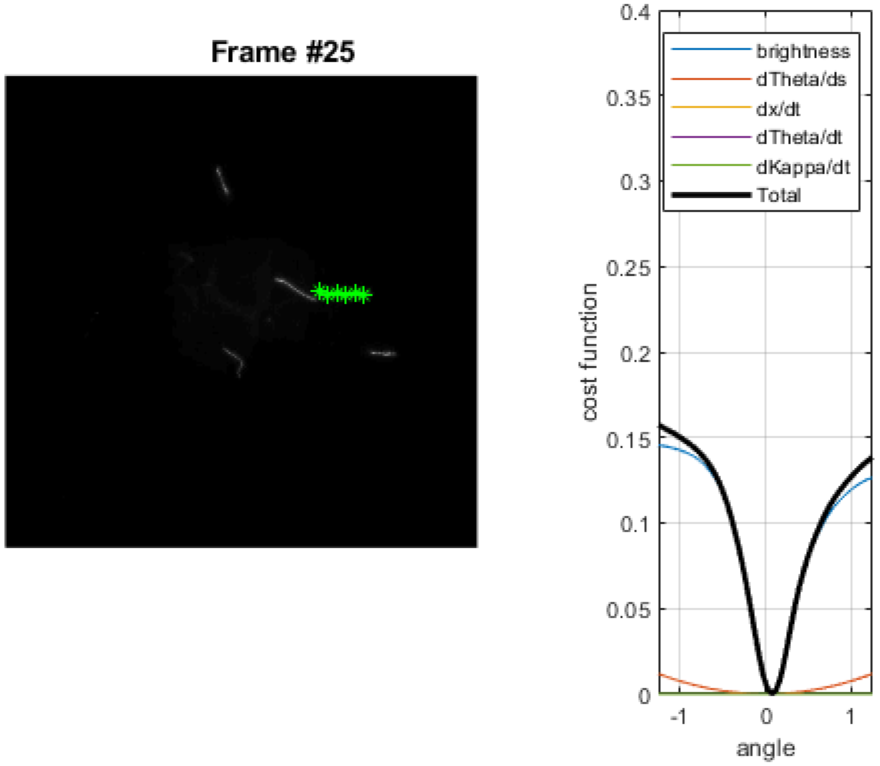
Frame #25
brightness
dTheta/ds
dx/dt
dTheta/dt
dKappa/dt
Total
cost function
angle
0.4
0.35
0.3
0.25
0.2
0.15
0.1
0.05
0
-1
0
1

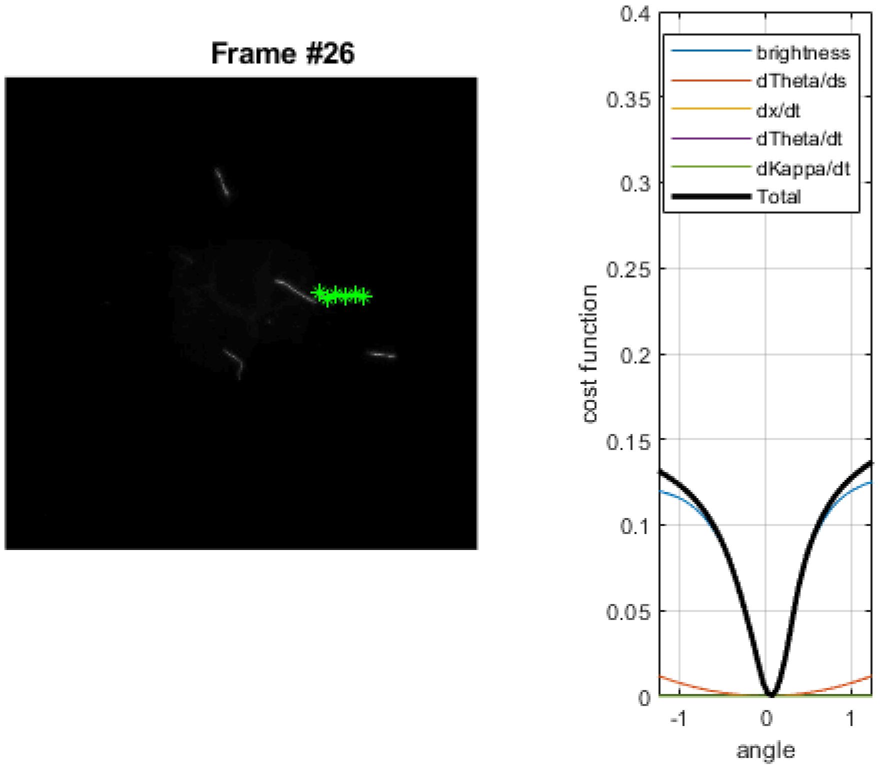
Frame #26
brightness
dTheta/ds
dx/dt
dTheta/dt
dKappa/dt
Total
cost function
angle
0.4
0.35
0.3
0.25
0.2
0.15
0.1
0.05
0
-1
0
1

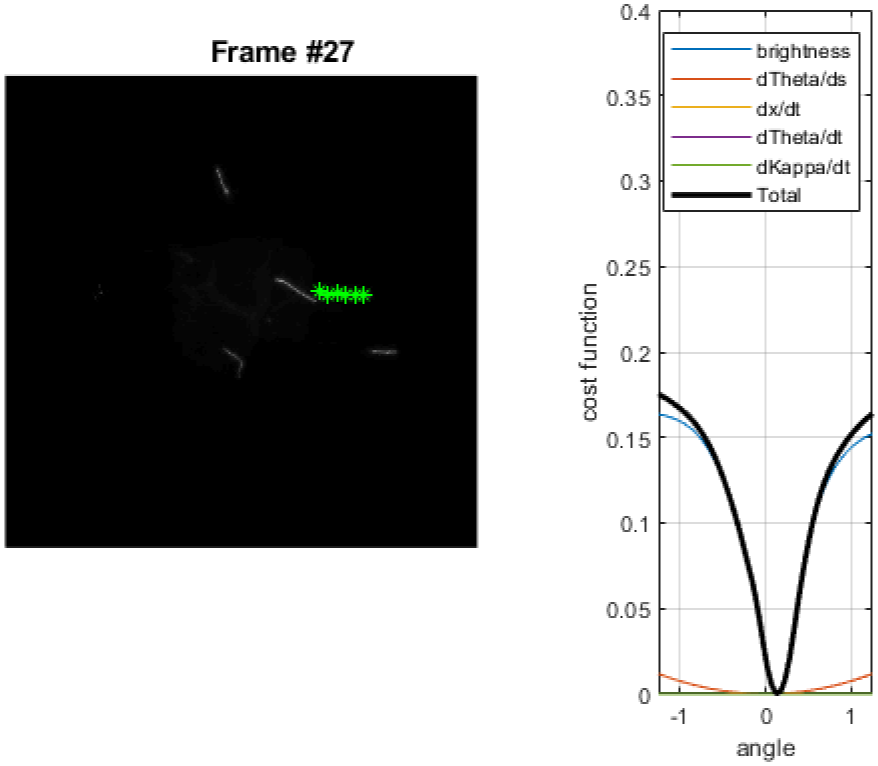
Frame #27
brightness
dTheta/ds
dx/dt
dTheta/dt
dKappa/dt
Total
cost function
angle

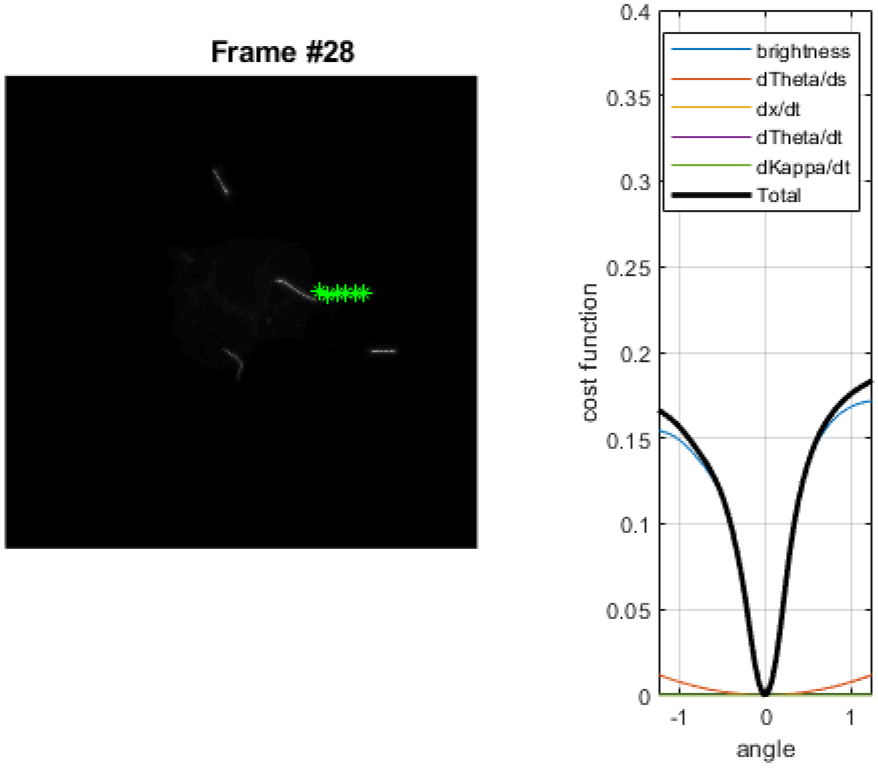
Frame #28
brightness
dTheta/ds
dx/dt
dTheta/dt
dKappa/dt
Total
cost function
angle

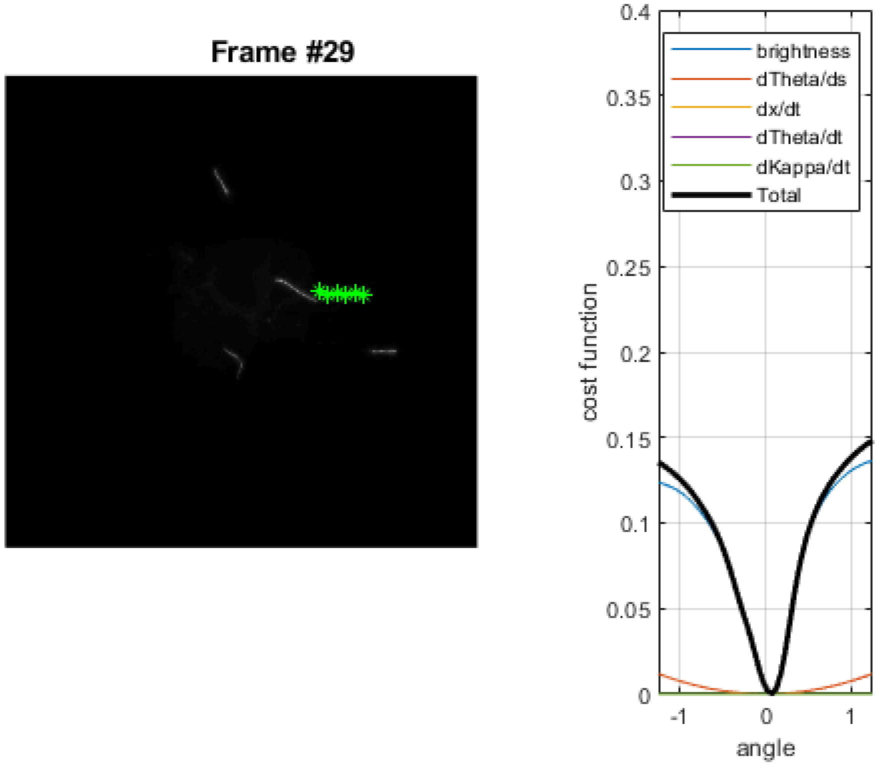
Frame #29
brightness
dTheta/ds
dx/dt
dTheta/dt
dKappa/dt
Total
cost function
angle

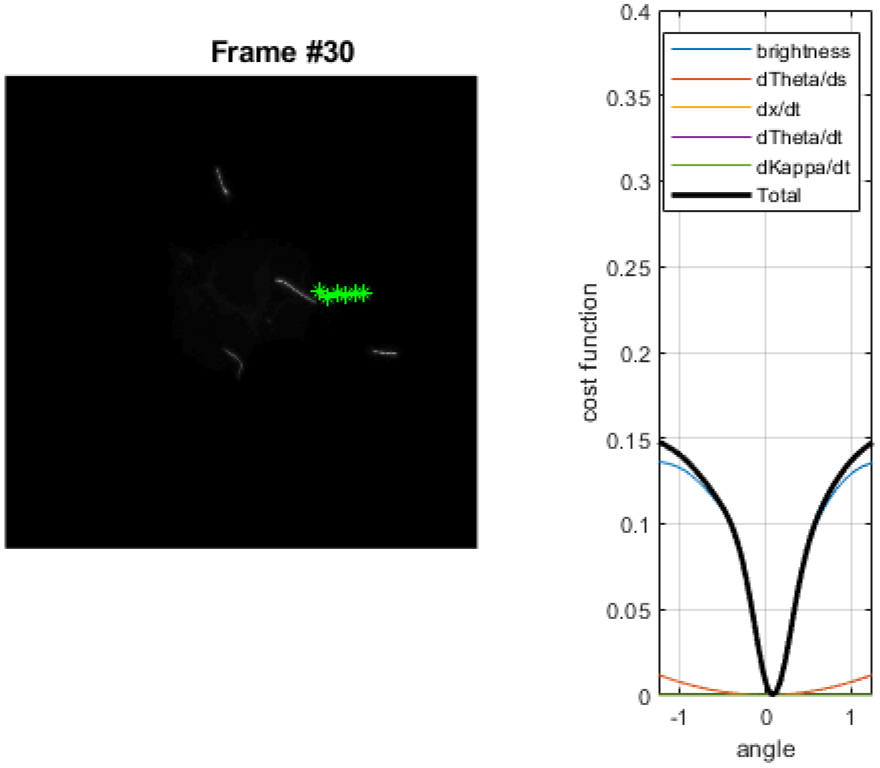
Frame #30
brightness
dTheta/ds
dx/dt
dTheta/dt
dKappa/dt
Total
cost function
angle

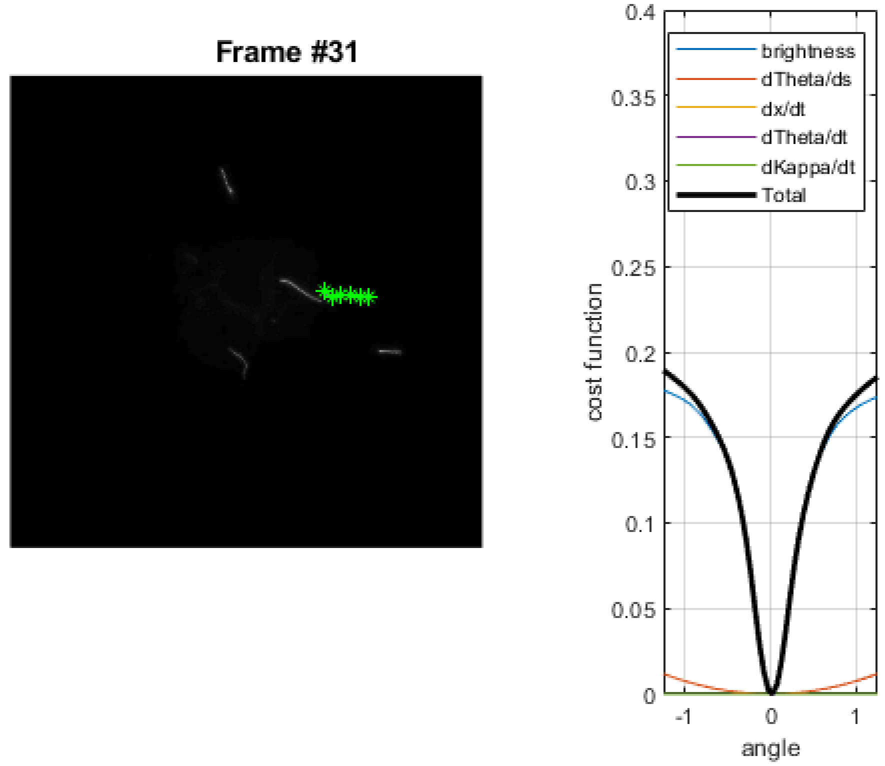
Frame #31
brightness
dTheta/ds
dx/dt
dTheta/dt
dKappa/dt
Total
cost function
angle

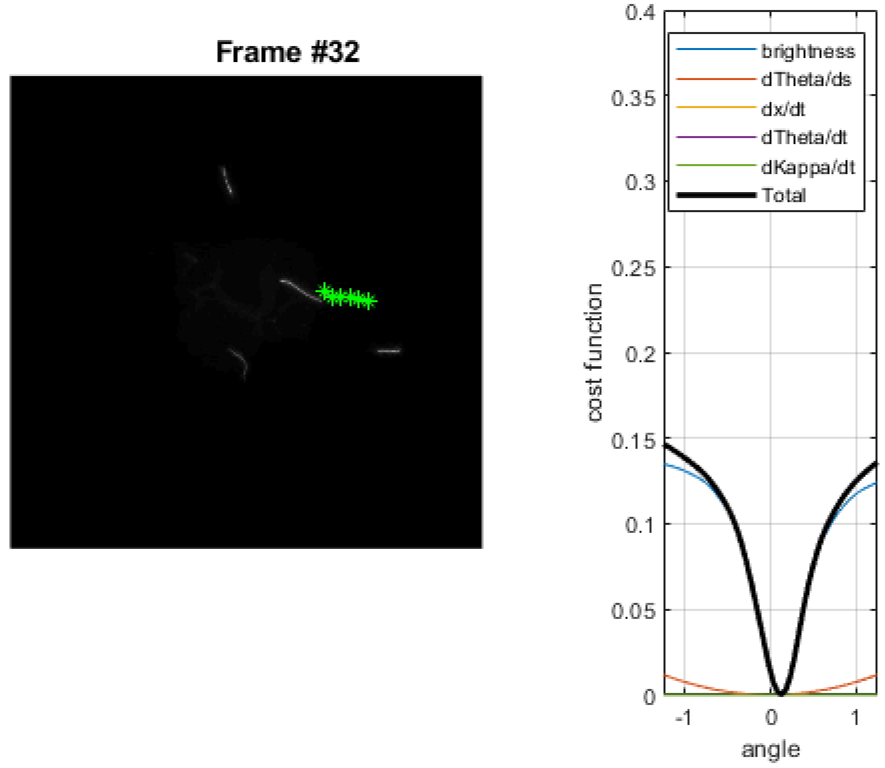
Frame #32
brightness
dTheta/ds
dx/dt
dTheta/dt
dKappa/dt
Total
cost function
angle

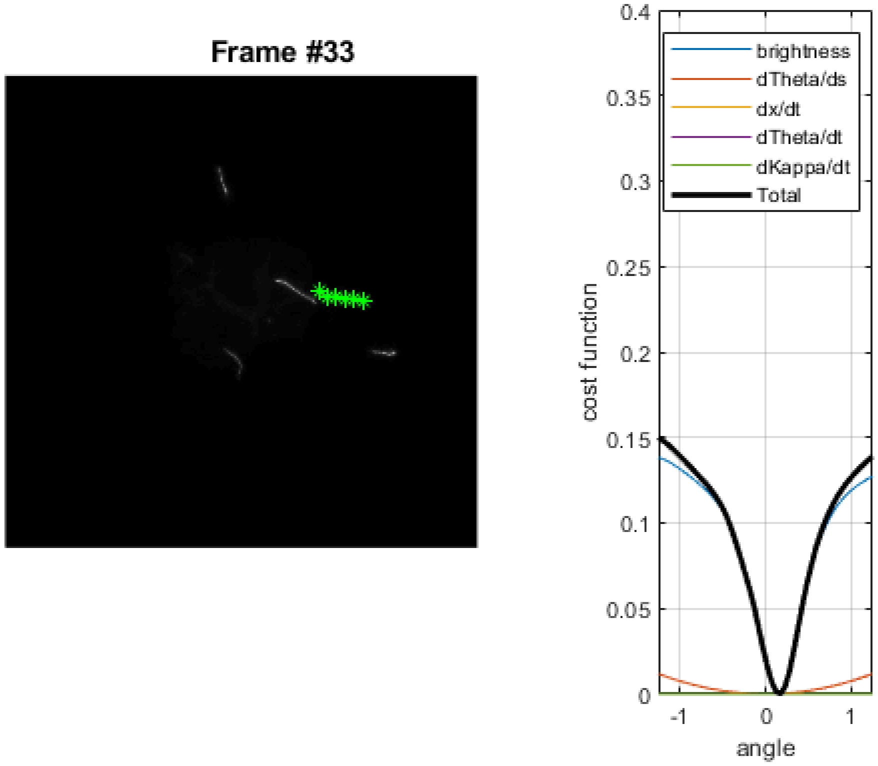
Frame #33
0.4
0.35
0.3
0.25
0.2
0.15
0.1
0.05
0
cost function
brightness
dTheta/ds
dx/dt
dTheta/dt
dKappa/dt
Total
-1
0
1
angle

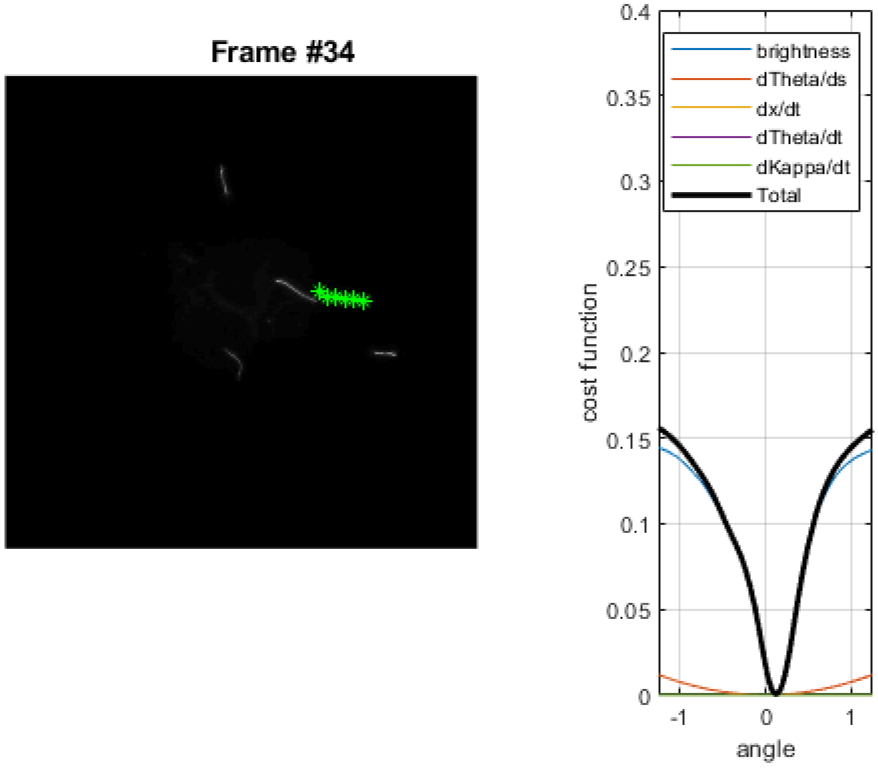
Frame #34
0.4
0.35
0.3
0.25
0.2
0.15
0.1
0.05
0
cost function
brightness
dTheta/ds
dx/dt
dTheta/dt
dKappa/dt
Total
-1
0
1
angle

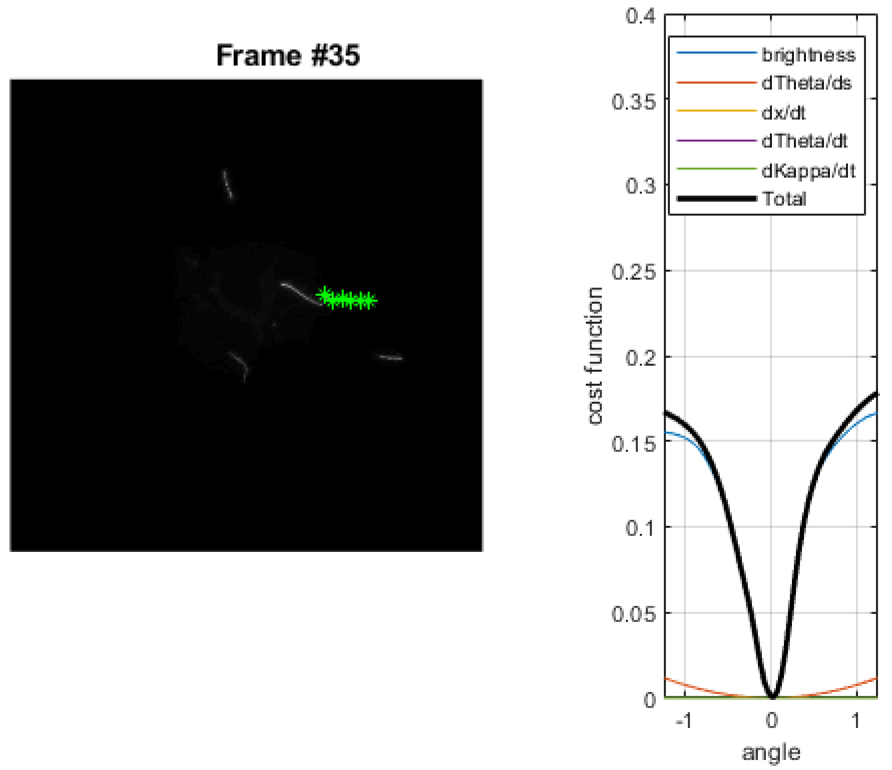
Frame #35
brightness
dTheta/ds
dx/dt
dTheta/dt
dKappa/dt
Total
cost function
angle

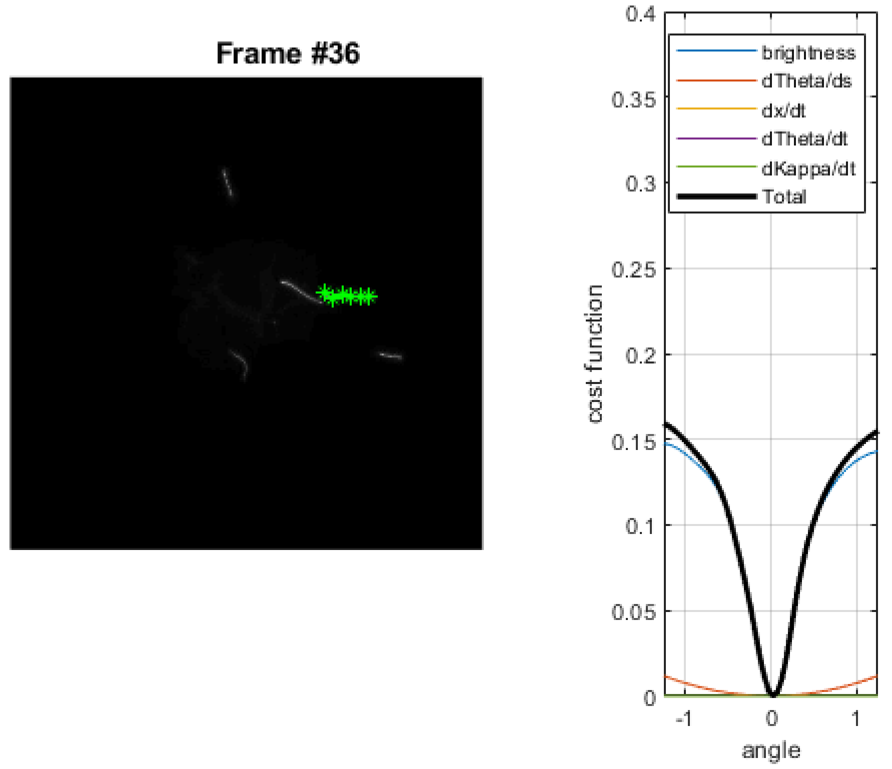
Frame #36
brightness
dTheta/ds
dx/dt
dTheta/dt
dKappa/dt
Total
cost function
angle

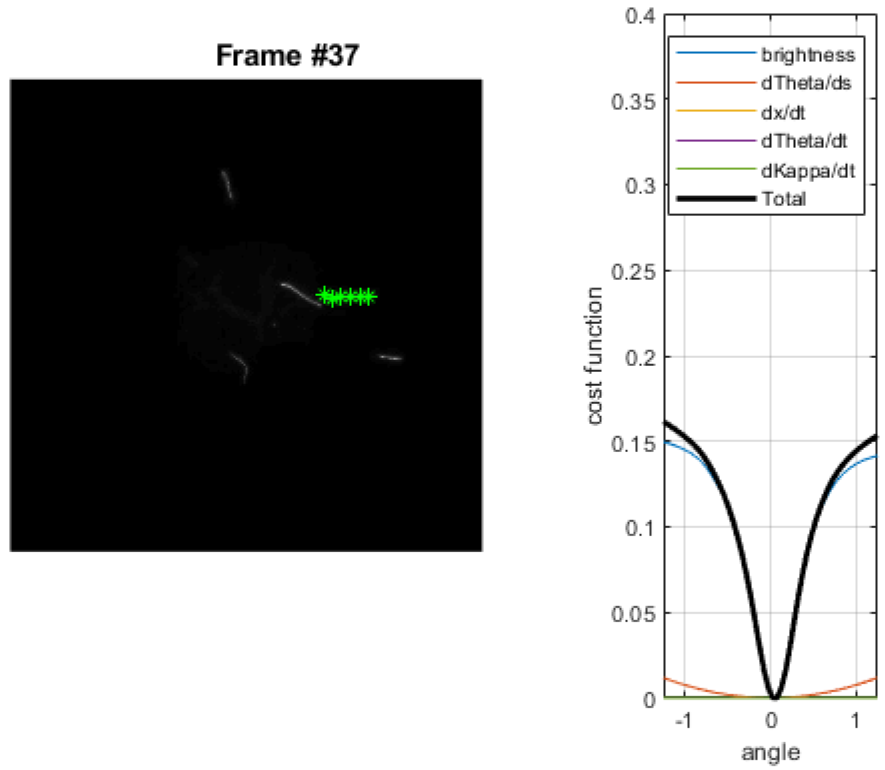
Frame #37
brightness
dTheta/ds
dx/dt
dTheta/dt
dKappa/dt
Total
cost function
angle
0.4
0.35
0.3
0.25
0.2
0.15
0.1
0.05
0
-1
0
1

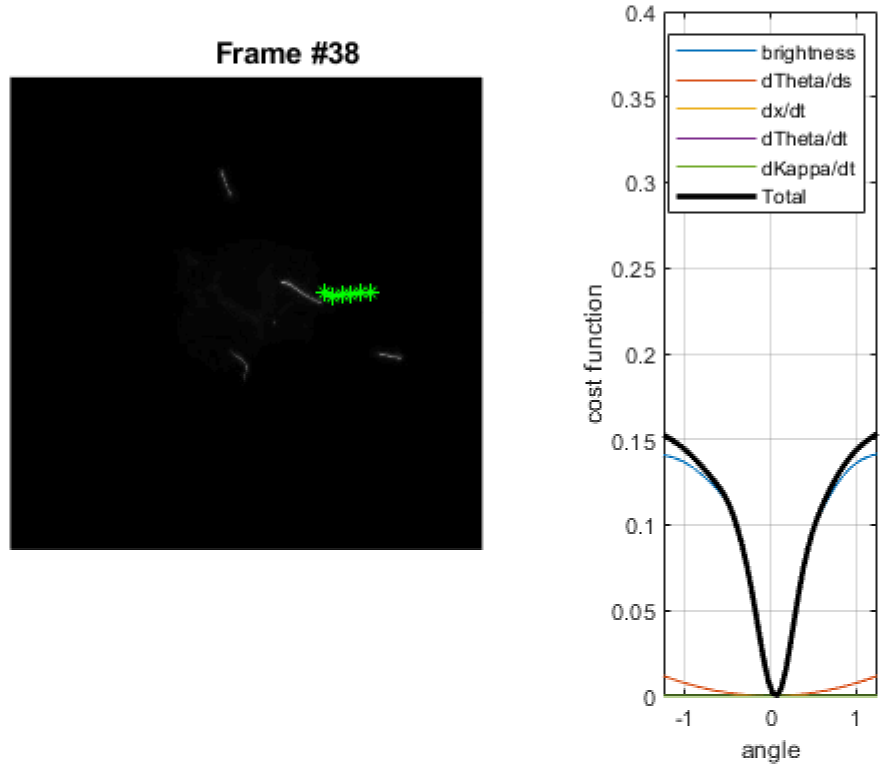
Frame #38
brightness
dTheta/ds
dx/dt
dTheta/dt
dKappa/dt
Total
cost function
angle
0.4
0.35
0.3
0.25
0.2
0.15
0.1
0.05
0
-1
0
1

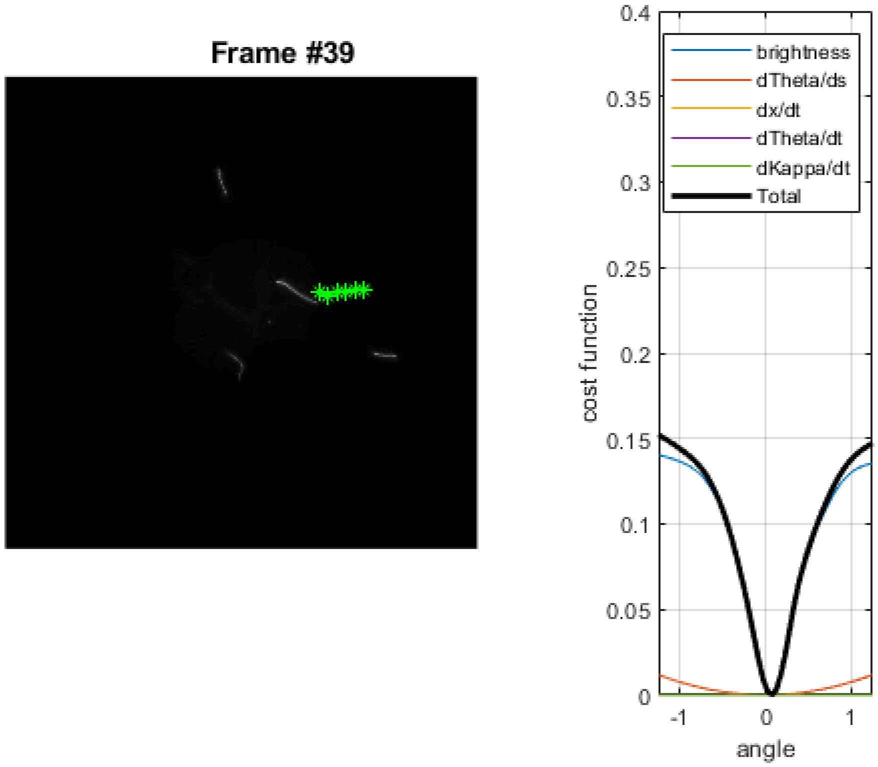
Frame #39
brightness
dTheta/ds
dx/dt
dTheta/dt
dKappa/dt
Total
cost function
angle

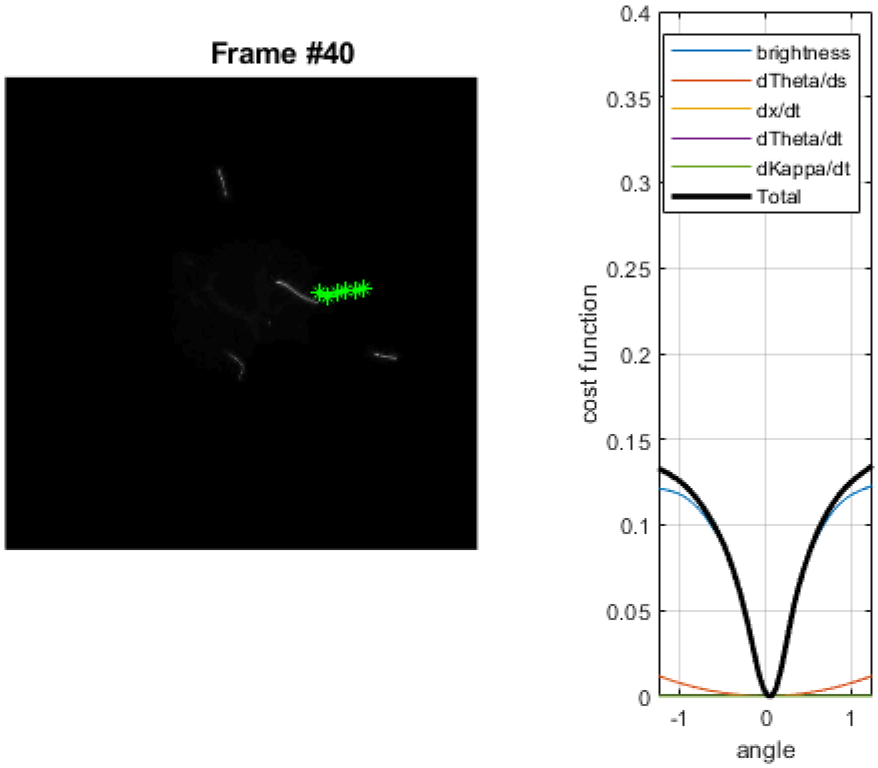
Frame #40
brightness
dTheta/ds
dx/dt
dTheta/dt
dKappa/dt
Total
cost function
angle

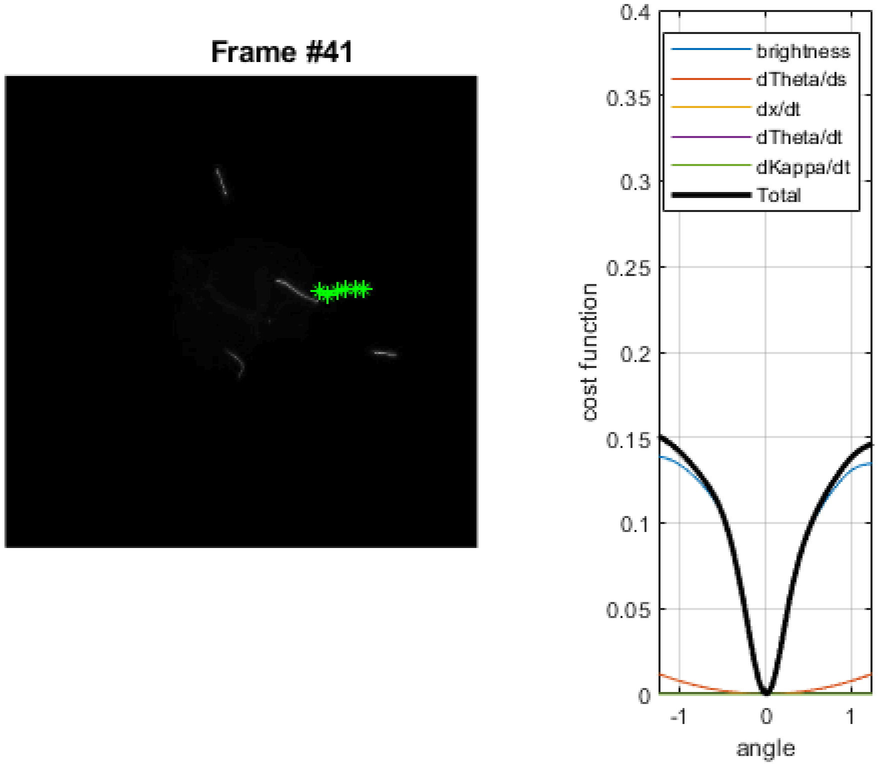
Frame #41
brightness
dTheta/ds
dx/dt
dTheta/dt
dKappa/dt
Total
cost function
angle
0.4
0.35
0.3
0.25
0.2
0.15
0.1
0.05
0
-1
0
1

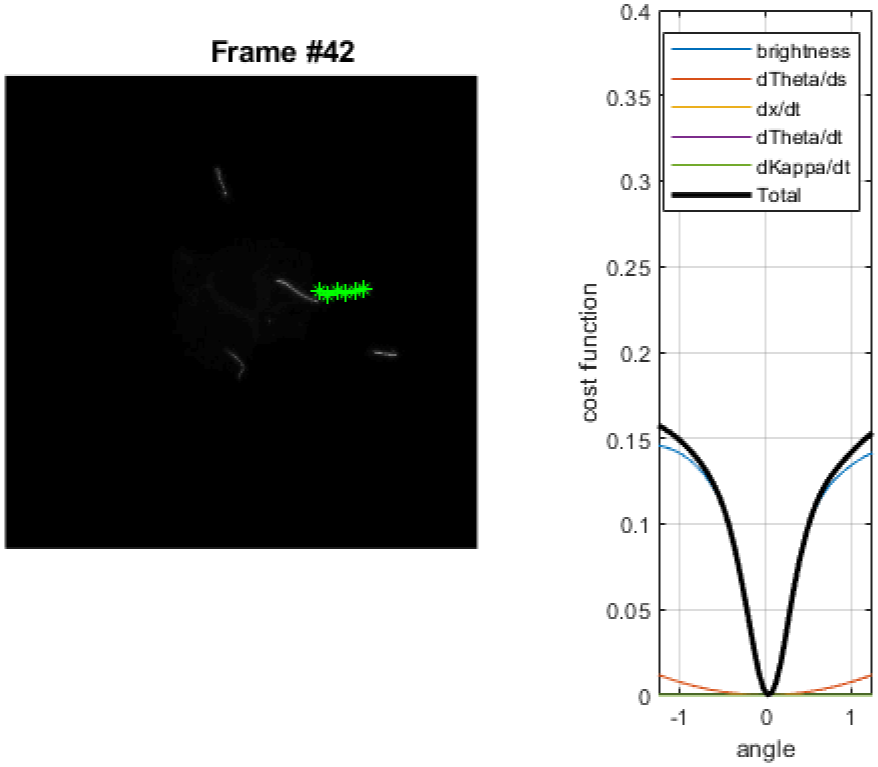
Frame #42
brightness
dTheta/ds
dx/dt
dTheta/dt
dKappa/dt
Total
cost function
angle
0.4
0.35
0.3
0.25
0.2
0.15
0.1
0.05
0
-1
0
1

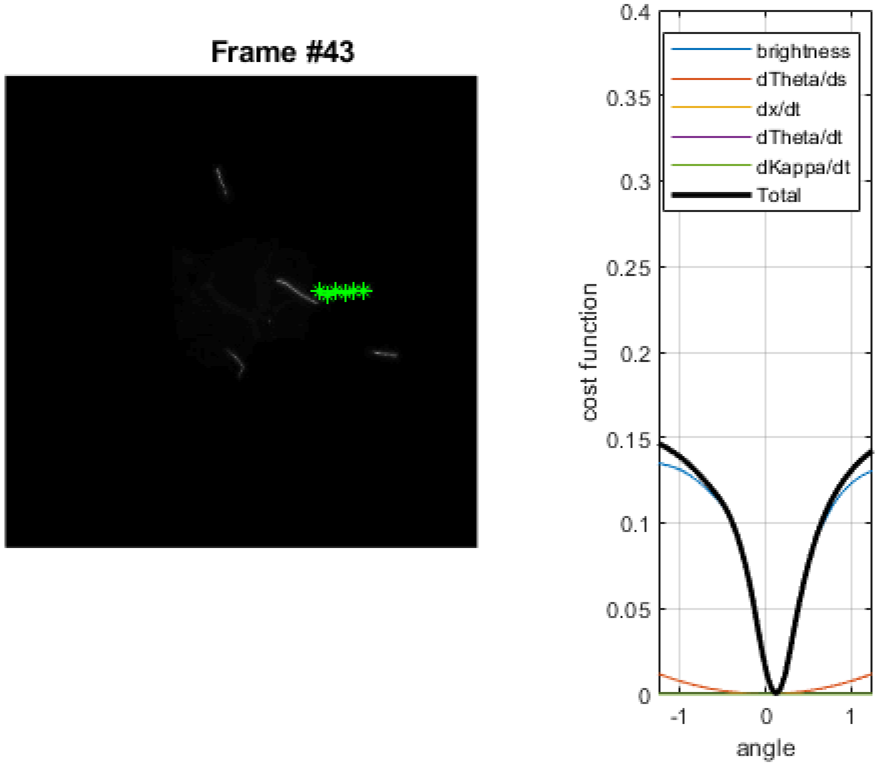
Frame #43
brightness
dTheta/ds
dx/dt
dTheta/dt
dKappa/dt
Total
cost function
angle

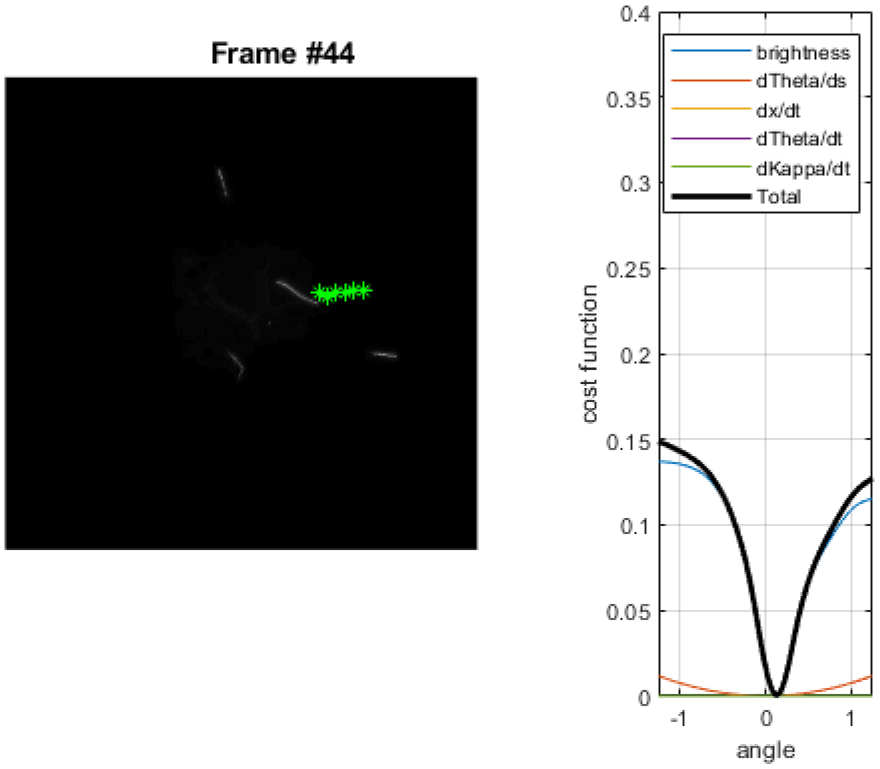
Frame #44
brightness
dTheta/ds
dx/dt
dTheta/dt
dKappa/dt
Total
cost function
angle

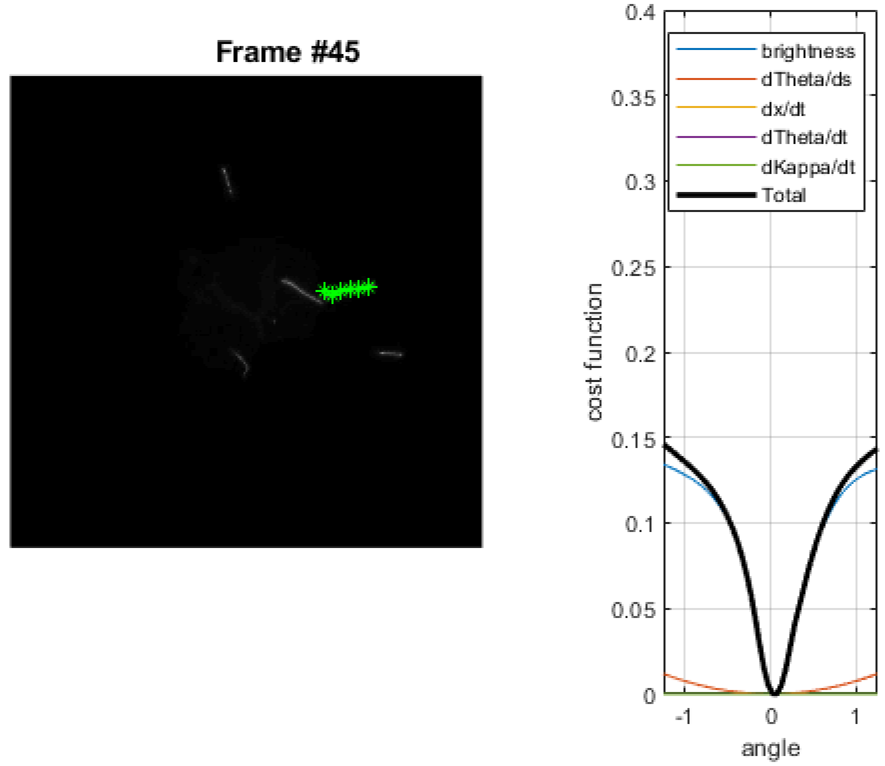

Frame #45
brightness
dTheta/ds
dx/dt
dTheta/dt
dKappa/dt
Total
cost function
angle
0.4
0.35
0.3
0.25
0.2
0.15
0.1
0.05
0
-1
0
1

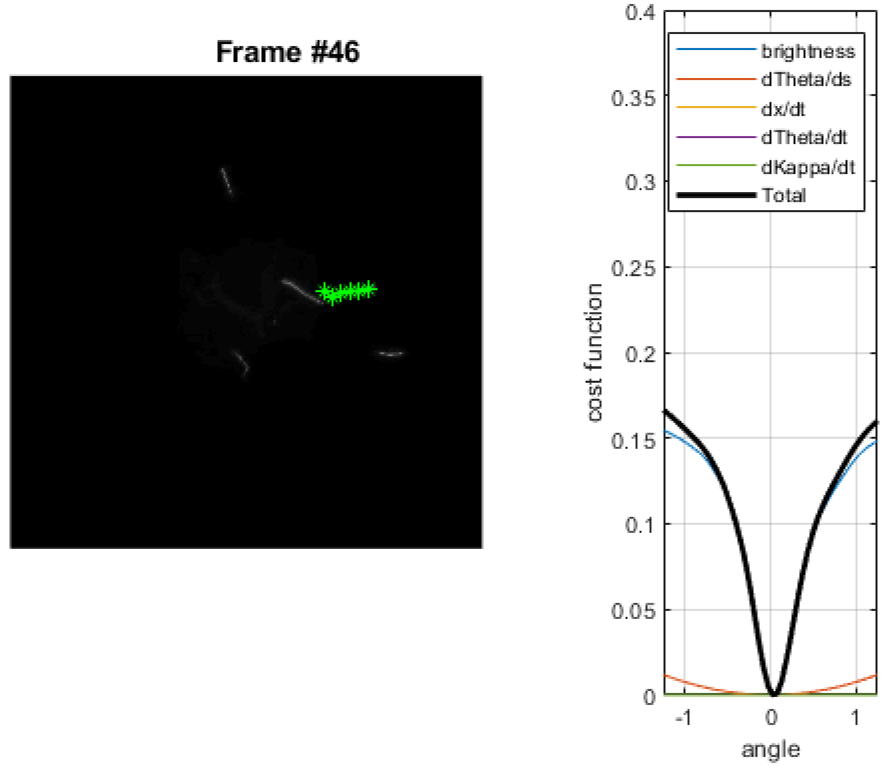

Frame #46
brightness
dTheta/ds
dx/dt
dTheta/dt
dKappa/dt
Total
cost function
angle
0.4
0.35
0.3
0.25
0.2
0.15
0.1
0.05
0
-1
0
1

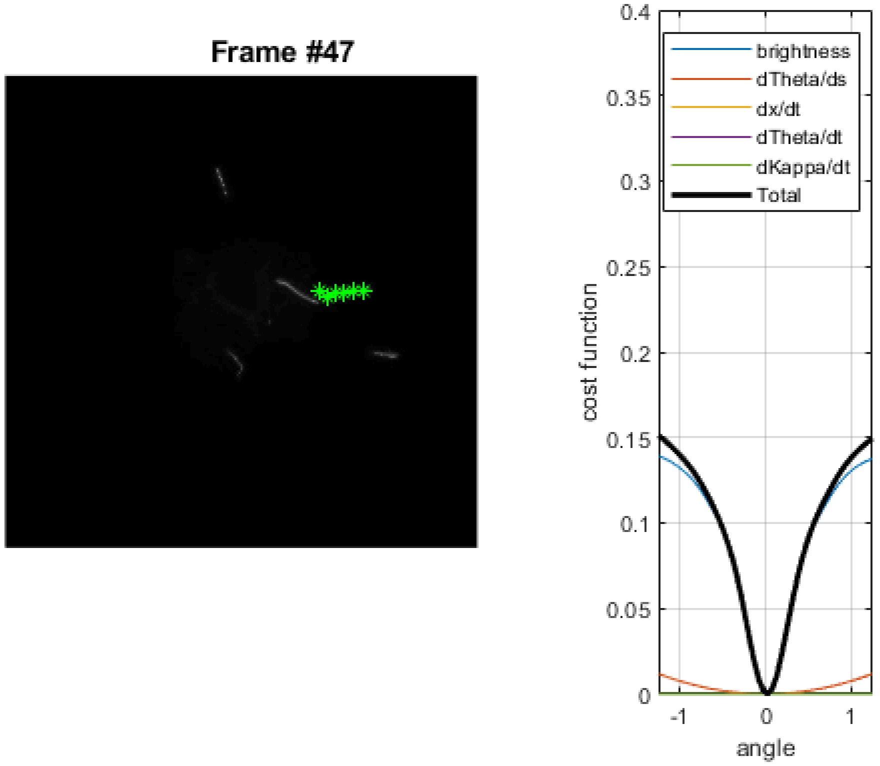
Frame #47
brightness
dTheta/ds
dx/dt
dTheta/dt
dKappa/dt
Total
cost function
angle

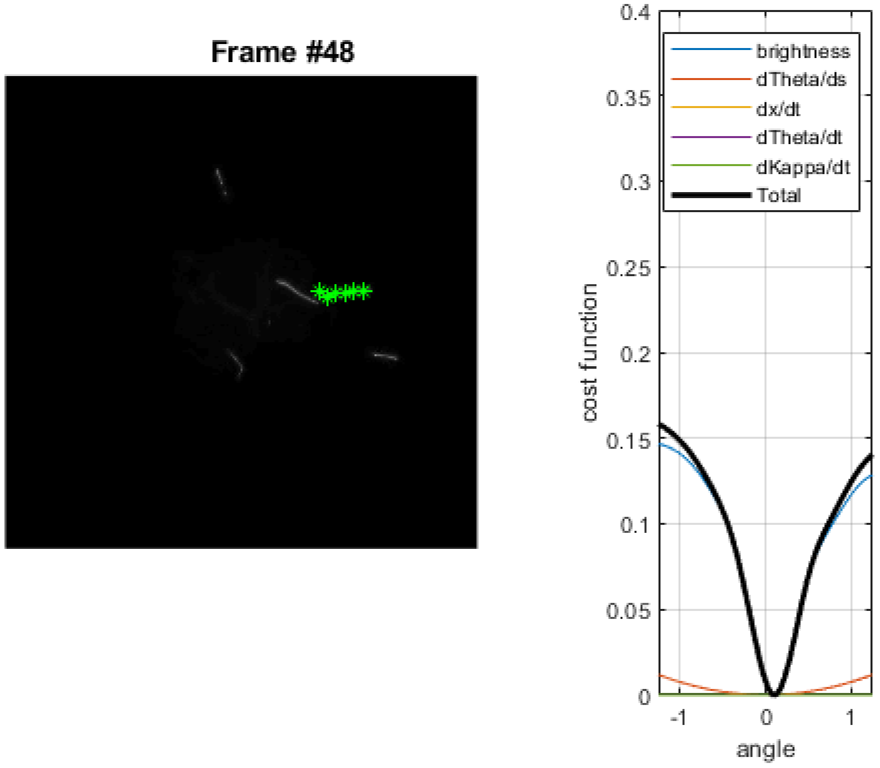
Frame #48
brightness
dTheta/ds
dx/dt
dTheta/dt
dKappa/dt
Total
cost function
angle

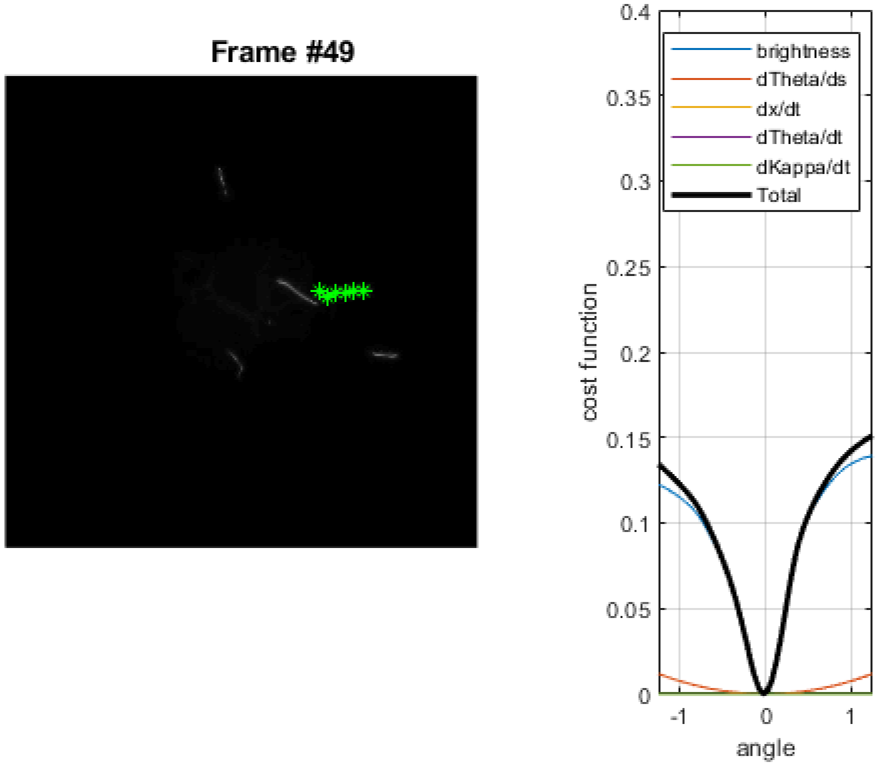
Frame #49
brightness
dTheta/ds
dx/dt
dTheta/dt
dKappa/dt
Total
cost function
angle
0.4
0.35
0.3
0.25
0.2
0.15
0.1
0.05
0
-1
0
1

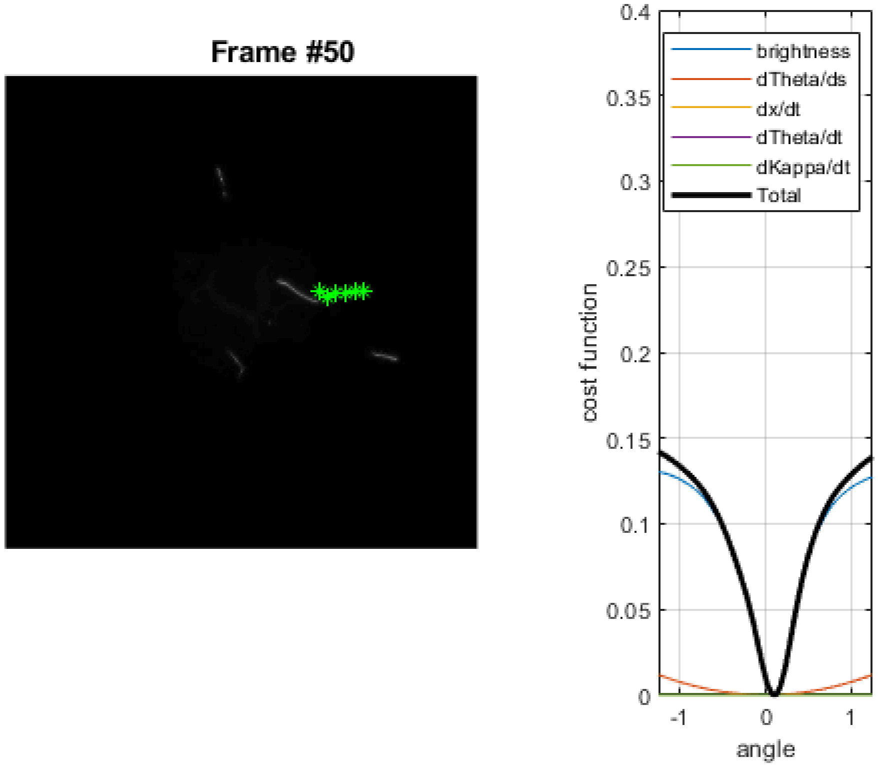
Frame #50
brightness
dTheta/ds
dx/dt
dTheta/dt
dKappa/dt
Total
cost function
angle
0.4
0.35
0.3
0.25
0.2
0.15
0.1
0.05
0
-1
0
1

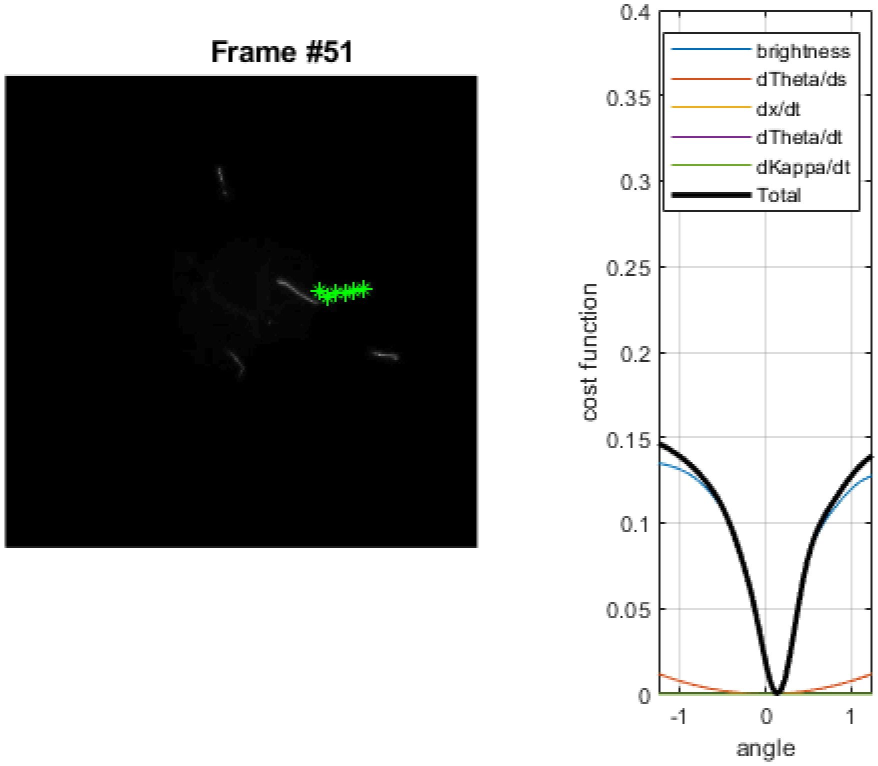
Frame #51
brightness
dTheta/ds
dx/dt
dTheta/dt
dKappa/dt
Total
cost function
angle

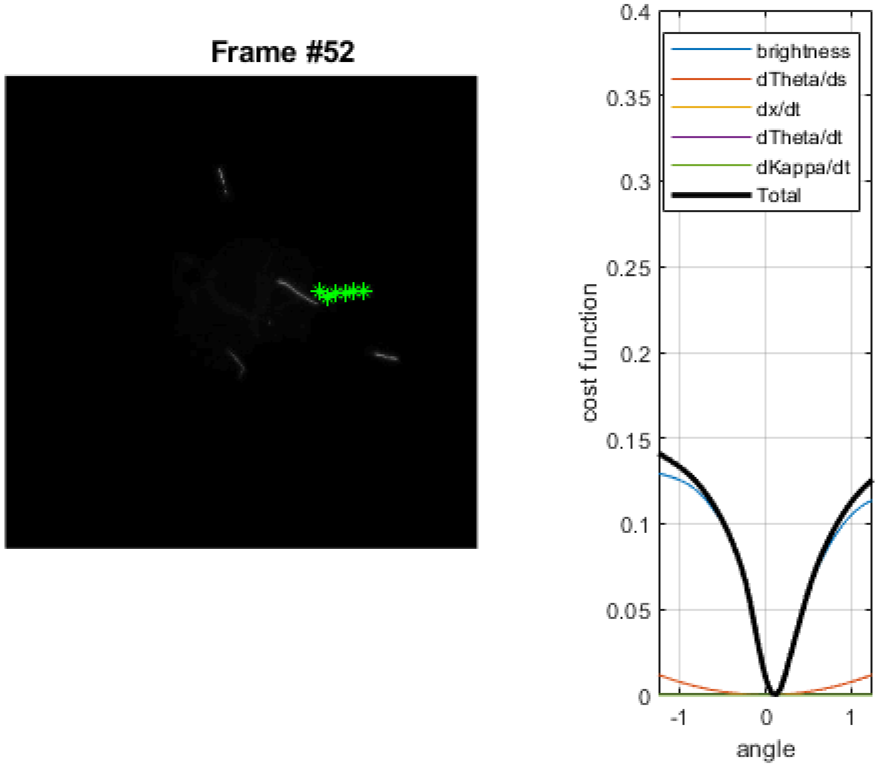
Frame #52
brightness
dTheta/ds
dx/dt
dTheta/dt
dKappa/dt
Total
cost function
angle

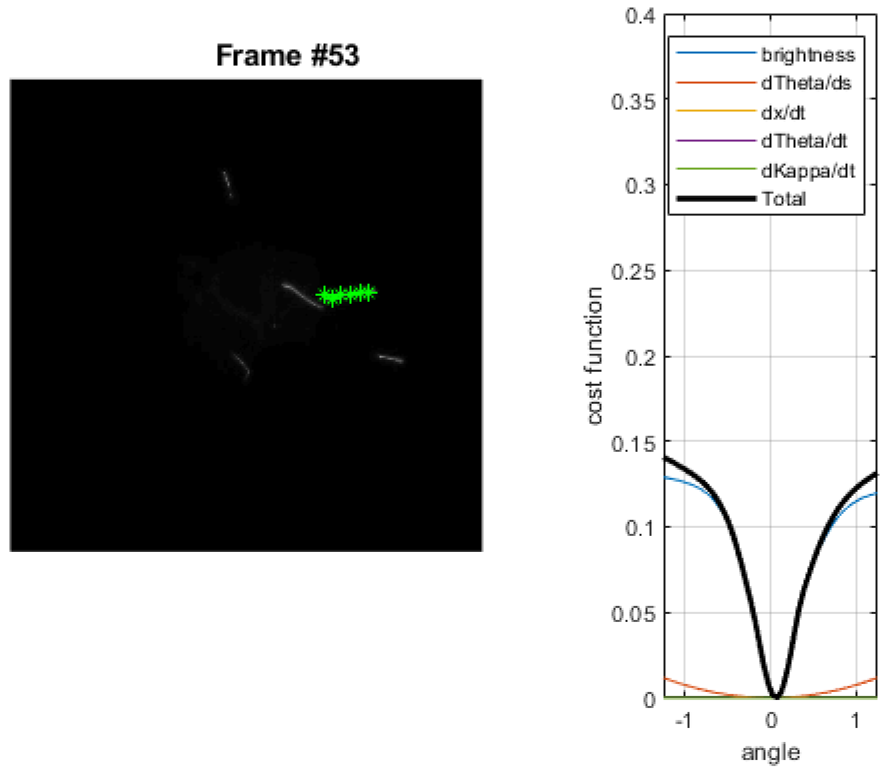
Frame #53
brightness
dTheta/ds
dx/dt
dTheta/dt
dKappa/dt
Total
cost function
angle
0.4
0.35
0.3
0.25
0.2
0.15
0.1
0.05
0
-1
0
1

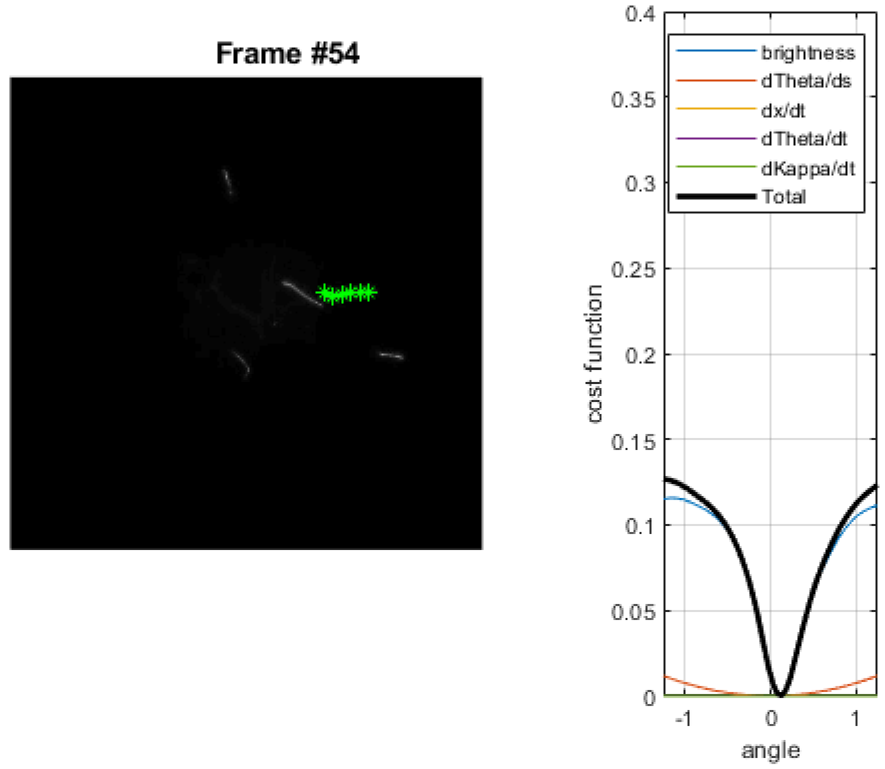
Frame #54
brightness
dTheta/ds
dx/dt
dTheta/dt
dKappa/dt
Total
cost function
angle
0.4
0.35
0.3
0.25
0.2
0.15
0.1
0.05
0
-1
0
1

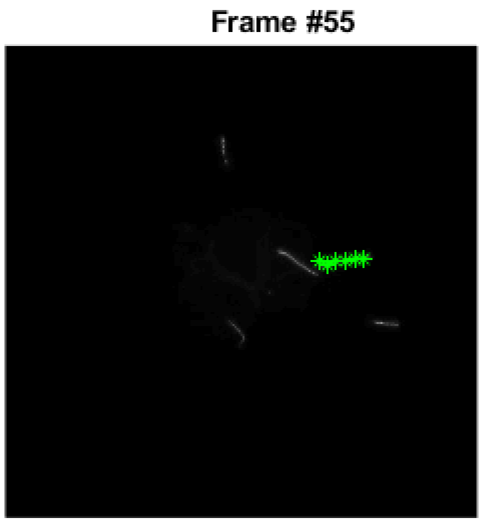
Frame #55

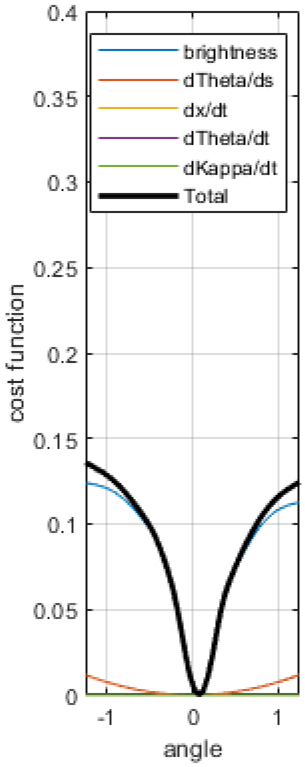
brightness
dTheta/ds
dx/dt
dTheta/dt
dKappa/dt
Total
cost function
angle

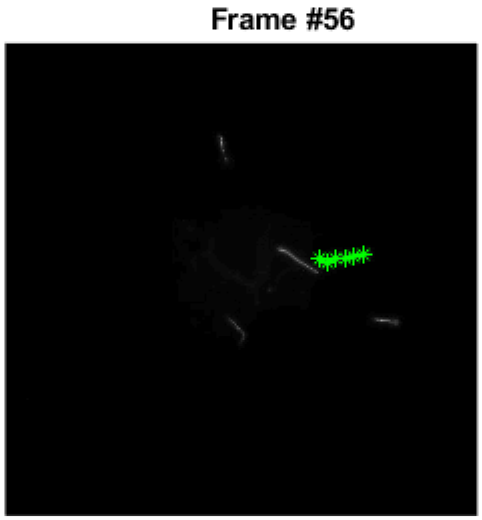
Frame #56

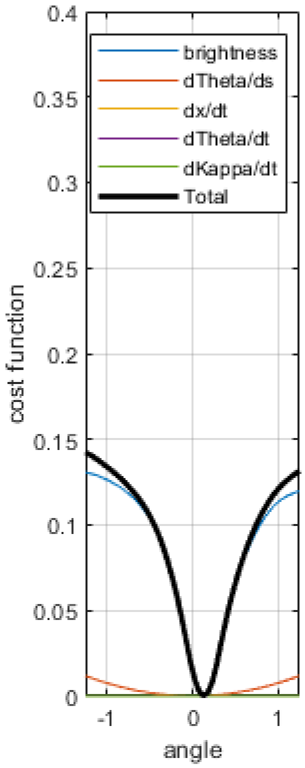
brightness
dTheta/ds
dx/dt
dTheta/dt
dKappa/dt
Total
cost function
angle

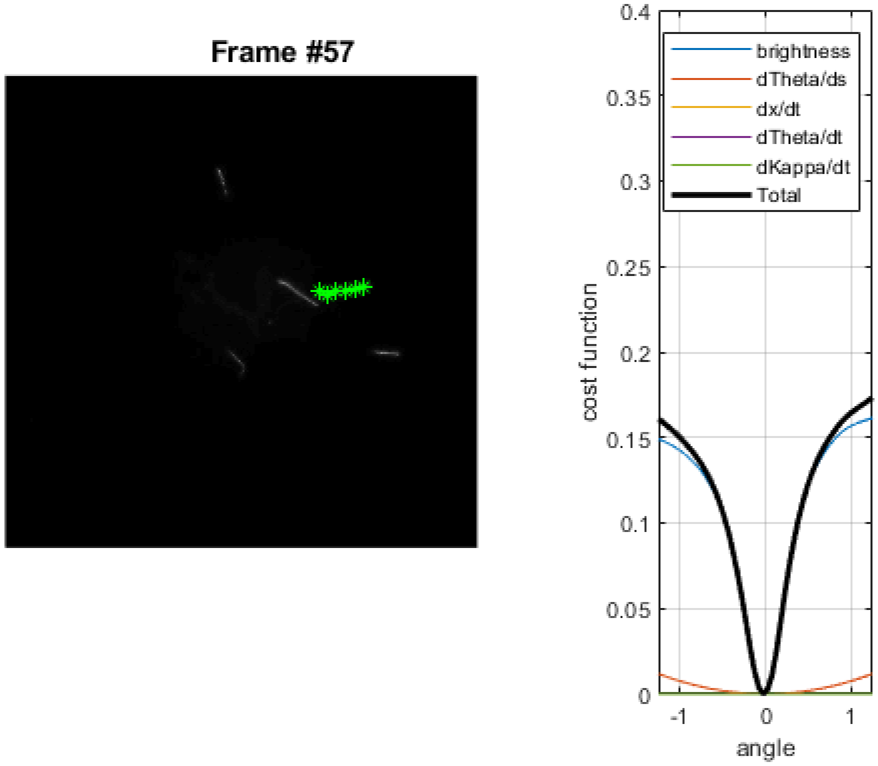
Frame #57
brightness
dTheta/ds
dx/dt
dTheta/dt
dKappa/dt
Total
cost function
angle
0.4
0.35
0.3
0.25
0.2
0.15
0.1
0.05
0
-1
0
1

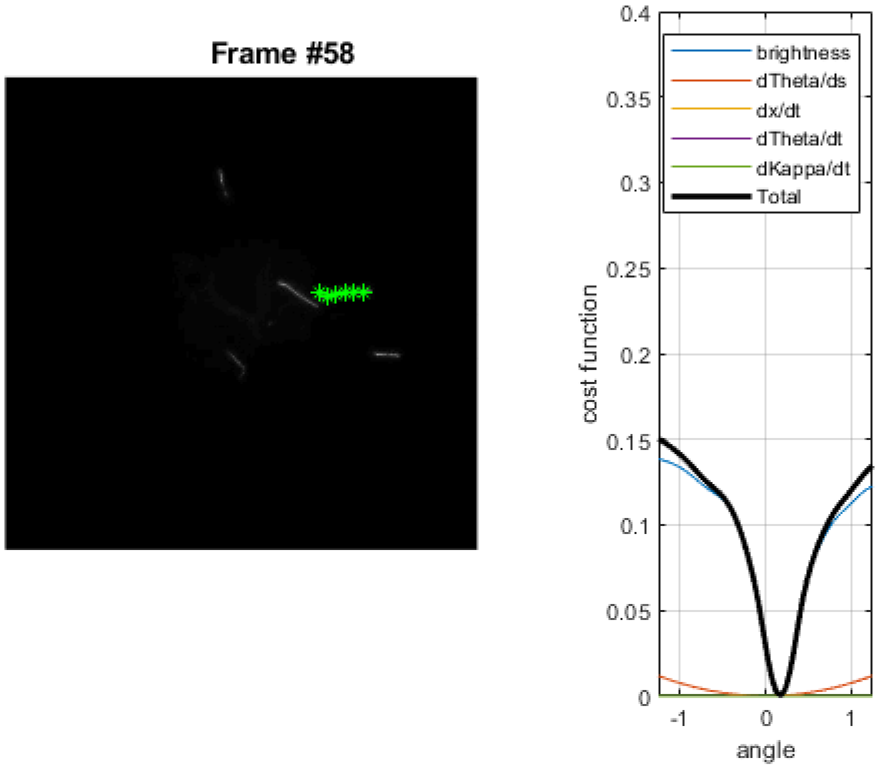
Frame #58
brightness
dTheta/ds
dx/dt
dTheta/dt
dKappa/dt
Total
cost function
angle
0.4
0.35
0.3
0.25
0.2
0.15
0.1
0.05
0
-1
0
1

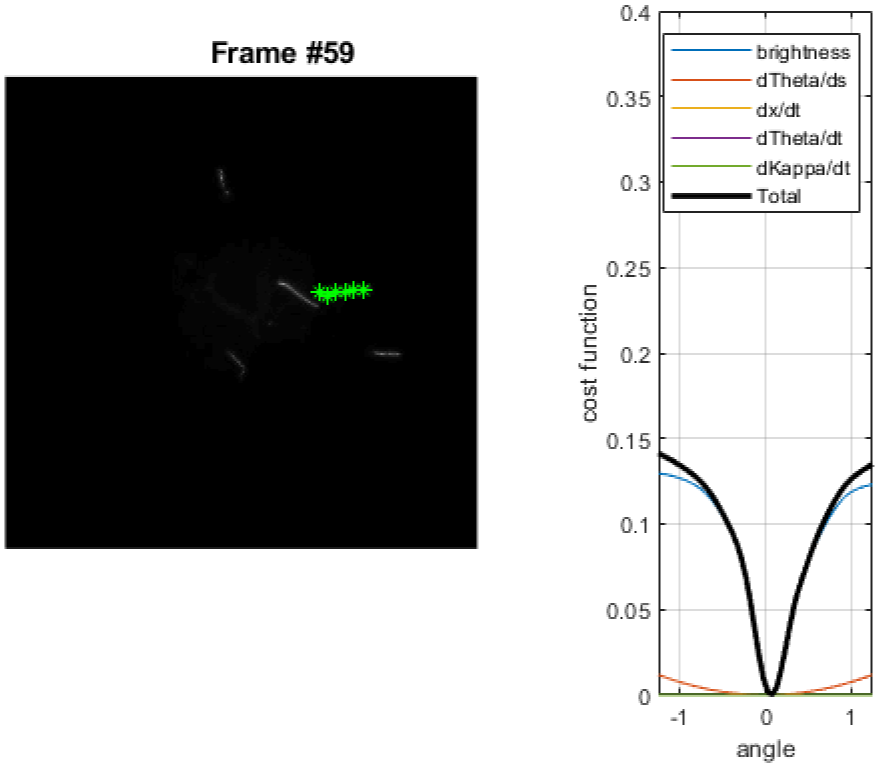
Frame #59
0.4
0.35
0.3
0.25
0.2
0.15
0.1
0.05
0
cost function
-1
0
1
angle
brightness
dTheta/ds
dx/dt
dTheta/dt
dKappa/dt
Total

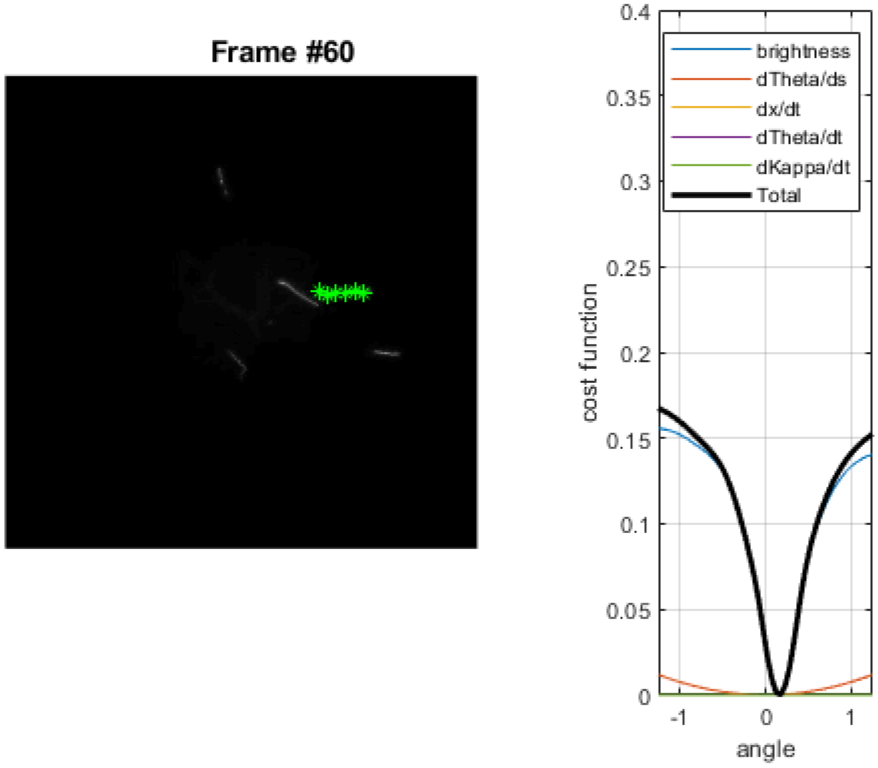
Frame #60
0.4
0.35
0.3
0.25
0.2
0.15
0.1
0.05
0
cost function
-1
0
1
angle
brightness
dTheta/ds
dx/dt
dTheta/dt
dKappa/dt
Total

```
end
thetaArray(1,:) = []; % discard first row - JUNK
if exist('w1','var'), delete(w1), end
toc(startTime)

if showStats
    figure(2)
    subplot(2,1,1)
    relCostArray = diag(sum(costArray,2))\costArray;
    plot(1:numFrames,relCostArray)
    legend('brightness','dTheta/ds','dX/dt','dTheta/dt','dKappa/dt')
    xlabel('frame number')
    ylabel('relative penalty')
    title('relative penalties')
    grid

    subplot(2,1,2)
    plot(totalCost)
    mu = mean(totalCost(2:end));
    s = 2*std(totalCost(2:end));
    line([1 numFrames], [mu mu],'LineStyle','--');
    line([1 numFrames], [mu+s mu+s],'Color','r');
    line([1 numFrames], [mu-s mu-s],'Color', 'green');
    % yl = ylim;
    % ylim([0 yl(2)])
    xlabel('frame number')
    ylabel('total cost')
    title('total cost')
    grid
end



% output to struct
Out.fileName = fileName;
Out.umpp = umpp;
Out.length = arcLenPix*umpp;
Out.dt = dt;

Out.Data.thetaArray = thetaArray;
Out.Data.xArray = squeeze(pointArray(:,1,:));
Out.Data.yArray = squeeze(pointArray(:,2,:));
Out.Data.arcLengthPix = arcLenPix;

Out.Stats.totalCost = totalCost;
Out.Stats.costArray = costArray;
Out.Stats.numFrames = numFrames;
Out.Stats.numPoints = n+1; % n is number of SEGMENTS

FileInfo.firstFrame = firstFrame;
FileInfo.lastFrame  = lastFrame;
FileInfo.fileName = fileName;
FileInfo.line1 = line1;
```

```
Out = postProcTrace(Out);
%Out = rollKappaMax(Out);
Out = findWaveLength(Out);

if showPlot
    %if ~isnan(Out.PP.freq)
        %ntr = ceil(1/Out.dt/Out.PP.freq);
    %else
        ntr = numFrames;
    %end
    figure(3)
    % plotPointArray(pointArray)
    % plotThetaArray(Out.PP.thetaRecon, ntr)
    plotThetaArray(Out.Data.thetaArray, ntr)
end

if showTrace2
    figure(4)
    plotTrace(Out)
end
```

```
Elapsed time is 36.768310 seconds.

ans =

  struct with fields:

    fileName: 'E:\2021-2022 Hughes Lab computer backup\Motility Project files
\Motility video images\Converted AVIs & cilia tracing analysis\Low vs high
 glucose\022822_low high glucose converted\MAX_HG 11mM 1h-1.avi'
        umpp: 0.2600
      length: 12.9170
          dt: 6.8493
        Data: [1×1 struct]
       Stats: [1×1 struct]
          PP: [1×1 struct]
```

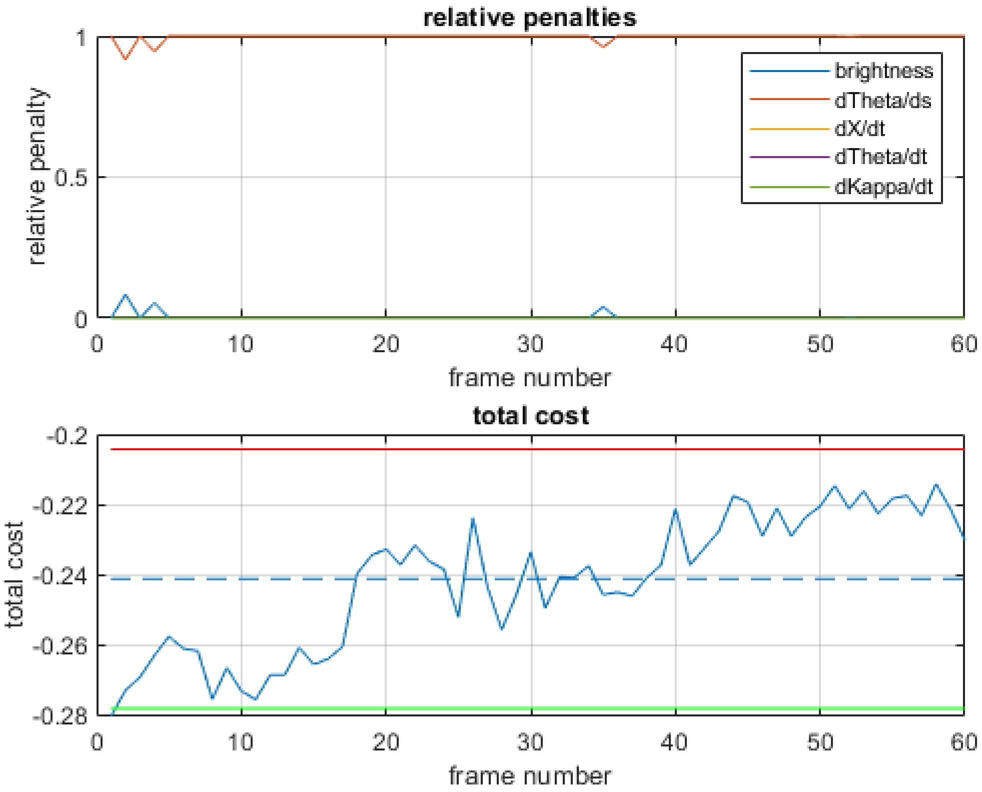
relative penalties
relative penalty
frame number
brightness
dTheta/ds
dX/dt
dTheta/dt
dKappa/dt
1
0.5
0
0
10
20
30
40
50
60
total cost
total cost
frame number
-0.2
-0.22
-0.24
-0.26
-0.28
0
10
20
30
40
50
60

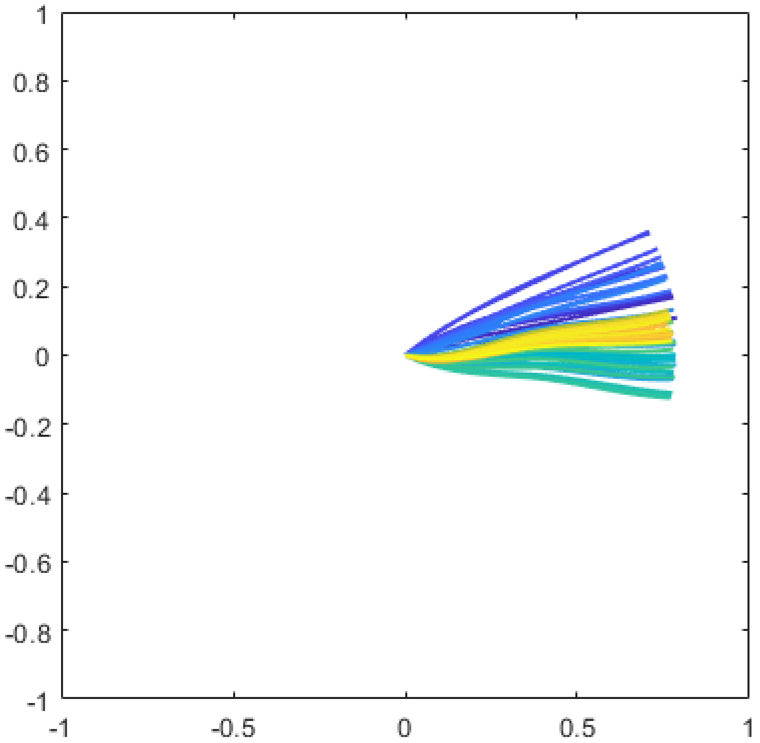
1
0.8
0.6
0.4
0.2
0
-0.2
-0.4
-0.6
-0.8
-1
-1
-0.5
0
0.5
1

Published with MATLAB® R2021b
